# Learning cooking algorithm for solving global optimization problems

**DOI:** 10.1038/s41598-024-60821-0

**Published:** 2024-06-11

**Authors:** S. Gopi, Prabhujit Mohapatra

**Affiliations:** Department of Mathematics, School of Advanced Sciences, Vellore Institute of Technology, Vellore, Tamil Nadu 632 014 India

**Keywords:** Applied mathematics, Computational science

## Abstract

In recent years, many researchers have made a continuous effort to develop new and efficient meta-heuristic algorithms to address complex problems. Hence, in this study, a novel human-based meta-heuristic algorithm, namely, the learning cooking algorithm (LCA), is proposed that mimics the cooking learning activity of humans in order to solve challenging problems. The LCA strategy is primarily motivated by observing how mothers and children prepare food. The fundamental idea of the LCA strategy is mathematically designed in two phases: (i) children learn from their mothers and (ii) children and mothers learn from a chef. The performance of the proposed LCA algorithm is evaluated on 51 different benchmark functions (which includes the first 23 functions of the CEC 2005 benchmark functions) and the CEC 2019 benchmark functions compared with state-of-the-art meta-heuristic algorithms. The simulation results and statistical analysis such as the *t*-test, Wilcoxon rank-sum test, and Friedman test reveal that LCA may effectively address optimization problems by maintaining a proper balance between exploitation and exploration. Furthermore, the LCA algorithm has been employed to solve seven real-world engineering problems, such as the tension/compression spring design, pressure vessel design problem, welded beam design problem, speed reducer design problem, gear train design problem, three-bar truss design, and cantilever beam problem. The results demonstrate the LCA’s superiority and capability over other algorithms in solving complex optimization problems.

## Introduction

The optimization technique involves finding a scenario that minimises or maximises an objective function while fulfilling a predetermined set of constraints. This case is known as the optimal solution, and it is often explored through an exponential collection of candidate solutions requiring highly expensive execution time. Meta-heuristic approximation techniques have been developed to help with this practical challenge. Even though these problem-solving methods cannot guarantee that the solution is optimal, they are quite capable of providing solutions that are close to optimal^1–6^. Meta-heuristic algorithms use exploitation and exploration, which represent intensity and diversity, as their two methods for determining the optimal solution. The growth of meta-heuristic algorithms has been influenced by a variety of natural phenomena, including animals, insects, wildlife, birds, living things, plants, biomedical laws, chemical reactions, physics laws, human activities, game mechanics, and other natural biological processes. In general, meta-heuristic algorithms may be divided into five categories: evolutionary-based optimization algorithms, swarm-based optimization algorithms, chemistry and physics-based optimization algorithms, game-based optimization algorithms, and human-based optimization algorithms.

The modelling of biological sciences and genetics and the use of evolutionary operators like natural selection are the basis of evolutionary-based optimization algorithms^7^. One of the first evolutionary-based optimization algorithm, the genetic algorithm (GA)^8^, has been developed using selection, crossover, and mutation sequence operators and a model of the reproductive process. Another popular evolutionary-based optimization algorithm called differential evolution (DE)^9^ has been developed as a powerful and quick method to solve problems in continuous spaces and has a strong capacity to optimize non-differentiable nonlinear functions. Some other algorithms in this group, such as cultural algorithms (CAs)^10^, Biogeography-Based Optimizer (BBO)^11^, invasive tumor growth (ITGO)^12^, and learner performance behaviour (LPB)^13^. The development of swarm-based optimization algorithms is focused on simulating the natural behaviours of creatures such as animals, insects, ocean animals, plants, and other living things. One of the most commonly used swarm-based algorithms is Particle Swarm Optimization (PSO)^14^, which takes its inspiration from the reasonable behaviour of fish and birds. The Grey Wolf optimization (GWO)^15–17^ has been created using hierarchical leadership behaviour modelling as well as grey wolf hunting tactics. Ant colony optimization (ACO)^18^ was created by modelling the behaviour of ant swarms in order to determine the shortest route between a food source and a nest. The humpback whales use of bubble nets for hunting served as inspiration for the Whale optimization algorithm (WOA)^19^. Tunicate Swarm algorithm (TSA)^20^, Crow Search Algorithm (CSA)^21^, Raccoon optimization algorithm (ROA)^22^, Tree seed algorithm (TSA)^23^, Marine predators algorithm (MPA)^24^, Capuchin search algorithm (CapSA)^25^, Chameleon Swarm algorithm (CSA)^26^, and Aquila optimizer (AO)^27^ are other swarm-based algorithms^28–35^.

Based on the modelling of several physics phenomena and laws, optimization algorithms with such a physics-based algorithm have been developed. The Simulated Annealing (SA)^36^, one of the first algorithms in this category, was inspired by the modelling of the annealing process in metallurgical cooling and melting processes. The water cycle algorithm (WCA)^37^ simulates the evaporation of water from the ocean, cloud formation, rainfall, river creation, and overflow of water from pits, all of which are inspired by the natural water cycle. A gravitational search algorithm (GSA)^38^ has been developed as a result of simulations of the gravitational force that objects exert on one another at various distances. Other physics-based algorithms are atom search optimization (ASO)^39^, multi-verse optimizer (MVO)^40^, Electromagnetic field optimization (EFO)^41^, nuclear reaction optimization (NRO)^42^, optics inspired optimization (OIO)^43^, Equilibrium optimizer (EO)^44^, Archimedes Optimization Algorithm (AOA)^45^, and Lichtenberg Algorithm (LA)^46^. Chemistry-based algorithms have been developed with chemical reactions as inspiration. One of the most famous chemistry-based algorithms is chemical-reaction-inspired meta-heuristic for optimization^47^. Chemical reaction optimization (CRO)^48^ is a recently developed meta-heuristic for optimization that takes inspiration from the nature of chemical reactions. A natural process of changing unstable molecules into stable molecules is called a chemical reaction. Another chemistry-based algorithm is artificial chemical reaction optimization algorithm (ACROA)^49^.

Game-based algorithms have been created using simulations of the rules governing various sports and the actions of players, trainers, and other participants. The Volleyball premier league (VPL)^50^ algorithm’s major concept has been to create modelling contests for the volleyball league, while the football game-based optimization (FGBO)^51^ algorithm’s main idea was to create modelling competitions for the football league. The Puzzle Optimization Algorithm (POA)^52^ was developed mostly as a result of the players’ strategy and talent in creating puzzle components. The primary inspiration for the Tug-of-War Optimization (TWO)^53^ technique was the players’ collective effort throughout the game. The introduction of human-based algorithms is based on the mathematical simulation of various human activities that follow an evolutionary process. The most well-known human-based algorithm is called teaching-learning-based optimization (TLBO)^54^, and it has been created by simulating the conversation and interactions between a teacher and students in a classroom. Doctor and patient optimization (DPO)^55^ algorithm has been made with interactions between doctors and patients, such as preventing illness, getting check-ups, and getting treatment, in mind. Creating Poor and rich optimization (PRO)^56^ has been primarily motivated by the economic activities of the rich and poor in society. In order to be successful, human mental search (HMS)^57^ has been created by simulating human behaviour on online auction marketplaces. Other human-based algorithms are Tabu Search (TS)^58,59^, Imperialist Competitive Algorithm (ICA)^60^, colliding bodies optimization (CBO)^61^, Mine Blast Algorithm (MBA)^62^, seeker optimization algorithm (SOA)^63^, group counseling optimization (GCO)^64,65^ algorithm, harmony search (HS)^66^, League Championship Algorithm (LCA)^67^, Coronavirus herd immunity optimizer (CHIO)^68^, and Ali Baba and the Forty Thieves (AFT)^69^.

In recent years, meta-heuristic algorithms are applied for solving complex problems in different applications such as optimization of weight and cost of cantilever retaining wall^70^, multi-response machining processes^71^, symbiosis organisms search for global optimization and image segmentation^72^, human social learning intelligence^73^, nanotubular halloysites in weathered pegmatites^74^, numerical optimization and real-world applications^75^, convergence analysis^76^, higher Dimensional Optimization Problems^77^, non-dominated sorting advanced^78^, Lagrange Interpolation^79^. LCA is quite different from the existing meta-heuristic algorithms although it belongs to the category of human-based meta-heuristics. The major difference between LCA and them is its particular human-based background. LCA is inspired by observing how mothers and children prepare food. Another important difference is exploration and exploitation. The proposed LCA algorithm works in two phases such as (i) children learn from their mothers and (ii) children and mothers learn from a chef. The exploration is established through Phase 1 of the algorithm when the children learn from their mothers. In the same way, the exploitation is established through Phase 2 of the algorithm, when the children and mother learn from the chef. Therefore, considering these mentioned factors, there are significant differences between LCA and the existing meta-heuristic algorithms. Table 1 shows the comparative assessment between the proposed LCA algorithm and other meta-heuristic algorithms that have been analyzed in terms of algorithm search mechanisms.

**Table 1:** The difference between the proposed LCA algorithm and existing meta-heuristic algorithms.

$\text { Algorithm}$	$\text { Category }$	$\text {Inspiration}$
Genetic algorithm (GA)^8^	Evolutionary-based	Evolutionary concepts
Differential evolution (DE)^9^	Evolutionary-based	Darwin’s theory of evolution
Learner performance behaviour (LPB)^13^	Evolutionary-based	Distribution of students in different departments at a university
Particle swarm algorithm (PSO)^14^	Swarm-based	Social behavior of bird swarms
Tunicate swarm algorithm (TSA)^20^	Swarm-based	The jet propulsion and swarm behaviors of tunicates
Salp swarm algorithm (SSA)^80^	Swarm-based	Foraging behavior of the salps
Grey Wolf optimizer (GWO)^15^	Swarm-based	Hunting behaviour of the grey wolfs
Whale optimization algorithm (WOA)^19^	Swarm-based	Social behavior of the humpback whales
Golden Jackal optimization (GJO)^81^	Swarm-based	Hunting behaviour of the golden jackals
Beluga Whale optimization (BWO)^82^	Swarm-based	Social behavior of beluga whales
Harris Hawks Optimizer (HHO)^83^	Swarm-based	Cooperative behavior and stalking style of Harris’s hawks
Mountain Gazelle optimizer (MGO)^84^	Swarm-based	The social life and hierarchy of wild mountain gazelles
Sand cat swarm optimization (SCSO)^85^	Swarm-based	Social behavior of the sand cat
Simulated annealing (SA)^36^	Physics-based	Annealing process in metallurgy
Multi-Verse optimizer (MVO)^40^	Physics-based	Multi-verse theory
Equilibrium optimizer (EO)^44^	Physics-based	Physics-based source and sink models
Teaching-learning-based optimization (TLBO)^54^	Human-based	Teaching and learning in a classroom
Mountaineering team based optimization (MTBO)^86^	Human-based	Social performance and cooperation of humans by considering natural phenomena
Seeker optimization algorithm (SOA)^63^	Human-based	The action of human randomized search
Ali Baba and the Forty Thieves (AFT)^69^	Human-based	The tale of Ali Baba and the forty thieves
Coronavirus herd immunity optimizer (CHIO)^68^	Human-based	Herd immunity concept to respond to COVID-19
Learning cooking algorithm (LCA)	Human-based	The cooking activity of humans

The existing meta-heuristic algorithms have some flaws, concerns, and issues. For example, the Harris Hawk Optimization (HHO) algorithm^83^ performs well at solving standard benchmark problems while failing miserably at complex problems such as CEC 2017 and real-world problems. As a result, in order to solve complex problems and functions, the performance of this algorithm needs to be improved. The poor and rich algorithm (PRO)^56^ was recently developed and configured to perform well on a few simple and old test functions while failing to solve new and complex test functions such as CEC 2017. As a result, in order to solve complex problems and functions, the algorithm has to be improved. The mechanism of well-known algorithms such as the grey wolf optimizer (GWO)^15^ and the whale optimization algorithm (WOA)^19^ is very similar, with the main difference being the search range. Here, a critical question arises: What is required to offer and develop new algorithms in the presence of well-known algorithms like those stated above? According to the No Free Lunch (NFL)^87^ theorem, no optimization algorithm can solve all optimization problems. According to the NFL, an algorithm’s ability to successfully address one or more optimization problems does not guarantee that it will do so with others, and it may even fail. As a result, it is impossible to say that a particular optimization algorithm is the best approach for all problems. New algorithms can always be developed that are more effective than current algorithms at solving difficult optimization problems. The NFL invites researchers to be inspired to create new optimization algorithms that are better able to address difficult optimization problems. The ideas described in the NFL theorem inspired the authors of this paper to propose a new optimization algorithm namely the Learning Cooking Algorithm (LCA).

In every optimization algorithm, exploration and exploitation play the most important role. So, keeping this in mind, this paper proposes a new human-based algorithm namely the LCA algorithm to maintain a proper balance between exploration and exploitation among optimization algorithms. The LCA algorithm mimics two phases: (i) children learn from their mothers and (ii) children and mothers learn from a chef. The exploration is established through Phase 1 of the algorithm when the children learn from their mothers. For each child, the corresponding mother is chosen by the greedy selection mechanism. This phase helps the algorithm explore the large search space. In the same way, the exploitation is established through Phase 2 of the algorithm, when the children and mother learn from the chef. The chef acts as the global best solution and directs the other swarm particles i.e. the children and mothers to move towards it. The 51 different benchmark functions (which include the first 23 functions of the CEC 2005 benchmark functions) and the CEC 2019 benchmark functions are employed to evaluate the LCA’s capability. Seven well-known algorithms, two top-performing algorithms, and eight recently developed algorithms for solving optimization problems are compared to the performance of the proposed LCA algorithm. This algorithm has also been used to solve seven optimal design problems in order to evaluate the LCA for solving real-life engineering problems. The structure of the paper is designed as follows: “Learning cooking algorithm” describes the inspiration for the Learning Cooking Algorithm (LCA) and the mathematical model for the LCA. In “Simulation studies and results”, simulation studies, results, and discussion are presented. The performance of LCA in solving engineering design problems is evaluated in “LCA for engineering optimization problems”. Conclusions and suggestions for further study of this paper are provided in “Conclusion”.

## Learning cooking algorithm

In this section, the learning cooking algorithm (LCA) is proposed, followed by a discussion of its mathematical modelling.

### Inspiration

Cooking is the process of exposing food to heat. All of the methods constitute cooking, regardless of whether the food is baked, fried, sauteed, boiled, or grilled. According to the evidence, our ancestors began cooking over an open fire some 2 million years ago. Although devices like microwaves, toasters, and stovetops are extensively utilized, some foods are still cooked over an open flame. There are numerous ways to cook, but the majority of them have their origins in the past. These include boiling, steaming, braising, grilling, barbecuing, roasting, and smoking. Steaming is a more recent innovation. Different cooking techniques require varying levels of heat, moisture, and time. First of all, without being cooked, certain foods are not safe to eat. Cooking not only heats food but also has the potential to eliminate dangerous microorganisms. Because they are more likely to carry bacteria while they are raw, meats must be cooked to a specific temperature before eating. Cooking is a learning process in which a beginner (a child) learns to cook from the mother, and then children and mothers learn to cook from the cooking expert by watching television, YouTube, and other social media platforms. This study “Learning Cooking Algorithm,” is divided into two phases: (i) children learn from their mothers and (ii) children and mothers learn from a chef. This idea is similar to meta-heuristic algorithms, in which the problem’s best candidate solution is selected as the algorithm’s final output after multiple initial candidate solutions are improved through an iterative process.

### Mathematical model of LCA

LCA is a population-based optimization algorithm that includes cooking learners (children), mothers, and chefs. LCA members are candidate solutions to the problem, as it is modelled by a population matrix in Eq. (1). Equation (2) is used to randomly initialize these members’ positions at the beginning of implementation.

1 $$\begin{aligned} C= & {} \begin{bmatrix} C_1\\ \vdots \\ C_i\\ \vdots \\ C_N \end{bmatrix}_{N\times m}= \begin{bmatrix} c_{11} &{} \cdots &{} c_{1j}&{}\cdots &{}c_{1m}\\ \vdots &{}\ddots &{}\vdots &{}\ddots &{}\cdots \\ c_{i1}&{} \cdots &{} c_{ij}&{}\cdots &{}c_{im}\\ \vdots &{}\ddots &{}\vdots &{}\ddots &{}\cdots \\ c_{N1}&{} \cdots &{} c_{Nj}&{}\cdots &{}c_{Nm} \end{bmatrix}_{N\times m}, \end{aligned}$$

2 $$\begin{aligned} c_{i,j}= & {} rand(UB_j-LB_j)+LB_j, i=1,2,...,m, \end{aligned}$$

where *C* is the LCA population, $C_i$ is the $i{th}$ candidate solution, $c_{i,j}$ is the value of the $j^{th}$ variable determined by the $i{th}$ candidate solution, the value *N* represents the size of the LCA population, *m* is the number of problem variables, the value of *rand* is chosen at random from the range $\left[ 0,1\right]$, the upper and lower limits of the $j{th}$ problem variable are denoted as ${UB}_j$ and ${LB}_j$, respectively. Here, in the LCA algorithm the food items represent the problem variables. The children and mothers try to learn different types of cuisines such as Mexican, Italian, Indian, American cuisines from the chefs around the world.

The objective function values are represented by the vector in Eq. (3).

3 $$\begin{aligned} F= \begin{bmatrix} F_1\\ \vdots \\ F_i\\ \vdots \\ F_N \end{bmatrix}_{N\times 1}= \begin{bmatrix} F(C_1)\\ \vdots \\ F(C_i)\\ \vdots \\ F(C_N) \end{bmatrix}_{N\times 1}, \end{aligned}$$

where the objective functions are represented by the vector *F* and $F_i$ represented the of objective function delivered by the $i{th}$ candidate solution.

The values for the objective function are the most important things used to judge the quality of candidate solutions. On the basis of comparisons of the values of the objective function, the member of the population with the best value is referred to as the “best member of the population (Cbest).” Each iteration improves and updates the candidate solutions, so the best member must also be updated. The methodology used to update candidate solutions is the main difference between meta-heuristic optimization algorithms. In LCA, candidate solutions are updated in two main phases: (i) children learn from their mothers and (ii) children and mothers learn from a chef.

#### Phase 1: children learn from their mothers (exploration)

**Figure 1:**
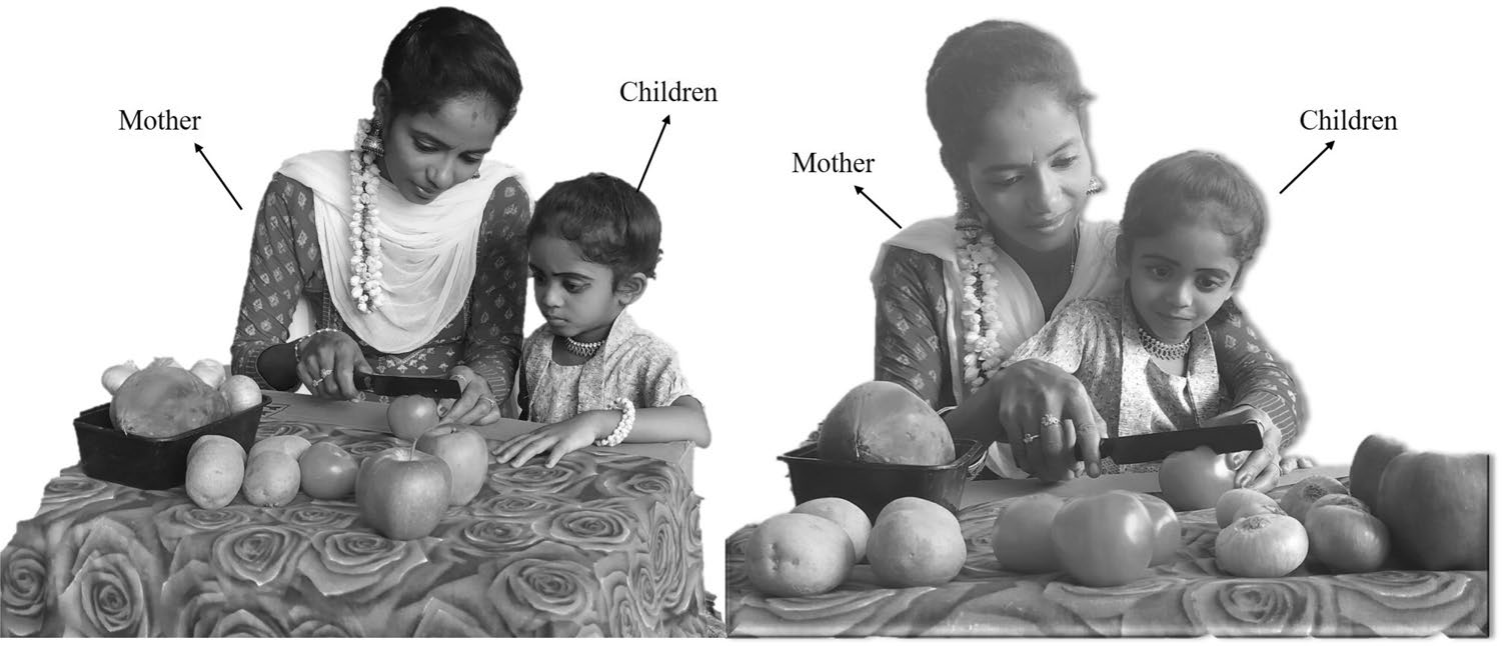
Children learn from their mothers.

 The first stage of the LCA update is based on the choice of the mother by the children and then the teaching of cooking by the selected mother to the children (Fig. 1). The selection of mothers is done by choosing a number of the best members from the whole population. The number of mothers is denoted by $N_{MO}$ which is decided using the formula $N_{MO} =\lfloor 0.1\cdot N\cdot (1-\frac{t}{T} )\rfloor$. After choosing the mother and trying to learn how to cook, children in the population will move to different places in the search space. This will improve the LCA’s exploration capabilities in the global search for it and the identification of the optimal location. The exploratory capability of this algorithm is therefore demonstrated by this stage of the LCA. Equation (4) says that the $N_{MO}$ members of the LCA population are chosen as mothers by comparing the values of the objective function at each iteration.

4 $$\begin{aligned} MO= \begin{bmatrix} MO_1\\ \vdots \\ MO_i\\ \vdots \\ MO_{N_{MO}} \end{bmatrix}_{N_{MO}\times m}= \begin{bmatrix} MO_{11} &{} \cdots &{} MO_{1j}&{}\cdots &{}MO_{1m}\\ \vdots &{}\ddots &{}\vdots &{}\ddots &{}\cdots \\ MO_{i1}&{} \cdots &{} MO_{ij}&{}\cdots &{}MO_{im}\\ \vdots &{}\ddots &{}\vdots &{}\ddots &{}\cdots \\ MO_{N_{MO}1}&{} \cdots &{} MO_{N_{MO}j}&{}\cdots &{}MO_{N_{MO}m} \end{bmatrix}_{N_{MO}\times m} \end{aligned}$$

where *MO* is the matrix of mothers, ${MO}_{i}$ is the $i{th}$ mothers, ${MO}_{i,j}$ is the $j{th}$ dimension, and $N_{MO}$ is the number of mothers, *t* represents the current iteration and the maximum number of iterations is *T*.

The new location for each member is first determined by using Eq. (5) in accordance with the mathematical modelling of this LCA phase. Equation (6) shows that the new location takes the place of the old one if the value of the objective function increases.

5 $$\begin{aligned} c_{i,j}^{P1}= & {} {\left\{ \begin{array}{ll} c_{i,j}+rand_{1}\cdot (MO_{{k_i},{j}}-I_{1}\cdot {c_{i,j}}), F_{MO_{k_i}} <F_i; \\ c_{i,j}+rand_{1}\cdot ({c_{i,j}}-I_{1}\cdot {MO_{{k_i},{j}}}), otherwise, \end{array}\right. } \end{aligned}$$

6 $$\begin{aligned} C_{i}= & {} {\left\{ \begin{array}{ll} C_{i}^{P1},F_{i}^{P1}<F_i; \\ C_{i}, otherwise, \end{array}\right. } \end{aligned}$$

where $C_{i}^{P1}$ is the new calculated status for the $i{th}$ candidate solution based on the first phase of LCA, $c_{i,j}^{P1}$ is its $j{th}$ dimension, $F_{i}^{P1}$ is its objective function value, $I_{1}$ is a number chosen at random from the range of $\{1,2\}$, and the value of $rand_{1}$ is a random number between [0, 1], $MO_{{k_i}}$, where $k_i$ is chosen at random from the set $\{1,2,\cdots ,N_{MO} \}$, represents a randomly selected mother to learn the $i{th}$ member, $MO_{{k_i},{j}}$ is its $j^{th}$ dimension, and $F_{MO_{k_i}}$ is its objective function value.

#### Phase 2: children and mother learn from chef (exploitation)

**Figure 2:**
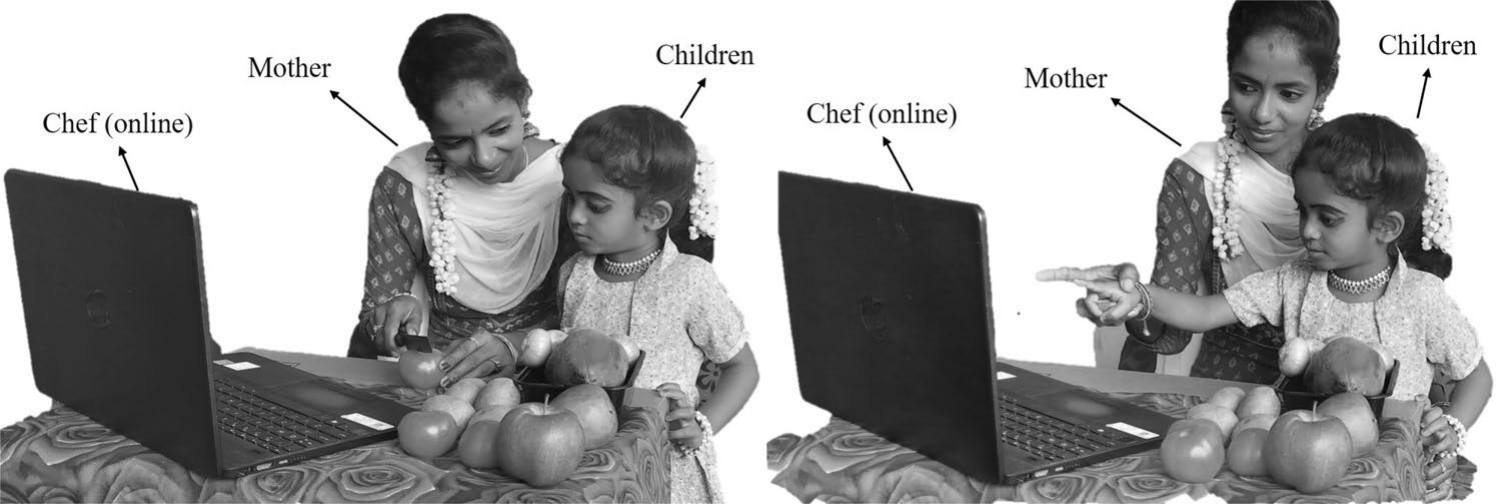
Children and mothers learn from chefs via social media.

 The second stage of the LCA update is based on the children and their mother selecting the chef, followed by watching a YouTube video to learn the chef’s style of cooking (Fig. 2). The selection of chefs is done by choosing a number of the best members from the mother population. The number of chefs is denoted by $N_{Cf}$ which is decided using the formula $N_{Cf} =\lfloor 0.1\cdot N_{MO}\cdot (1-\frac{t}{T})\rfloor$. The population members will move to the local search after selecting the chef and learning about their various cooking techniques. The $N_{Cf}$ members of the LCA population are chosen as chefs in each iteration based on a comparison of the values of the objective function, as given in Eq. (7). This phase demonstrates the power of LCA to exploit global search.

7 $$\begin{aligned} Cf= & {} \begin{bmatrix} Cf_1\\ \vdots \\ Cf_i\\ \vdots \\ Cf_{N_{Cf}} \end{bmatrix}_{N_{Cf}\times m}= \begin{bmatrix} Cf_{11} &{} \cdots &{} Cf_{1j}&{}\cdots &{}Cf_{1m}\\ \vdots &{}\ddots &{}\vdots &{}\ddots &{}\cdots \\ Cf_{i1}&{} \cdots &{} Cf_{ij}&{}\cdots &{}Cf_{im}\\ \vdots &{}\ddots &{}\vdots &{}\ddots &{}\cdots \\ Cf_{N_{Cf}1}&{} \cdots &{} Cf_{N_{Cf}j}&{}\cdots &{}Cf_{N_{Cf}m} \end{bmatrix}_{N_{Cf}\times m}, \end{aligned}$$

where *Cf* is the matrix of chefs, ${Cf}_{i}$ is the $i{th}$ chefs, ${Cf}_{i,j}$ is the $j{th}$ dimension, *t* represents the current iteration and the maximum number of iterations is *T*.

In order to represent this concept mathematically, the new location for each member is determined using Eq. (8). By Eq. (9), this new position replaces the previous one if it enhances the objective function’s value.

8 $$\begin{aligned} c_{i,j}^{P2}= & {} {\left\{ \begin{array}{ll} c_{i,j}+rand_{2}\cdot (Cf_{{k_i},{j}}-I_{2}\cdot MO_{{k_i},{j}})+rand_{3}\cdot (Cf_{{k_i},{j}}-I_{3}\cdot {c_{i,j}}), F_{Cf_{k_i}} <F_i; \\ c_{i,j}+rand_{2}\cdot (MO_{{k_i},{j}}-I_{2}\cdot {Cf_{{k_i},{j}}})+rand_{3}\cdot ({c_{i,j}}-I_{3}\cdot {Cf_{{k_i},{j}}}), otherwise, \end{array}\right. } \end{aligned}$$

9 $$\begin{aligned} C_{i}= & {} {\left\{ \begin{array}{ll} C_{i}^{P2},F_{i}^{P2}<F_i; \\ C_{i}, otherwise, \end{array}\right. } \end{aligned}$$

where $C_{i}^{P2}$ is the new calculated status for the $i^{th}$ candidate solution based on the second phase of LCA, $c_{i,j}^{P2}$ is its $j^{th}$ dimension, $F_{i}^{P2}$ is its objective function value, $I_{2}$, $I_{3}$, are a numbers randomly chosen from the set $\{1,2\}$, the values of $rand_{2}$, $rand_{3}$ are a random numbers between [0, 1], $Cf_{k_i}$, where $k_i$ is chosen at random from the set $\{1,2,\cdots ,N_{Cf} \}$, represents a randomly selected chef to learn the $i^{th}$ member and mother, ${Cf_{{k_i},{j}}}$ is its $j^{th}$ dimension, and $F_{Cf_{k_i}}$ is its objective function value.

#### Repetition procedure, pseudo-Code of LCA and LCA flow chart:

**Figure 3:**
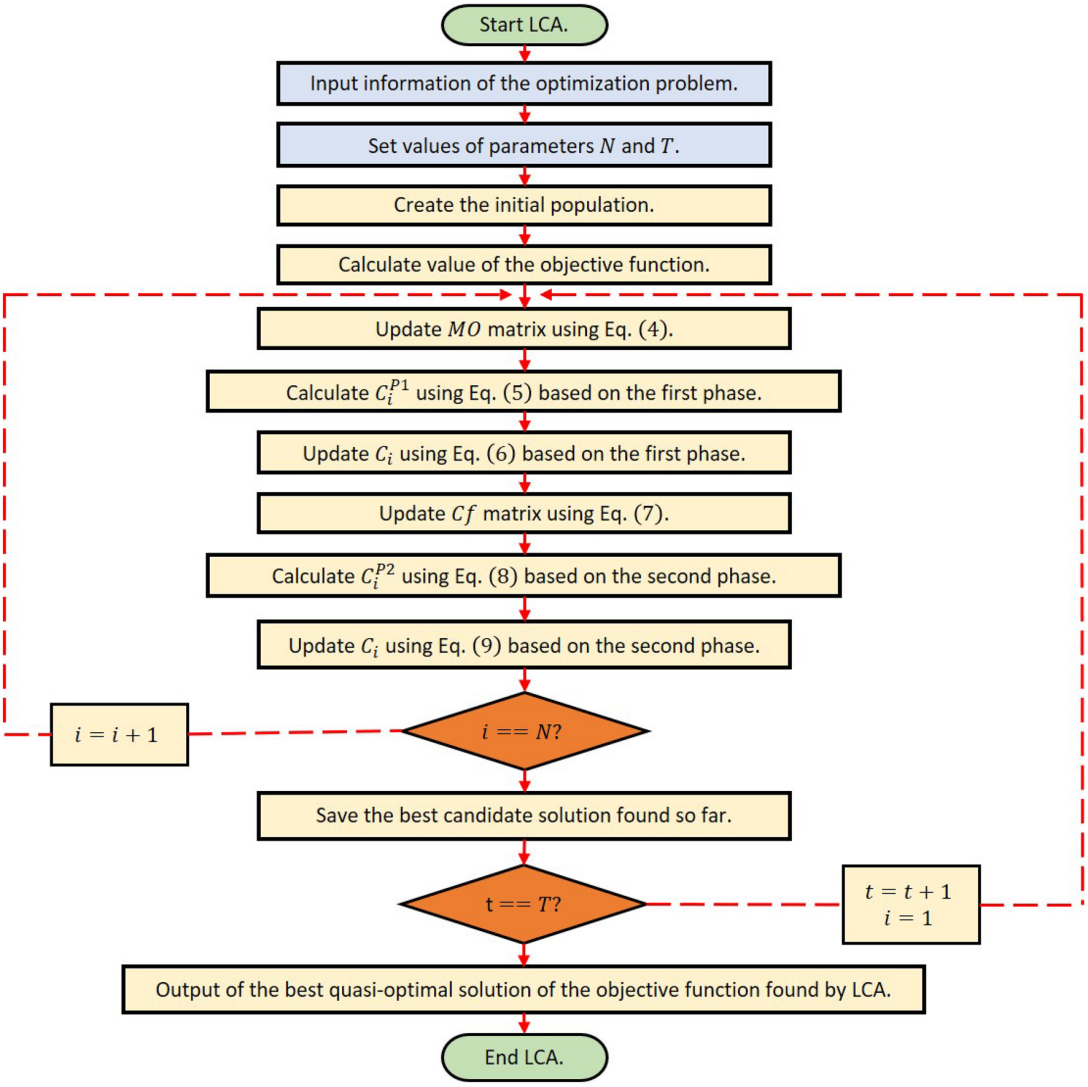
Flowchart of the learning cooking algorithm.

 An LCA iteration is completed after the population members have been updated in accordance with the first and second stages. The algorithm entered the following LCA iteration with the updated population. To complete the maximum number of repetitions, the updation procedure is performed in accordance with the first and second phase stages and in accordance with Eqs. (4) to (9). The best candidate solution that has been recorded during the execution of LCA on the given problem is presented as the solution when LCA has been fully implemented. The proposed LCA algorithm pseudocode is shown in Algorithm 1, and Fig. 3 shows its flowchart.

**Algorithm 1:**
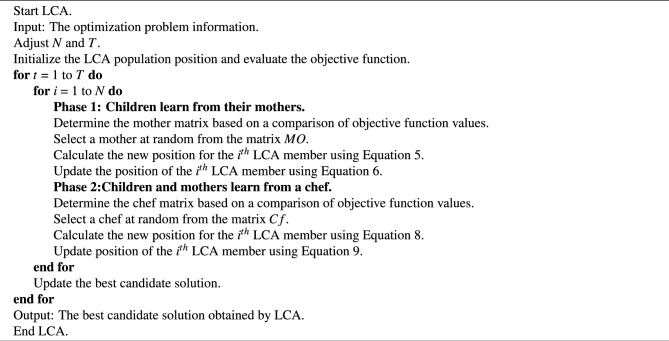
Pseudo-code of the proposed learning cooking algorithm.

### The computational complexity of LCA

The computational complexity of *LCA* is discussed in this subsection. The computational complexity of the preparation and initialization of *LCA* for the problem with the number of members equal to *N* and the problem with the number of decision variables equal to *m* is equal to $O(N \times m)$. The *LCA* members are updated in two stages during each iteration. As a result, the computational complexity of the *LCA* update processes is $O(2N\times m\times T)$, where *T* is the maximum number of algorithm iterations. As a consequence, the total computational complexity of *LCA* is $O(N\times m\times (1+2T))$.

## Simulation studies and results

This section looks at how well the LCA performs in applications involving optimization and how it provides the most effective solutions to these types of problems. In LCA, fifty-one different Benchmark functions (which includes the first 23 functions of the CEC 2005 benchmark functions)^88^ and ten CEC 2019 benchmark functions^89^ are used. The performance of the LCA algorithm is compared with well-known algorithms, top-performance algorithms, modified algorithms, and newly developed algorithms, such as PSO^14^, TSA^20^, SSA^80^, MVO^40^, GWO^15^, WOA^19^, GJO^81,90^, LSHADE^91^, CMAES^92^, IGWO^93^, MWOA^94^, TLBO^54^, MTBO^86^, BWO^82^, HHO^83^, MGO^84^, and SCSO^85^ in order to evaluate the efficiency of the LCA results. For each of the optimization algorithms under evaluation, the size of the population, maximum iterations, and the number of function evaluations (NFEs) are set at 30, 1000, and 30,000, respectively, with 20 independent runs for each function. Table 2 lists the parameter values for each algorithm. The details of the 51 test functions and CEC 2019 test functions are described in Tables 3 and 4. For validation, a parametric and non-parametric statistical analysis such as the mean value of the fitness function (average), standard deviation (std), best, worst, median, Wilcoxon rank-sum test, t-test, rank, Friedman test, and convergence curve of algorithms are used. Optimization algorithms base their performance ranking criteria on the t-test value. The *NA* denotes “Not Applicable” which means that the equivalent algorithm result cannot be compared with other algorithm results. The experiments are performed on Windows 10, Intel Core i3, 2.10GHz, 8.00 GB RAM, and MATLAB R2020b.

**Table 2:** The values are set for the control parameters of the competitor algorithms.

$\text {Algorithm}$	$\text {Parameter}$	$\text {Value}$
${\text {PSO}}$	Cognitive constant (*C*1)	1.5
	Social constant (*C*2)	2
	Local constant (*W*)	0.3
${\text {TSA}}$	Parameter $P_{min}$	1
	Parameter $P_{max}$	4
${\text {SSA}}$	Leader position update probability	0.5
${\text {MVO}}$	Wormhole existence probability (WEP)	$WEP\_Max$ = 1, $WEP\_Min$ = 0.2
${\text {GWO}}$	*l* is a random number	$[- 1, 1]$
	*r* is a random vector	[0, 1]
	Convergence parameter (*a*)	Linear reduction from 2 to 0
${\text {WOA}}$	*l* is a random number	[– 1, 1]
	*r* is a random vector	[0, 1]
	Convergence parameter (*a*)	Linear reduction from 2 to 0
${\text {GJO}}$	Constant value	$c_{1} = 1.5$
	Energy of the prey	$E_{1}$ is linearly decreased from 1.5 to 0
${\text {CMAES}}$	Number of parents	$\mu$= $\lfloor N/2 \rfloor$
	Step size	$\alpha$=2
${\text {LSHADE}}$	Pbest rate	0.11
	Arc rate	1.4
	Memory size	5
${\text {IGWO}}$	*l* is a random number	$[- 1,1]$
	*r* is a random vector	[0, 1]
	Convergence parameter (*a*)	Linear reduction from 2 to 0
${\text {MWOA}}$	*l* is a random number	[– 1,1]
	*r* is a random vector	[0, 1]
	Convergence parameter (*a*)	Linear reduction from 2 to 0
${\text {TLBO}}$	Teacher factor	[1, 2]
	Random number	[0, 1]
$${\text {MTBO}}$$	The scaling factors	$$Li=(0.25+0.25*rand) $$
		$$Ai=(0.75+0.25*rand) $$
		$$Mi=(0.75+0.25*rand) $$
${\text {BWO}}$	The probability of whale fall decreased at the interval $W_{f}$	[0.1, 0.05]
${\text {HHO}}$	Probability thresholds of escaping, escaping energy	0.5, 0.5
${\text {MGO}}$	The population size	$N=30$
	Maximum number of iterations	$T=1000$
${\text {SCSO}}$	Sensitivity range $(r_{G})$	[2, 0]
	Phases control range (*R*)	$[- 2{r}_{G}, 2{r}_{G}]$

### Measurements of performance

- *Mean value *
  The average value represents the mean of the best results obtained by an algorithm over various runs, and it can be determined as follows:
  10 $$\begin{aligned} Mean=\frac{1}{N} \sum _{i=1}^N {A_{i}}, \end{aligned}$$
  where $A_{i}$ denotes the best-obtained solution from $i^{th}$ run and *N* represents 20 independent runs.
- *Standard deviation (Std)*
  The standard deviation is determined to examine whether an algorithm can generate the same best value in multiple runs and to examine the repeatability of an algorithm’s outcomes, which can be calculated as follows:
  11 $$\begin{aligned} Std = \sqrt{\frac{1}{N} \sum _{i=1}^N (A_i -Mean)^2}, \end{aligned}$$
- *Best*
  The lowest of the results received from different runs:
  12 $$\begin{aligned} Best=Min_{1\le i\le N} {A_{i}^*}. \end{aligned}$$
- *Worst*
  The highest of the results received from different runs:
  13 $$\begin{aligned} Worst=Max_{1\le i\le N} {A_{i}^*}. \end{aligned}$$
- *Median*
  The ordered data’s middle value.
- *Wilcoxon rank-sum test*
  The Wilcoxon rank-sum test is used to determine whether two samples come from the same population.
- *Rank*
  Optimization algorithms base their performance ranking criteria on the t-test value.
- *t-test*
  A statistical test like a t-test is employed to estimate the significant differences between the proposed method with respect to other meta-heuristics. These are calculated as follows;
  14 $$\begin{aligned} t-value=\frac{Mean_{1}-Mean_{2}}{\sqrt{\frac{Std_{1}^2+Std_{2}^2}{N}}} , \end{aligned}$$
  where $Mean_{1}$, $Mean_{2}$, $Std_{1}$, and $Std_{2}$ be the mean and standard deviation for the two algorithms, respectively.

**Table 3:** 51 benchmark functions, U: Uni-modal, M: Multi-modal, S: Separable, N: Non-separable.

${\text {Name}}$	${\text {Function}}$	${\text {Characteristics}}$	${\text {Dimension}}$	${\text {Range}}$	${\text {f}}_{\text {optimal}}$
${\text {F1}}$	Sphere	US	30	[– 100, 100]	0
${\text {F2}}$	Schwefel’problem2.22	UN	30	[– 10, 10]	0
${\text {F3}}$	Schwefel’problem1.2	UN	30	[– 100, 100]	0
${\text {F4}}$	Schwefel’problem2.21	UN	30	[– 100, 100]	0
${\text {F5}}$	Rosen brock	UN	30	[– 30, 30]	0
${\text {F6}}$	Step	US	30	[– 100, 100]	0
${\text {F7}}$	Noise	US	30	[– 1.28, 1.28]	0
${\text {F8}}$	Generalized Schwefel’s problem	MS	30	[– 500, 500]	– 12569.5
${\text {F9}}$	Rastrigin	MS	30	[– 5.12, 5.12]	0
${\text {F10}}$	Ackley	MN	30	[– 32, 32]	0
${\text {F11}}$	Griewank	MN	30	[– 600, 600]	0
${\text {F12}}$	Generalized penalized function1	MN	30	[– 50, 50]	0
${\text {F13}}$	Generalized penalized function2	MN	30	[– 50, 50]	0
${\text {F14}}$	Shekel’s foxholes function	MS	2	[– 65, 65]	1
${\text {F15}}$	Kowalik’s function	MN	4	[– 5, 5]	0.00030
${\text {F16}}$	Six-hump camelback	MN	2	[– 5, 5]	– 1.0316
${\text {F17}}$	Branin	MS	2	[– 5, 5]	0.398
${\text {F18}}$	Goldstein-price function	MN	2	[– 2, 2]	3
${\text {F19}}$	Hartmann1	MN	3	[1, 3]	– 3.86
${\text {F20}}$	Hartmann2	MN	6	[0, 1]	– 3.32
${\text {F21}}$	Shekel1	MN	4	[0, 10]	– 10.1532
${\text {F22}}$	Shekel2	MN	4	[0, 10]	– 10.4028
${\text {F23}}$	Shekel3	MN	4	[0, 10]	– 10.5363
${\text {F24}}$	Stepint	US	5	[– 5.12, 5.12]	0
${\text {F25}}$	SumSquares	US	30	[– 10, 10]	0
${\text {F26}}$	Beale	UN	5	[– 4.5, 4.5]	0
${\text {F27}}$	Easom	UN	2	[– 100, 100]	– 1
${\text {F28}}$	Matyas	UN	2	[– 10, 10]	0
${\text {F29}}$	Colville	UN	4	[– 10, 10]	0
${\text {F30}}$	Trid6	UN	6	[$-D^2$,$D^2$]	– 360
${\text {F31}}$	Trid10	UN	10	[$-D^2$,$D^2$ ]	– 2600
${\text {F32}}$	Zakharov	UN	10	[– 5, 10]	0
${\text {F33}}$	Powell	UN	24	[– 4, 5]	0
${\text {F34}}$	Dixon– Price	UN	30	[– 10, 10]	0
${\text {F35}}$	Bohachevsky1	MS	2	[– 100, 100]	0
${\text {F36}}$	Booth	MS	2	[– 10, 10]	0
${\text {F37}}$	Michalewicz2	MS	2	[0,$\pi$]	– 1.8013
${\text {F38}}$	Michalewicz5	MS	5	[0,$\pi$]	– 4.6877
${\text {F39}}$	Michalewicz10	MS	10	[0,$\pi$]	– 9.6602
${\text {F40}}$	Schaffer	MN	2	[– 100, 100]	0
${\text {F41}}$	Bohachevshy2	MN	2	[– 100, 100]	0
${\text {F42}}$	Bohachevshy3	MN	2	[– 100, 100]	0
${\text {F43}}$	Shubert	MN	2	[– 100, 100]	– 25
${\text {F44}}$	Perm	MN	4	[$- D$,*D*]	0
${\text {F45}}$	PowerSum	MN	4	[0,*D*]	0
${\text {F46}}$	Langerman2	MN	2	[0, 10]	– 1.08
${\text {F47}}$	Langerman5	MN	5	[0, 10]	– 4.825
${\text {F48}}$	Langerman10	MN	10	[0, 10]	– 8.76
${\text {F49}}$	FletcherPowell2	MN	2	[$-\pi$,$\pi$]	0
${\text {F50}}$	FletcherPowell5	MN	5	[$-\pi$,$\pi$]	0
${\text {F51}}$	FletcherPowell10	MN	10	[$-\pi$,$\pi$]	0

**Table 4:** CEC 2019 test functions.

Functions	Dim	Range	$H_{min}$
${\text {CEC19-1}}$ = Storn’s chebyshev polynomial fitting problem	9	[– 8192, 8192]	1
${\text {CEC19-2}}$ = Inverse Hilbert matrix problem	16	[– 16384, 16384]	1
${\text {CEC19-3}}$ = Lennard– jones minimum energy cluster	18	[– 4, 4]	1
${\text {CEC19-4}}$ = Rastrigin’s function	10	[– 100, 100]	1
${\text {CEC19-5}}$ = Griewangk’s function	10	[– 100, 100]	1
${\text {CEC19-6}}$ = Weierstrass function	10	[– 100, 100]	1
${\text {CEC19-7}}$ = Modified schwefel’s function	10	[– 100, 100]	1
${\text {CEC19-8}}$ = Expanded schaffer’s F6 function	10	[– 100, 100]	1
${\text {CEC19-9}}$ = Happy cat function	10	[– 100, 100]	1
${\text {CEC19-10}}$ = Ackley function	10	[– 100, 100]	1

**Table 5:** Evaluation results of well-known & top-performing algorithms for the functions F1 to F15.

${\text {Functions}}$		${\text {PSO}}$	${\text {TSA}}$	${\text {SSA}}$	${\text {MVO}}$	${\text {GWO}}$	${\text {WOA}}$	${\text {GJO}}$	${\text {LSHADE}}$	${\text {CMAES}}$	${\text {LCA}}$
${\text {F1}}$	Mean	1.694E–05	3.954E–47	1.204E–08	3.086E–01	1.997E–59	6.61E–155	4.29E–113	2.005E–06	1.231E–24	0
	Std	1.926E–05	8.091E–47	2.692E–09	8.637E–02	3.320E–59	1.91E–154	1.06E–112	2.290E–06	1.120E–24	0
	Best	7.962E–07	6.342E–51	7.892E–09	1.741E–01	6.033E–6	7.18E–165	1.95E–117	1.429E–08	2.347E–25	0
	Worst	6.793E–05	2.649E–46	1.573E–08	4.288E–01	1.063E–58	6.56E–154	3.45E–112	7.216E–06	4.321E–24	0
	Median	1.106E–05	1.219E–48	1.273E–08	2.920E–01	3.049E–60	1.79E–161	2.46E–115	1.136E–06	8.201E–25	0
${\text {F2}}$	Mean	8.188E–03	6.762E–29	6.591E–01	5.155E–01	7.543E–35	5.75E–103	2.362E–66	9.163E–04	3.573E–12	0
	Std	4.409E–03	1.286E–28	8.061E–01	1.691E–01	5.818E–35	1.76E–102	3.454E–66	1.685E–03	1.408E–12	0
	Best	3.156E–03	3.278E–30	1.454E–02	3.085E–01	1.023E–35	2.50E–108	3.308E–67	5.388E–05	1.656E–12	0
	Worst	1.477E–02	4.296E–28	2.245E+00	7.696E–01	1.991E–34	5.72E–102	1.117E–65	7.189E–03	5.666E–12	0
	Median	7.437E–03	1.350E–29	3.143E–01	4.565E–01	6.116E–35	2.91E–107	8.758E–67	2.315E–04	3.678E–12	0
${\text {F3}}$	Mean	1.881E+01	1.413E–11	3.493E+02	4.653E+01	5.345E–14	2.465E+04	4.696E–39	3.474E+01	2.050E+00	0
	Std	8.849E+00	2.883E–11	2.755E+02	1.994E+01	1.613E–13	1.254E+04	1.398E–38	2.717E+01	1.389E+00	0
	Best	9.030E+00	1.878E–16	1.086E+02	2.501E+01	4.204E–20	4.527E+03	7.583E–46	3.729E+00	6.423E–01	0
	Worst	3.849E+01	8.084E–11	1.060E+03	8.514E+01	5.251E–13	4.534E+04	4.558E–38	9.065E+01	5.304E+00	0
	Median	1.646E+01	2.186E–14	2.304E+02	4.071E+01	1.079E–16	2.632E+04	5.334E–41	3.192E+01	1.764E+00	0
${\text {F4}}$	Mean	7.462E–01	5.400E–03	8.570E+00	9.789E–01	8.888E–15	4.222E+01	1.115E–32	8.418E+00	7.135E–10	0
	Std	1.685E–01	1.156E–02	3.952E+00	4.094E–01	6.057E–15	3.313E+01	3.029E–32	2.191E+00	2.074E–10	0
	Best	4.673E–01	4.023E–05	1.995E+00	4.774E–01	9.666E–16	7.332E–01	2.987E–35	5.458E+00	4.974E–10	0
	Worst	1.119E+00	3.885E–02	1.361E+01	1.850E+00	1.847E–14	8.772E+01	9.945E–32	1.298E+01	1.357E–09	0
	Median	7.357E–01	1.420E–03	8.594E+00	9.772E–01	1.095E–14	4.356E+01	6.003E–34	8.071E+00	6.338E–10	0
${\text {F5}}$	Mean	1.567E+02	2.815E+01	4.130E+02	7.026E+02	2.673E+01	2.750E+01	2.773E+01	4.638E+01	2.705E+00	0
	Std	1.342E+02	8.143E–01	7.855E+02	9.315E+02	6.005E–01	7.525E–01	7.617E–01	2.930E+01	6.222E–01	0
	Best	2.972E+01	2.642E+01	2.514E+01	3.361E+01	2.617E+01	2.664E+01	2.624E+01	3.970E+00	1.777E+00	0
	Worst	4.328E+02	2.886E+01	2.671E+03	2.481E+03	2.793E+01	2.875E+01	2.887E+01	8.412E+01	3.812E+00	0
	Median	9.772E+01	2.833E+01	1.404E+02	1.578E+02	2.667E+01	2.715E+01	2.80E+01	2.916E+01	2.748E+00	0
${\text {F6}}$	Mean	1.770E–05	3.655E+00	1.513E–08	3.024E–01	8.147E–01	1.735E–01	2.312E+00	1.672E–06	1.082E–24	0
	Std	1.063E–05	4.294E–01	6.384E–09	8.260E–02	3.183E–01	2.382E–01	4.422E–01	4.948E–06	8.075E–25	0
	Best	3.187E–06	2.576E+00	1.008E–08	1.885E–01	2.516E–01	1.111E–02	1.749E+00	2.501E–08	1.832E–25	0
	Worst	3.810E–05	4.050E+00	3.033E–08	5.125E–01	1.483E+00	7.978E–01	3.013E+00	2.243E–05	2.513E–24	0
	Median	1.582E–05	3.814E+00	1.254E–08	3.032E–01	7.491E–01	4.599E–02	2.246E+00	2.701E–07	8.051E–25	0

${\text {F7}}$	Mean	8.297E–02	5.990E–03	9.157E–02	2.281E–02	8.363E–04	9.469E–04	2.573E–04	5.008E–02	3.668E–03	2.969E–05
	Std	1.844E–02	1.790E–03	3.812E–02	6.110E–03	3.756E–04	8.758E–04	1.034E–04	2.134E–02	1.629E–03	1.651E–05
	Best	4.813E–02	3.650E–03	3.571E–02	1.466E–02	1.959E–04	1.163E–04	1.081E–04	2.631E–02	7.526E–04	1.534E–05
	Worst	1.055E–01	9.810E–03	1.628E–01	3.041E–02	1.362E–03	2.093E–03	4.528E–04	1.054E–01	6.576E–03	6.860E–05
	Median	8.990E–02	5.750E–03	9.604E–02	2.552E–02	7.988E–04	3.755E–04	2.354E–04	4.582E–02	3.698E–03	2.213E–05
${\text {F8}}$	Mean	–6.67E+03	–6.45E+03	–4.75E+04	–8.04E+03	–6.23E+03	–1.19E+04	–4.38E+03	–1.23E+04	–3.3E+306	–1.26E+04
	Std	9.59E+02	6.85E+02	8.52E+03	3.03E+02	4.75E+02	9.02E+02	1.08E+03	1.68E+02	*NA*	1.87E–12
	Best	–8.17E+03	–7.31E+03	–6.49E+04	–8.69E+03	–7.19E+03	–1.26E+04	–6.49E+03	–1.26E+04	–2.9E+307	–1.26E+04
	Worst	–5.07E+03	–5.27E+03	–3.50E+04	–7.54E+03	–5.69E+03	–9.79E+03	–2.70E+03	–1.20E+04	–1.3E+296	–1.26E+04
	Median	–6.68E+03	–6.73E+03	–4.63E+04	–8.07E+03	–6.08E+03	–1.22E+04	–4.32E+03	–1.22E+04	–4.7E+303	–1.26E+04
${\text {F9}}$	Mean	49.85266	164.25398	52.13577	105.04556	0.45353	0	0	0.049825	157.6098	0
	Std	9.156932	35.025847	10.46209	33.65920	1.395935	0	0	0.222481	7.324355	0
	Best	33.8776	116.1056	36.8135	51.9801	0	0	0	1.275E–06	141.9516	0
	Worst	64.6898	211.1062	73.6268	164.3799	4.5353	0	0	0.995044	165.2087	0
	Median	49.3953	171.94725	49.74785	98.6025	0	0	0	4.593E–05	159.2086	0
${\text {F10}}$	Mean	9.969E–02	1.855E+00	1.818E+00	1.271E+00	1.652E–14	3.730E–15	4.440E–15	2.450E+00	2.892E–13	4.441E–16
	Std	2.847E–01	1.572E+00	1.026E+00	6.649E–01	2.915E–15	2.187E–15	2.428E–30	5.409E–01	1.621E–13	1.012E–31
	Best	1.837E–03	1.509E–14	3.002E–05	2.279E–01	1.509E–14	8.881E–16	4.440E–15	1.501E+00	1.354E–13	4.441E–16
	Worst	9.318E–01	3.546E+00	2.957E+00	2.383E+00	2.220E–14	7.993E–15	4.440E–15	3.518E+00	7.038E–13	4.441E–16
	Median	4.268E–03	2.762E+00	2.117E+00	1.276E+00	1.509E–14	4.440E–15	4.440E–15	2.495E+00	2.491E–13	4.441E–16
${\text {F11}}$	Mean	0.0167412	0.016606	0.0132815	0.593154	0.0009793	0	0	0.015958	0	0
	Std	0.0095002	0.017481	0.0097684	0.104295	0.0030144	0	0	0.021317	0	0
	Best	1.229E–07	0	5.5395E–08	0.41554	0	0	0	5.590E–08	0	0
	Worst	0.029553	0.061033	0.031898	0.73028	0.0097937	0	0	0.095192	0	0
	Median	0.0172275	0.015464	0.013547	0.61796	0	0	0	0.012328	0	0
${\text {F12}}$	Mean	4.204E–07	7.471E+00	5.298E+00	1.468E+00	4.481E–02	7.131E–03	2.197E–01	5.262E–01	8.396E–24	1.570E–32
	Std	2.873E–07	2.605E+00	2.743E+00	8.326E–01	2.331E–02	5.741E–03	7.453E–02	1.281E+00	1.968E–23	2.808E–48
	Best	4.173E–08	3.765E+00	2.353E+00	4.619E–01	6.561E–03	1.968E–03	1.303E–01	4.497E–08	3.518E–25	1.570E–32
	Worst	9.178E–07	1.200E+01	1.065E+01	3.018E+00	7.885E–02	1.977E–02	3.619E–01	5.678E+00	9.136E–23	1.570E–32
	Median	4.548E–07	7.271E+00	4.935E+00	1.511E+00	3.803E–02	5.804E–03	1.942E–01	1.036E–01	4.045E–24	1.570E–32
${\text {F13}}$	Mean	2.204E–03	3.239E+00	1.850E–02	7.295E–02	4.655E–01	2.446E–01	1.545E+00	1.009E–01	6.238E–23	1.349E–32
	Std	4.511E–03	7.530E–01	3.047E–02	3.941E–02	1.863E–01	1.437E–01	1.692E–01	3.550E–01	7.751E–23	5.616E–48
	Best	2.490E–07	1.880E+00	1.256E–09	2.411E–02	1.128E–01	2.209E–02	1.380E+00	6.150E–08	4.119E–24	1.349E–32
	Worst	1.100E–02	4.506E+00	9.814E–02	1.573E–01	7.413E–01	5.548E–01	1.901E+00	1.597E+00	2.477E–22	1.349E–32
	Median	4.661E–06	3.030E+00	5.493E–03	6.252E–02	5.004E–01	2.251E–01	1.495E+00	1.109E–02	3.125E–23	1.349E–32
${\text {F14}}$	Mean	4.53782	7.62641	1.0974	0.998	4.3167	1.99005	7.82613	0.998003	4.856805	0.998
	Std	3.24061	5.27654	0.30594	0	4.8206	1.01782	4.98902	2.278E–16	2.939305	0
	Best	0.998	0.998	0.998	0.998	0.998	0.998	0.998	0.998003	0.99801	0.998
	Worst	10.7632	15.5038	1.992	0.998	12.6705	2.9821	12.6705	0.998003	10.7632	0.998
	Median	2.9821	8.346	0.998	0.998	0.998	1.9900	10.7632	0.998003	4.0102	0.998
${\text {F15}}$	Mean	8.699E–04	4.604E–03	1.005E–03	4.715E–03	4.501E–03	8.040E–04	1.579E–03	1.356E–03	2.491E–03	3.109E–04
	Std	2.897E–04	8.097E–03	2.593E–04	8.030E–03	8.144E–03	5.107E–04	3.806E–03	4.478E–03	1.462E–03	3.534E–06
	Best	3.145E–04	3.079E–04	5.634E–04	3.256E–04	3.074E–04	3.088E–04	3.075E–04	3.074E–04	1.135E–03	3.087E–04
	Worst	1.085E–03	2.036E–02	1.479E–03	2.036E–02	2.036E–02	1.503E–03	1.270E–02	2.036E–02	5.810E–03	3.185E–04
	Median	9.818E–04	4.620E–04	1.067E–03	7.598E–04	3.085E–04	5.751E–04	3.121E–04	3.074E–04	1.782E–03	3.089E–04

**Table 6:** Evaluation results of well-known & top-performing algorithms for the functions F16 to F30.

${\text {Functions}}$		${\text {PSO}}$	${\text {TSA}}$	${\text {SSA}}$	${\text {MVO}}$	${\text {GWO}}$	${\text {WOA}}$	${\text {GJO}}$	${\text {LSHADE}}$	${\text {CMAES}}$	${\text {LCA}}$
${\text {F16}}$	Mean	$-1.0316$	$-1.0316$	$-1.0316$	$-1.0316$	$-1.0316$	$-1.0316$	$-1.0316$	$-1.0316$	$-1.0316$	$-1.0316$
	Std	$4.5E-16$	$4.5E-16$	$4.5E-16$	$4.5E-16$	$4.5E-16$	$4.5E-16$	$4.5E-16$	$4.5E-16$	$4.5E-16$	$4.5E-16$
	Best	$-1.0316$	$-1.0316$	$-1.0316$	$-1.0316$	$-1.0316$	$-1.0316$	$-1.0316$	$-1.0316$	$-1.0316$	$-1.0316$
	Worst	$-1.0316$	$-1.0316$	$-1.0316$	$-1.0316$	$-1.0316$	$-1.0316$	$-1.0316$	$-1.0316$	$-1.0316$	$-1.0316$
	Median	$-1.0316$	$-1.0316$	$-1.0316$	$-1.0316$	$-1.0316$	$-1.0316$	$-1.0316$	$-1.0316$	$-1.0316$	$-1.0316$
${\text {F17}}$	Mean	0.39789	0.397954	0.39789	0.39789	0.397957	0.39789	0.39796	0.397887	0.39789	0.397931
	Std	1.139E–16	9.735E–05	1.139E–16	1.139E–16	0.000206	1.139E–16	0.000195	1.139E–16	1.139E–16	3.596E–05
	Best	0.39789	0.39789	0.39789	0.39789	0.39789	0.39789	0.39789	0.397887	0.39789	0.39789
	Worst	0.39789	0.3982	0.39789	0.39789	0.39856	0.39789	0.39853	0.397887	0.39789	0.39799
	Median	0.39789	0.39792	0.39789	0.39789	0.39789	0.39789	0.39789	0.397887	0.39789	0.39793
${\text {F18}}$	Mean	3	3	3	3	3	3	3	3	3.000000001	3
	Std	0	0	0	0	0	0	0	0	$1.522E-09$	0
	Best	3	3	3	3	3	3	3	3	3	3
	Worst	3	3	3	3	3	3	3	3	3.000000003	3
	Median	3	3	3	3	3	3	3	3	3	3
${\text {F19}}$	Mean	–3.8628	–3.8619	–3.8628	–3.8628	–3.8612	–3.8593	–3.8572	–3.862782	–3.8628	–3.8581
	Std	1.3E–15	0.00237	1.3E–15	1.3E–15	0.003232	0.003089	0.000365	9.112E–16	1.3E–15	0.00417
	Best	–3.8628	–3.8628	–3.8628	–3.8628	–3.8628	–3.8627	–3.8628	–3.862782	–3.8628	–3.8617
	Worst	–3.8628	–3.8550	–3.8628	–3.8628	–3.8549	–3.8549	–3.8549	–3.862782	–3.8628	–3.8619
	Median	–3.8628	–3.8627	–3.8628	–3.8628	–3.8628	–3.8595	–3.8549	–3.862782	–3.8628	–3.8617
${\text {F20}}$	Mean	–3.26255	–3.22041	–3.2331	–3.2623	–3.24607	–3.17022	–3.21823	–3.3219	–3.292275	–2.7719
	Std	0.060994	0.076133	0.060158	0.061251	0.09606	0.263940	0.078602	4.556E–16	0.052822	0.18520
	Best	–3.322	–3.3209	–3.322	–3.322	–3.322	–3.3219	–3.322	–3.3219	–3.322	–2.9438
	Worst	–3.2031	–3.0861	–3.1804	–3.202	–3.104	–2.4317	–3.0867	–3.3219	–3.2031	–2.5042
	Median	–3.26255	–3.20185	–3.20185	–3.2625	–3.322	–3.2517	–3.19945	–3.3219	–3.322	–2.821
${\text {F21}}$	Mean	–5.64936	–5.31385	–8.1441	–8.1185	–9.64751	–9.63652	–8.12049	–10.153199	$-10.1532$	$-10.1532$
	Std	3.194666	3.251232	3.21216	2.55663	1.555147	1.56683	2.549866739	1.822E–15	0	0
	Best	$-10.1532$	–10.349	$-10.1532$	$-10.1532$	$-10.1532$	$-10.1532$	–10.1518	–10.1531	$-10.1532$	$-10.1532$
	Worst	–2.6829	–2.6246	–2.6305	–5.0552	–5.1002	–5.0552	–5.0551	–10.1531	$-10.1532$	$-10.1532$
	Median	-5.1008	–3.8311	–10.1532	–10.1531	–10.1532	–10.14965	–10.14685	–10.1531	$-10.1532$	$-10.1532$

${\text {F22}}$	Mean	–7.29187	–7.23034	–8.58438	–7.75759	–10.4029	–8.56993	–9.86786	–10.4029	–10.4029	–10.4029
	Std	3.30111	3.760581	2.91792	2.71400	2.190E–04	2.67942	1.634849	5.467E–15	3.6E–15	3.6E–15
	*Best*	–10.4029	–10.289	–10.4029	–10.4029	–10.4028	–10.4029	–10.4029	–10.4029	–10.4029	–10.4029
	Worst	–2.7519	–2.7241	–2.7659	–5.0876	–10.4022	–3.7243	–5.0875	–10.4029	–10.4029	–10.4029
	Median	–7.76585	–10.1615	–10.4029	–7.7658	–10.4026	–10.3071	–10.3994	–10.4029	–10.4029	–10.4029
${\text {F23}}$	Mean	–8.91403	–6.44063	–9.23379	–7.8418	–9.72462	–10.5348	–9.99744	$-10.5364$	$-10.5364$	$-10.5364$
	Std	2.54259	3.36187	2.72468	2.76452	2.497545	0.001619	1.649480	5.467E–15	5.467E–15	5.467E–15
	Best	$-10.5364$	–10.4249	–10.5364	–10.5364	–10.5363	–10.5361	$-10.5364$	$-10.5364$	–10.5364	–10.5364
	Worst	–5.1285	–2.4201	–2.8711	–5.1284	–2.4217	–10.5304	–5.1743	–10.5364	–10.5364	–10.5364
	Median	–10.5364	–5.12535	–10.5364	–7.85585	–10.5361	–10.5353	–10.5364	–10.5364	–10.5364	–10.5364
${\text {F24}}$	Mean	–17076.3	0.3	–4830.9	$-5$	$-5$	$-5$	$-5$	$-5$	$-5$	$-5$
	Std	176.514	2.59756	78.5908	0	0	0	0	0	0	0
	Best	–17384	$-5$	–4941	$-5$	$-5$	$-5$	$-5$	$-5$	$-5$	$-5$
	Worst	–16810	6	–4703	$-5$	$-5$	$-5$	$-5$	$-5$	$-5$	$-5$
	Median	–17128.5	0	–4852	$-5$	$-5$	$-5$	$-5$	$-5$	$-5$	$-5$
${\text {F25}}$	Mean	5.526E–04	1.390E–48	1.924E–01	2.435E–01	2.160E–60	2.429E–151	3.277E–112	2.075E–07	1.594E–25	0
	Std	1.208E–03	2.205E–48	1.897E–01	2.483E–01	2.792E–60	7.358E–151	7.534E–112	2.625E–07	1.370E–25	0
	Best	2.653E–05	4.494E–51	4.543E–03	6.264E–02	9.171E–63	1.998E–177	1.263E–117	3.590E–09	2.930E–26	0
	Worst	4.066E–03	7.146E–48	7.266E–01	7.317E–01	9.493E–60	2.394E–150	2.381E–111	8.620E–07	6.519E–25	0
	Median	9.811E–05	2.841E–49	1.634E–01	1.577E–01	7.395E–61	1.124E–158	5.475E–115	8.210E–08	1.315E–25	0
${\text {F26}}$	Mean	0	2.180E–01	1.082E–01	7.621E–02	7.621E–02	1.400E–10	3.586E–07	0	2.718E–05	2.591E–04
	Std	0	3.428E–01	2.222E–01	2.345E–01	2.345E–01	4.026E–10	3.586E–07	0	6.328E–05	8.348E–05
	Best	0	1.169E–07	4.828E–18	8.310E–10	1.834E–09	3.322E–14	1.056E–08	0	5.314E–08	2.271E–04
	Worst	0	7.620E–01	5.437E–01	7.620E–01	7.629E–01	1.316E–09	1.148E–06	0	0.0002893	5.015E–04
	Median	0	7.758E–07	4.509E–16	3.101E–08	4.199E–08	1.027E–12	2.100E–07	0	0.0000128	2.271E–04
${\text {F27}}$	Mean	–1	–0.7499	–1	–0.8999	–1	–1	–1	–1	–1	–1
	Std	0	0	0.4442	0	0.3077	0	0	0	0	0
	Best	–1	–1	–1	–1	–1	–1	–1	–1	–1	–1
	Worst	–1	0	–1	0	–1	–1	–1	–1	–1	–1
	Median	–1	–0.9999	–1	–0.9999	–1	–1	–1	–1	–1	–1
${\text {F28}}$	Mean	8.063E–52	2.833E–155	6.168E–16	7.059E–09	1.896E–218	0	0	1.840E–127	3.050E–306	0
	Std	1.595E–51	0	5.759E–16	4.452E–09	0	0	0	8.212E–127	0	0
	Best	1.002E–54	1.635E–164	7.169E–17	2.366E–10	1.199E–237	0	0	2.084E–147	5.566E–308	0
	Worst	5.089E–51	2.395E–154	1.943E–15	1.361E–08	1.334E–217	0	0	3.673E–126	1.150E–305	0
	Median	5.083E–53	7.151E–158	3.846E–16	7.857E–09	3.903E–223	0	0	6.004E–135	2.598E–307	0
${\text {F29}}$	Mean	1.579E–02	1.549E+00	2.714E–01	7.401E–03	1.002E+00	1.036E+00	1.057E+00	0	1.900E–10	0
	Std	1.094E–02	1.944E+00	7.900E–01	9.976E–03	6.885E–01	1.180E+00	6.186E–01	0	4.997E–10	0
	Best	1.110E–05	3.521E–03	2.153E–04	2.035E–04	9.909E–04	7.515E–03	1.154E–01	0	2.271E–21	0
	Worst	3.096E–02	6.902E+00	2.581E+00	2.913E–02	1.608E+00	3.450E+00	1.568E+00	0	1.936E–09	0
	Median	1.645E–02	1.352E+00	1.283E–02	1.582E–03	1.424E+00	7.047E–01	1.328E+00	0	8.872E–12	0
${\text {F30}}$	Mean	–5.602E+06	–316.7	–4.181E+07	$-360$	–324	–345.6	–345.6	$-360$	–2.00E+306	$-360$
	Std	3.186E+04	36.274	1.397E+06	0	36.93	29.548	29.548	0	*NA*	0
	Best	–5.634E+06	$-360$	–4.373E+07	$-360$	$-360$	$-360$	$-360$	$-360$	–4.01E+307	$-360$
	Worst	–5.536E+06	–287	–3.880E+07	$-360$	–288	–288	–288	$-360$	–2.53E+150	$-360$
	Median	–5.614E+06	–288	–4.176E+07	$-360$	–324	$-360$	$-360$	$-360$	–5.35E+226	$-360$

**Table 7:** Evaluation results of well-known & top-performing algorithms for the functions F31 to F51.

${\text {Functions}}$		${\text {PSO}}$	${\text {TSA}}$	${\text {SSA}}$	${\text {MVO}}$	${\text {GWO}}$	${\text {WOA}}$	${\text {GJO}}$	${\text {LSHADE}}$	${\text {CMAES}}$	${\text {LCA}}$
${\text {F31}}$	Mean	–5.633E+06	–2519.79	–3.263E+08	–2540	–2540	–2560	–2540	–2520	–3.03E+301	$-2600$
	Std	4.531E+04	100.56	7.148E+06	94.032	94.032	82.078	94.032	100.52	NA	0.6545
	Best	–5.703E+06	$-2600$	–3.379E+08	$-2600$	$-2600$	$-2600$	$-2600$	$-2600$	–5.94E+302	$-2600$
	Worst	–5.539E+06	–2398.99	–3.172E+08	–2400	–2400	–2400	–2400	–2400	–1.45E+192	$-2600$
	Median	–5.629E+06	–2599.49	–3.281E+08	$-2600$	$-2600$	$-2600$	$-2600$	$-2600$	–7.50E+255	$-2600$
${\text {F32}}$	Mean	9.808E–21	1.827E–84	5.047E–11	4.048E–04	6.133E–118	5.475E–158	4.487E–219	3.296E–36	0.136153	0
	Std	1.354E–20	5.552E–84	2.616E–11	2.618E–04	1.565E–117	0	0	1.160E–35	0.091605	0
	Best	1.217E–22	1.933E–91	1.897E–11	1.019E–04	7.510E–121	1.989E–169	4.273E–230	9.272E–44	0.037223	0
	Worst	4.267E–20	1.806E–83	1.034E–10	8.838E–04	5.118E–117	5.474E–157	4.484E–218	5.190E–35	0.40012	0
	Median	1.645E–21	8.026E–88	4.956E–11	3.425E–04	3.982E–120	5.042E–166	9.533E–224	4.418E–38	0.120975	0
${\text {F33}}$	Mean	3.929E–02	4.603E–05	8.692E–01	3.761E–01	3.740E–06	6.371E–06	4.489E–09	4.290E–04	1.852E–01	0
	Std	3.131E–02	4.765E–05	8.597E–01	1.572E–01	4.180E–06	1.247E–05	1.272E–08	3.710E–04	1.210E–01	0
	Best	1.051E–02	1.908E–06	4.569E–01	2.139E–01	8.266E–07	2.406E–15	5.559E–14	1.802E–05	5.336E–02	0
	Worst	1.162E–01	1.393E–04	3.358E+00	7.637E–01	1.308E–05	3.488E–05	4.167E–08	1.428E–03	4.742E–01	0
	Median	2.947E–02	2.109E–05	5.812E–01	3.307E–01	1.917E–06	3.571E–07	7.753E–12	2.959E–04	1.324E–01	0
${\text {F34}}$	Mean	1.912E–29	6.000E–01	2.838E–14	6.454E–06	1.393E–09	8.329E–07	3.718E–06	0	5.663E–05	0
	Std	3.915E–29	5.026E–01	2.892E–14	9.092E–06	8.038E–10	1.509E–06	5.835E–06	0	7.594E–05	0
	Best	0	7.450E–09	8.906E–16	5.052E–08	3.183E–10	7.660E–12	5.679E–10	0	2.384E–06	0
	Worst	9.555E–29	1.0003	8.228E–14	2.313E–05	2.638E–09	3.770E–06	1.469E–05	0	3.362E–04	0
	Median	0	1	1.624E–14	8.876E–07	1.104E–09	7.099E–08	1.649E–08	0	3.073E–05	0
${\text {F35}}$	Mean	0	0	5.480E–12	1.714E–04	0	0	0	0	0	0
	Std	0	0	4.281E–12	1.351E–04	0	0	0	0	0	0
	Best	0	0	2.957E–13	1.532E–05	0	0	0	0	0	0
	Worst	0	0	1.223E–11	4.835E–04	0	0	0	0	0	0
	Median	0	0	4.154E–12	1.517E–04	0	0	0	0	0	0
${\text {F36}}$	Mean	0	1.800E–01	1.504E–14	3.950E–07	8.052E–08	2.051E–04	7.572E–07	0	0	0
	Std	0	5.540E–01	1.587E–14	3.354E–07	5.999E–08	1.669E–04	8.078E–07	0	0	0
	Best	0	4.319E–07	4.824E–16	6.200E–08	1.487E–08	7.203E–06	1.751E–08	0	0	0
	Worst	0	1.800E+00	4.752E–14	1.229E–06	2.215E–07	5.154E–04	2.446E–06	0	0	0
	Median	0	2.881E–06	9.595E–15	3.015E–07	7.783E–08	1.822E–04	4.921E–07	0	0	0
${\text {F37}}$	Mean	$-1.9093$	$-1.80126$	$-1.89264$	$-1.8013$	$-1.8013$	$-1.8013$	$-1.8013$	$-1.8013$	$-1.89296$	$-1.8013$
	Std	0.09078	5.026E–05	0.09389	2.200E–16	2.200E–16	2.200E–16	2.200E–16	2.200E–16	0.060191	2.200E–16
	Best	$-1.988$	$-1.8013$	$-1.988$	$-1.8013$	$-1.8013$	$-1.8013$	$-1.8013$	$-1.8013$	$-1.9883$	$-1.8013$
	Worst	$-1.8013$	$-1.8012$	$-1.8013$	$-1.8013$	$-1.8013$	$-1.8013$	$-1.8013$	$-1.8013$	$-1.8049$	$-1.8013$
	Median	$-1.9679$	$-1.8013$	$-1.8846$	$-1.8013$	$-1.8013$	$-1.8013$	$-1.8013$	$-1.8013$	$-1.91565$	$-1.8013$
${\text {F38}}$	Mean	–4.672E+00	–4.072E+00	–4.016E+00	–4.242E+00	–4.534E+00	–3.814E+00	–3.897E+00	–4.687658	–4.50042	–3.257E+00
	Std	4.752E–02	6.054E–01	6.354E–01	3.449E–01	5.962E–02	5.724E–01	2.747E–01	9.112E–16	0.179110	1.260E–01
	Best	–4.704E+00	–4.589E+00	–4.645E+00	–4.687E+00	–4.645E+00	–4.665E+00	–4.452E+00	–4.687658	–4.6877	–3.426E+00
	Worst	–4.537E+00	–2.747E+00	–2.600E+00	–3.686E+00	–4.495E+00	–2.885E+00	–3.618E+00	–4.687658	–4.136	–3.056E+00
	Median	–4.686E+00	–4.407E+00	–4.101E+00	–4.331E+00	–4.494E+00	–3.615E+00	–3.844E+00	–4.687658	–4.56845	–3.227E+00
${\text {F39}}$	Mean	–8.869E+00	–6.493E+00	–6.496E+00	–7.708E+00	–7.910E+00	–5.962E+00	–6.184E+00	–9.660151	–6.92552	–5.661E+00
	Std	5.371E–01	5.907E–01	7.129E–01	7.124E–01	1.216E+00	6.052E–01	1.035E+00	3.645E–15	0.631307	4.317E–01
	Best	–9.518E+00	–7.531E+00	–8.136E+00	–8.865E+00	–9.503E+00	–7.046E+00	–7.723E+00	–9.660151	–8.6191	–6.336E+00
	Worst	–7.990E+00	–5.855E+00	–5.517E+00	–6.589E+00	–5.489E+00	–5.014E+00	–4.310E+00	–9.660151	–5.9688	–5.227E+00
	Median	–8.796E+00	–6.261E+00	–6.361E+00	–7.905E+00	–8.085E+00	–5.854E+00	–6.285E+00	–9.660151	–6.8364	–5.449E+00
${\text {F40}}$	Mean	0	0	2.664E–16	8.298E–09	0	0	0	0	1.703E–05	0
	Std	0	0	3.022E–16	9.054E–09	0	0	0	0	1.401E–05	0
	Best	0	0	0	1.338E–09	0	0	0	0	2.181E–06	0
	Worst	0	0	8.881E–16	2.991E–08	0	0	0	0	5.771E–05	0
	Median	0	0	2.220E–16	3.508E–09	0	0	0	0	1.385E–05	0

${\text {F41}}$	Mean	0	0	1.007E–11	1.187E–04	0	0	0	0	5.112E–03	0
	Std	0	0	1.197E–11	1.229E–04	0	0	0	0	5.660E–03	0
	Best	0	0	6.614E–13	4.069E–06	0	0	0	0	2.104E–04	0
	Worst	0	0	4.002E–11	3.553E–04	0	0	0	0	2.137E–02	0
	Median	0	0	5.375E–12	8.998E–05	0	0	0	0	3.596E–03	0
${\text {F42}}$	Mean	0	4.525E–02	4.931E–12	3.013E–05	0	2.275E–16	0	0	1.285E–03	0
	Std	0	9.286E–02	4.915E–12	2.564E–05	0	1.913E–16	0	0	8.107E–04	0
	Best	0	0	2.765E–13	1.852E–07	0	0	0	0	5.142E–05	0
	Worst	0	2.262E–01	1.326E–11	8.532E–05	0	4.996E–16	0	0	2.587E–03	0
	Median	0	0	2.097E–12	2.857E–05	0	2.220E–16	0	0	1.346E–03	0
${\text {F43}}$	Mean	$-25$	$-24.99872$	$-25$	$-25$	$-24.9985$	$-25$	$-24.99759$	$-24.9999$	$-24.9996$	$-25$
	Std	0	0.000996	0	0	0.002237	0	0.003963	7.290E–15	0.000256	0
	Best	$-25$	$-24.9998$	$-25$	$-25$	$-25$	$-25$	$-25$	$-24.9999$	$-24.9999$	$-25$
	Worst	$-25$	$-24.9968$	$-25$	$-25$	$-24.9925$	$-25$	$-24.9879$	$-24.9999$	$-24.9992$	$-25$
	Median	$-25$	$-24.999$	$-25$	$-25$	$-24.9995$	$-25$	$-24.9993$	$-24.9999$	$-24.9997$	$-25$
${\text {F44}}$	Mean	0	0	1.337E–14	0	0	0	0	0	4.501E–06	0
	Std	0	0	1.698E–14	0	0	0	0	0	1.199E–05	0
	Best	0	0	7.658E–17	0	0	0	0	0	1.001E–09	0
	Worst	0	0	6.020E–14	0	0	0	0	0	3.954E–05	0
	Median	0	0	8.522E–15	0	0	0	0	0	1.237E–07	0
${\text {F45}}$	Mean	0	3.409E–06	1.262E–14	1.590E–07	1.824E–06	4.402E–12	2.724E–06	0	1.252E–07	0
	Std	0	6.038E–06	2.422E–14	1.688E–07	1.695E–06	1.029E–11	4.129E–06	0	2.670E–07	0
	Best	0	3.326E–09	1.579E–18	5.559E–09	2.502E–08	1.034E–15	5.573E–08	0	1.678E–11	0
	Worst	0	2.024E–05	8.119E–14	4.924E–07	4.873E–06	3.413E–11	1.142E–05	0	9.250E–07	0
	Median	0	5.062E–07	3.225E–15	6.255E–08	1.515E–06	7.322E–14	5.841E–07	0	2.886E–08	0
${\text {F46}}$	Mean	$-1.0341$	$-0.985586$	$-1.0341$	$-1.0341$	$-1.0341$	$-1.0341$	$-0.959692$	$-1.0341$	$-1.0341$	$-1.0341$
	Std	0	0.099343	0	0	0	0	0.152476	0	0	0
	Best	$-1.034$	$-1.034$	$-1.034$	$-1.034$	$-1.034$	$-1.034$	$-1.034$	$-1.034$	$-1.034$	$-1.034$
	Worst	$-1.034$	–0.79193	$-1.034$	$-1.034$	$-1.034$	$-1.034$	$-1.034$	$-1.034$	$-1.034$	$-1.034$
	Median	$-1.034$	$-1.034$	$-1.034$	$-1.034$	$-1.034$	$-1.034$	$-1.034$	$-1.034$	$-1.034$	$-1.034$
${\text {F47}}$	Mean	–4.825E+00	–4.011E+00	–4.053E+00	–4.535E+00	–4.439E+00	–4.103E+00	–4.299E+00	–4.825E+00	–4.824E+00	–4.62031
	Std	9.112E–16	7.259E–01	5.938E–01	4.537E–01	4.850E–01	5.964E–01	6.390E–01	9.112E–16	1.822E–15	0.2888552
	Best	–4.825E+00	–4.822E+00	–4.825E+00	–4.825E+00	–4.825E+00	–4.825E+00	–4.825E+00	–4.825E+00	–4.824E+00	–4.6849
	Worst	–4.825E+00	–2.895E+00	–2.895E+00	–3.860E+00	–3.860E+00	–2.895E+00	–2.916E+00	–4.825E+00	–4.824E+00	–3.3931
	Median	–4.825E+00	–3.858E+00	–3.860E+00	–4.825E+00	–4.825E+00	–3.863E+00	–4.599E+00	–4.825E+00	–4.824E+00	–4.6849
${\text {F48}}$	Mean	–8.760E+00	–6.560E+00	–5.349E+00	–6.312E+00	–7.449E+00	–5.369E+00	–5.602E+00	–8.760E+00	–8.444E+00	–5.838515
	Std	0	5.729E–01	1.068E+00	5.138E–01	7.223E–01	6.663E–01	8.037E–01	0	3.282E–01	0.2436643
	Best	–8.760E+00	–7.320E+00	–6.666E+00	–7.027E+00	–8.760E+00	–6.591E+00	–7.007E+00	–8.760E+00	–8.753E+00	–5.893
	Worst	–8.760E+00	–5.316E+00	–3.504E+00	–5.724E+00	–7.007E+00	–4.518E+00	–4.385E+00	–8.760E+00	–7.691E+00	–4.8033
	Median	–8.760E+00	–6.571E+00	–5.662E+00	–6.153E+00	–7.008E+00	–5.202E+00	–5.705E+00	–8.760E+00	–8.563E+00	–5.893
${\text {F49}}$	Mean	6.563E–29	1.570E–07	5.499E–17	3.152E–09	7.079E–08	3.616E–14	5.792E–07	0	1.696E–07	0
	Std	1.351E–28	2.465E–07	7.682E–17	4.124E–09	9.338E–08	8.253E–14	9.751E–07	0	3.635E–07	0
	Best	0	9.363E–10	7.884E–19	1.828E–11	1.257E–11	3.156E–17	2.556E–09	0	8.054E–13	0
	Worst	4.543E–28	8.583E–07	2.502E–16	1.135E–08	2.849E–07	2.731E–13	3.256E–06	0	1.195E–06	0
	Median	2.524E–29	9.174E–08	1.496E–17	4.614E–10	3.056E–08	3.527E–15	2.282E–07	0	2.618E–08	0
${\text {F50}}$	Mean	0	2.785E–07	1.900E–16	1.062E–07	3.451E–06	7.273E–14	1.530E–06	0	5.528E–07	0
	Std	0	5.418E–07	2.729E–16	3.083E–07	9.023E–06	2.034E–13	2.063E–06	0	1.443E–06	0
	Best	0	1.219E–10	8.448E–20	3.060E–10	1.050E–12	1.059E–16	1.250E–10	0	3.299E–09	0
	Worst	0	1.845E–06	7.341E–16	1.007E–06	2.950E–05	6.670E–13	6.349E–06	0	4.759E–06	0
	Median	0	1.315E–07	5.807E–17	6.862E–09	3.732E–08	3.449E–15	8.437E–07	0	1.768E–08	0
${\text {F51}}$	Mean	4.543E–29	1.253E–07	5.579E–16	7.685E–08	7.820E–08	1.758E–14	1.908E–07	0	8.752E–07	0
	Std	1.398E–28	1.843E–07	5.605E–16	1.091E–07	1.276E–07	2.505E–14	1.968E–07	0	2.515E–06	0
	Best	0	1.080E–10	1.936E–17	6.619E–10	7.637E–13	1.720E–17	1.068E–10	0	9.635E–11	0
	Worst	0	4.938E–07	1.836E–15	3.719E–07	4.139E–07	8.365E–14	5.622E–07	0	1.120E–05	0
	Median	0	1.539E–08	3.969E–16	3.808E–08	1.646E–08	1.046E–14	1.049E–07	0	2.605E–07	0

**Table 8:** Evaluation results of recent algorithms for the functions F1 to F15.

${\text {Functions}}$		${\text {IGWO}}$	${\text {MWOA}}$	${\text {TLBO}}$	${\text {MTBO}}$	${\text {BWO}}$	${\text {HHO}}$	${\text {MGO}}$	${\text {SCSO}}$	${\text {LCA}}$
${\text {F1}}$	Mean	1.267E–60	2.658E–149	7.817E–183	8.815E–08	0	4.070E–189	9.615E–146	1.087E–223	0
	Std	3.214E–60	1.188E–148	0	3.859E–07	0	0	3.139E–145	0	0
	Best	7.650E–64	6.649E–167	7.903E–186	2.228E–13	0	5.979E–204	6.557E–163	1.355E–241	0
	Worst	1.309E–59	5.315E–148	3.515E–182	1.727E–06	0	7.215E–188	1.400E–144	2.174E–222	0
	Median	2.321E–61	1.459E–155	1.663E–183	5.748E–11	0	2.199E–195	1.211E–148	7.329E–237	0
${\text {F2}}$	Mean	4.710E–37	1.212E–103	7.484E–92	1.613E–01	1.480E–259	2.994E–97	4.230E–84	5.858E–123	0
	Std	4.200E–37	4.381E–103	1.407E–91	4.500E–01	0	9.789E–97	9.363E–84	2.239E–122	0
	Best	7.989E–38	5.944E–113	2.094E–93	7.179E–04	4.975E–269	1.223E–105	1.906E–89	1.096E–129	0
	Worst	1.412E–36	1.953E–102	6.495E–91	2.027E+00	2.954E–258	4.176E–96	3.041E–83	1.000E–121	0
	Median	2.867E–37	5.907E–109	3.171E–92	3.228E–02	3.320E–265	1.812E–100	2.422E–85	6.140E–127	0
${\text {F3}}$	Mean	3.280E–10	1.909E+04	5.176E–41	1.494E+02	0	4.056E–147	7.074E–18	8.844E–195	0
	Std	9.589E–10	8.656E+03	6.618E–41	1.916E+02	0	1.814E–146	2.752E–17	0	0
	Best	7.621E–14	2.357E+03	1.985E–44	7.682E+00	0	1.479E–180	8.967E–29	6.248E–214	0
	Worst	4.149E–09	3.869E+04	1.954E–40	6.363E+02	0	8.113E–146	1.228E–16	1.769E–193	0
	Median	5.332E–12	1.654E+04	2.223E–41	5.639E+01	0	1.584E–163	4.292E–23	1.076E–205	0
${\text {F4}}$	Mean	1.150E–11	4.610E+01	8.366E–75	1.007E+01	1.359E–252	1.031E–90	2.942E–47	8.172E–101	0
	Std	1.633E–11	2.958E+01	9.442E–75	3.024E+00	0	4.611E–90	8.594E–47	3.595E–100	0
	Best	9.717E–13	1.765E–01	4.521E–76	5.932E+00	1.728E–260	5.362E–103	4.449E–57	2.272E–111	0
	Worst	6.413E–11	9.001E+01	4.347E–74	2.032E+01	2.706E–251	2.062E–89	3.530E–46	1.609E–99	0
	Median	4.784E–12	5.984E+01	5.792E–75	9.785E+00	5.736E–257	5.788E–99	3.281E–50	2.826E–105	0
${\text {F5}}$	Mean	2.296E+01	2.700E+01	2.406E+01	6.069E+01	1.451E–13	1.817E–03	1.652E–30	2.800E+01	0
	Std	1.971E–01	3.619E–01	6.934E–01	3.333E+01	5.440E–13	1.658E–03	3.857E–30	7.589E–01	0
	Best	2.261E+01	2.639E+01	2.267E+01	7.835E+00	1.344E–19	2.044E–05	0	2.711E+01	0
	Worst	2.337E+01	2.796E+01	2.530E+01	1.315E+02	2.445E–12	6.287E–03	1.514E–29	2.887E+01	0
	Median	2.294E+01	2.692E+01	2.417E+01	7.178E+01	1.104E–15	1.547E–03	0	2.804E+01	0
${\text {F6}}$	Mean	4.734E–06	5.520E–02	1.071E–10	1.279E–06	9.033E–28	4.384E–05	2.708E–15	1.930E+00	0
	Std	2.118E–06	6.349E–02	2.568E–10	3.912E–06	1.751E–27	6.932E–05	1.002E–14	4.083E–01	0
	Best	2.518E–06	9.860E–03	2.253E–14	8.527E–13	3.236E–31	1.710E–07	1.594E–19	1.260E+00	0
	Worst	1.080E–05	2.690E–01	1.085E–09	1.273E–05	6.916E–27	2.335E–04	4.504E–14	2.771E+00	0
	Median	4.191E–06	3.477E–02	2.511E–12	5.278E–10	1.169E–28	1.194E–05	8.987E–17	1.898E+00	0
${\text {F7}}$	Mean	1.415E–03	1.040E–03	5.429E–04	5.460E–02	7.321E–05	1.016E–04	2.255E–04	1.041E–04	2.969E–05
	Std	4.332E–04	1.185E–03	2.086E–04	2.505E–02	5.329E–05	1.985E–04	1.330E–04	1.573E–04	1.651E–05
	Best	7.231E–04	2.755E–05	1.847E–04	1.888E–02	5.563E–06	3.240E–06	1.935E–05	2.874E–06	1.534E–05
	Worst	2.576E–03	4.881E–03	8.562E–04	1.015E–01	1.673E–04	9.265E–04	4.618E–04	6.886E–04	6.860E–05
	Median	1.389E–03	8.353E–04	5.748E–04	5.118E–02	6.474E–05	4.619E–05	2.215E–04	4.609E–05	2.213E–05
${\text {F8}}$	Mean	–9.748E+03	–9.080E+03	–8.154E+03	–7.601E+03	–1.257E+04	–1.257E+04	–1.257E+04	–6.920E+03	–1.26E+04
	Std	1.085E+03	1.220E+03	5.918E+02	1.025E+03	3.732E–12	3.002E–01	1.430E–10	5.515E+02	1.87E–12
	Best	–1.095E+04	–1.257E+04	–9.175E+03	–8.789E+03	–1.257E+04	–1.257E+04	–1.257E+04	–7.872E+03	–1.26E+04
	Worst	–7.319E+03	–7.725E+03	–7.268E+03	–4.414E+03	–1.257E+04	–1.257E+04	–1.257E+04	–5.906E+03	–1.26E+04
	Median	–1.007E+04	–8.874E+03	–8.127E+03	–7.804E+03	–1.257E+04	–1.257E+04	–1.257E+04	–6.923E+03	–1.26E+04

${\text {F9}}$	Mean	1.814E+01	0	1.380E+01	3.661E+01	0	0	0	0	0
	Std	7.141E+00	0	6.331E+00	8.832E+00	0	0	0	0	0
	Best	3.227E+00	0	0	1.990E+01	0	0	0	0	0
	Worst	3.060E+01	0	2.687E+01	5.472E+01	0	0	0	0	0
	Median	1.908E+01	0	1.492E+01	3.681E+01	0	0	0	0	0
${\text {F10}}$	Mean	1.341E–14	2.931E–15	6.128E–15	9.046E+00	4.441E–16	4.441E–16	1.155E–15	4.441E–16	4.441E–16
	Std	2.383E–15	2.334E–15	1.786E–15	1.807E+00	2.023E–31	2.023E–31	1.458E–15	2.023E–31	1.012E–31
	Best	7.550E–15	4.441E–16	3.997E–15	4.383E+00	4.441E–16	4.441E–16	4.441E–16	4.441E–16	4.441E–16
	Worst	1.465E–14	7.550E–15	7.550E–15	1.173E+01	4.441E–16	4.441E–16	3.997E–15	4.441E–16	4.441E–16
	Median	1.465E–14	3.997E–15	7.550E–15	9.123E+00	4.441E–16	4.441E–16	4.441E–16	4.441E–16	4.441E–16
${\text {F11}}$	Mean	1.180E–02	3.307E–03	0	1.032E–01	0	0	0	0	0
	Std	1.467E–02	1.479E–02	0	1.283E–01	0	0	0	0	0
	Best	0	0	0	7.396E–03	0	0	0	0	0
	Worst	5.880E–02	6.614E–02	0	5.940E–01	0	0	0	0	0
	Median	8.627E–03	0	0	6.851E–02	0	0	0	0	0
${\text {F12}}$	Mean	2.283E–07	1.130E–02	1.037E–02	1.770E+00	1.892E–25	1.149E–06	1.571E–32	6.675E–02	1.570E–32
	Std	7.663E–08	1.370E–02	3.191E–02	1.828E+00	2.749E–25	1.161E–06	5.616E–48	2.410E–02	2.808E–48
	Best	7.831E–08	7.591E–04	4.222E–15	2.516E–04	8.837E–28	1.159E–08	1.571E–32	2.695E–02	1.570E–32
	Worst	3.967E–07	6.256E–02	1.037E–01	7.249E+00	9.150E–25	3.925E–06	1.571E–32	1.147E–01	1.570E–32
	Median	2.256E–07	8.073E–03	3.779E–13	1.242E+00	3.747E–26	7.914E–07	1.571E–32	6.303E–02	1.570E–32
${\text {F13}}$	Mean	1.469E–02	2.273E–01	6.869E–02	1.035E+01	1.333E–24	2.155E–05	1.350E–32	2.392E+00	1.349E–32
	Std	3.586E–02	1.625E–01	6.716E–02	7.885E+00	2.261E–24	3.914E–05	2.808E–48	2.809E–01	5.616E–48
	Best	1.902E–06	1.967E–02	8.994E–12	1.099E–02	2.506E–28	2.016E–07	1.350E–32	1.752E+00	1.349E–32
	Worst	9.889E–02	6.187E–01	1.956E–01	2.573E+01	8.684E–24	1.604E–04	1.350E–32	2.789E+00	1.349E–32
	Median	7.286E–06	2.092E–01	4.224E–02	8.715E+00	5.157E–25	8.669E–06	1.350E–32	2.495E+00	1.349E–32
${\text {F14}}$	Mean	0.9980038	2.5697519	0.9980038	0.9980038	0.9980038	1.2445459	0.9980038	3.6494023	0.998
	Std	2.278E–16	2.9462685	2.278E–16	2.278E–16	1.161E–12	1.102569	2.278E–16	3.476762	0
	Best	0.9980038	0.9980038	0.9980038	0.9980038	0.9980038	0.9980038	0.9980038	0.9980038	0.998
	Worst	0.9980038	10.763180	0.9980038	0.9980038	0.9980038	5.9288451	0.9980038	12.670505	0.998
	Median	0.9980038	0.9980038	0.9980038	0.9980038	0.9980038	0.9980038	0.9980038	2.9821051	0.998
${\text {F15}}$	Mean	3.533E–04	6.378E–04	1.312E–03	1.579E–03	3.448E–04	3.298E–04	4.448E–04	3.784E–04	3.109E–04
	Std	2.048E–04	4.774E–04	4.484E–03	4.430E–03	3.730E–05	1.879E–05	3.355E–04	2.083E–04	3.534E–06
	Best	3.075E–04	3.085E–04	3.075E–04	3.075E–04	3.079E–04	3.090E–04	3.075E–04	3.075E–04	3.087E–04
	Worst	1.223E–03	2.180E–03	2.036E–02	2.036E–02	4.228E–04	3.773E–04	1.223E–03	1.223E–03	3.185E–04
	Median	3.075E–04	4.489E–04	3.075E–04	6.100E–04	3.306E–04	3.292E–04	3.075E–04	3.075E–04	3.089E–04

**Table 9:** Evaluation results of recent algorithms for the functions F16 to F30.

${\text {Functions}}$		${\text {IGWO}}$	${\text {MWOA}}$	${\text {TLBO}}$	${\text {MTBO}}$	${\text {BWO}}$	${\text {HHO}}$	${\text {MGO}}$	${\text {SCSO}}$	${\text {LCA}}$
${\text {F16}}$	Mean	–1.031628453	–1.031628453	–1.031628453	–1.031628453	–1.031519453	–1.031628453	–1.031628453	–1.031628453	$-1.0316$
	Std	0	1.42155E–10	0	0	0.000138132	4.46706E–11	0	1.00851E–10	$4.5E-16$
	Best	–1.031628453	–1.031628453	–1.031628453	–1.031628453	–1.031622955	–1.031628453	–1.031628453	–1.031628453	$-1.0316$
	Worst	–1.031628453	–1.031628453	–1.031628453	–1.031628453	–1.031170848	–1.031628453	–1.031628453	–1.031628453	$-1.0316$
	Median	–1.031628453	–1.031628453	–1.031628453	–1.031628453	–1.031572739	–1.031628453	–1.031628453	–1.031628453	$-1.0316$
${\text {F17}}$	Mean	0.397887358	0.397888203	0.397887358	0.397887358	0.399540375	0.397887548	0.397887358	0.397887378	0.397931
	Std	1.13906E–16	1.2932E–06	1.139E–16	1.139E–16	0.001699	3.986E–07	1.139E–16	2.98963E–08	3.596E–05
	Best	0.397887358	0.397887358	0.397887358	0.397887358	0.398016352	0.397887358	0.397887358	0.397887358	0.39789
	Worst	0.397887358	0.397892503	0.397887358	0.397887358	0.403096172	0.397888927	0.397887358	0.397887475	0.39799
	Median	0.397887358	0.397887744	0.397887358	0.397887358	0.398783676	0.397887367	0.397887358	0.397887367	0.39793
${\text {F18}}$	Mean	3	3.00002562	3	3	3.806062772	3.000000021	3	3.000001943	3
	Std	9.11252E–16	7.2916E–05	9.11252E–16	9.11252E–16	1.051940928	5.51285E–08	2.55313E–15	1.9466E–06	0
	Best	3	3	3	3	3.044339174	3	3	3.000000054	3
	Worst	3	3.000330139	3	3	6.691037397	3.00000023	3	3.000007774	3
	Median	3	3.000004633	3	3	3.233396062	3	3	3.000001323	3
${\text {F19}}$	Mean	–3.862782148	–3.859605134	–0.300478907	–3.862782148	–3.859180338	–3.861917162	–3.862782148	–3.860081434	–3.8581
	Std	9.11252E–16	0.003485126	5.69532E–17	9.11252E–16	0.002706882	0.001167888	9.11252E–16	0.003779125	0.00417
	Best	–3.862782148	–3.862759593	–0.300478907	–3.862782148	–3.862576798	–3.862781882	–3.862782148	–3.862782145	–3.8617
	Worst	–3.862782148	–3.854717509	–0.300478907	–3.862782148	–3.854550597	–3.858036371	–3.862782148	–3.854900607	–3.8619
	Median	–3.862782148	–3.861237973	–0.300478907	–3.862782148	–3.85950753	–3.862403254	–3.862782148	–3.862778104	–3.8617
${\text {F20}}$	Mean	–3.304161203	–3.252259292	–3.316032459	–3.268493267	–3.293716407	–3.160182479	–3.250659299	–3.227916732	–2.7719
	Std	0.043556204	0.082465905	0.026581182	0.060685164	0.034408508	0.106687397	0.059758618	0.084299104	0.18520
	Best	–3.321995172	–3.32197519	–3.321995172	–3.321995172	–3.312217711	–3.300817033	–3.321995172	–3.321995075	–2.9438
	Worst	–3.20310205	–3.096228164	–3.20310205	–3.20310205	–3.194823213	–2.923391951	–3.20310205	–3.091737365	–2.5042
	Median	–3.321995172	–3.317268843	–3.321995172	–3.321995172	–3.305296578	–3.156799959	–3.20310205	–3.202835139	–2.821
${\text {F21}}$	Mean	–10.13108689	–7.359709904	–9.898299582	–5.784013667	–10.15319942	–5.054494211	–10.15319968	–5.747122367	$-10.1532$
	Std	0.098891393	2.947520421	1.139947891	3.696588343	4.18419E–07	0.000578814	1.8225E–15	2.48879422	0
	Best	–10.15319968	–10.15270958	–10.15319968	–10.15319968	–10.15319966	–5.055175115	–10.15319968	–10.15319327	$-10.1532$
	Worst	–9.710943925	–2.629698008	–5.055197729	–2.630471668	–10.15319809	–5.05296594	–10.15319968	–0.880981825	$-10.1532$
	Median	–10.15319968	–7.596502228	–10.15319968	–2.682860396	–10.1531996	–5.054720408	–10.15319968	–5.055197661	$-10.1532$
${\text {F22}}$	Mean	–10.40294057	–7.341118508	–9.797225531	–7.895871968	–10.40293938	–5.605019424	–10.40294057	–5.666829108	–10.4029
	Std	4.62626E–10	2.866237264	1.874962926	3.52229388	1.45206E–06	1.595295236	0	2.744069867	3.6E–15
	Best	–10.40294057	–10.4027869	–10.40294057	–10.40294057	–10.40294056	–10.35157951	–10.40294057	–10.40293898	–10.4029
	Worst	–10.40294056	–3.723326686	–3.724300347	–2.751933564	–10.40293489	–5.082777759	–10.40294057	–0.909805029	–10.4029
	Median	–10.40294057	–5.087661843	–10.40294057	–10.40294057	–10.40293984	–5.087250905	–10.40294057	–5.087671663	–10.4029
${\text {F23}}$	Mean	–10.53640982	–6.987815674	–10.26030948	–8.66508135	–10.53640888	–5.6249448	–10.53640982	–5.942023398	$-10.5364$
	Std	2.28214E–14	2.673670225	1.234758254	3.331541553	8.68555E–07	1.531385105	5.46751E–15	1.980191343	5.467E–15
	Best	–10.53640982	–10.53638074	–10.53640982	–10.53640982	–10.53640976	–10.19458146	–10.53640982	–10.53640356	–10.5364
	Worst	–10.53640982	–4.485847579	–5.014403031	–2.806630721	–10.53640667	–5.125527989	–10.53640982	–5.128480412	–10.5364
	Median	–10.53640982	–5.128479011	–10.53640982	–10.53640982	–10.53640921	–5.127701525	–10.53640982	–5.128480715	–10.5364

${\text {F24}}$	Mean	$-5$	$-5$	$-5$	$-5$	$-5$	$-5$	$-5$	$-5$	$-5$
	Std	0	0	0	0	0	0	0	0	0
	Best	$-5$	$-5$	$-5$	$-5$	$-5$	$-5$	$-5$	$-5$	$-5$
	Worst	$-5$	$-5$	$-5$	$-5$	$-5$	$-5$	$-5$	$-5$	$-5$
	Median	$-5$	$-5$	$-5$	$-5$	$-5$	$-5$	$-5$	$-5$	$-5$
${\text {F25}}$	Mean	1.671E–61	1.337E–148	1.963E–183	3.795E–09	0	1.771E–186	1.508E–147	5.544E–228	0
	Std	3.050E–61	5.971E–148	0	1.322E–08	0	0	6.477E–147	0	0
	Best	1.497E–63	1.084E–164	4.313E–186	2.779E–13	0	3.399E–212	1.932E–157	1.275E–246	0
	Worst	1.241E–60	2.670E–147	2.723E–182	5.927E–08	0	3.503E–185	2.901E–146	1.094E–226	0
	Median	4.276E–62	4.140E–155	2.278E–184	3.309E–11	0	1.950E–200	9.140E–153	1.215E–238	0
${\text {F26}}$	Mean	8.274E–23	7.621E–02	0	0	9.081E–04	1.070E–12	1.938E–31	6.650E–10	2.591E–04
	Std	3.325E–22	2.346E–01	0	0	0	1.322E–03	3.213E–12	5.372E–31	9.166E–10
	Best	8.971E–27	3.412E–16	0	0	1.505E–05	0	0	2.115E–13	2.271E–04
	Worst	1.493E–21	7.621E–01	0	0	4.928E–03	1.353E–11	2.132E–30	3.597E–09	5.015E–04
	Median	1.031E–24	1.173E–11	0	0	2.729E–04	5.970E–15	0	3.210E–10	2.271E–04
${\text {F27}}$	Mean	–1	–0.999999896	–1	–1	–1	–0.999998796	–1	–0.999999997	–1
	Std	0	2.374E–07	0	0	0	0	1.675E–06	0	3.38776E–09
	Best	–1	–1	–1	–1	–1	–0.999999999	–1	–1	–1
	Worst	–1	–0.99999904	–1	–1	–1	–0.999994265	–1	–0.999999985	–1
	Median	–1	–0.999999989	–1	–1	–1	–0.999999678	–1	–0.999999998	–1
${\text {F28}}$	Mean	8.488E–307	0	8.086E–268	3.391E–117	0	2.921E–236	0	0	0
	Std	0	0	0	1.497E–116	0	0	0	0	0
	Best	0	0	1.375E–281	5.398E–132	0	1.895E–295	0	0	0
	Worst	5.093E–306	0	1.617E–266	6.700E–116	0	5.765E–235	0	0	0
	Median	0	0	3.187E–275	3.618E–126	0	5.392E–275	0	0	0
${\text {F29}}$	Mean	4.273E–05	1.384E+00	4.005E–10	1.466E–03	9.976E–04	9.292E–05	2.893E–30	7.892E–01	0
	Std	3.582E–05	1.835E+00	7.816E–10	3.494E–03	1.151E–03	1.001E–04	3.845E–30	7.021E–01	0
	Best	6.971E–06	1.955E–04	2.523E–13	1.704E–07	1.114E–04	5.153E–07	0	5.952E–03	0
	Worst	1.216E–04	7.788E+00	3.047E–09	1.457E–02	4.900E–03	3.890E–04	1.438E–29	1.571E+00	0
	Median	2.632E–05	8.529E–01	5.898E–11	5.418E–05	5.134E–04	7.440E–05	1.597E–30	1.030E+00	0
${\text {F30}}$	Mean	–356.4	–331.1999889	–356.4	–356.4	–359.9965549	–345.6	–360	–359.9999999	$-360$
	Std	16.09968944	36.18896836	16.09968944	16.09968944	0.002920961	29.54817654	0	7.02304E–08	0
	Best	–360	–360	–360	–360	–359.9999635	–360	–360	–360	$-360$
	Worst	–288	–288	–288	–288	–359.989665	–288	–360	–359.9999998	$-360$
	Median	–360	–359.999978	–360	–360	–359.9974954	–360	–360	–360	$-360$

**Table 10:** Evaluation results of recent algorithms for the functions F31 to F51.

${\text {Functions}}$		${\text {IGWO}}$	${\text {MWOA}}$	${\text {TLBO}}$	${\text {MTBO}}$	${\text {BWO}}$	${\text {HHO}}$	${\text {MGO}}$	${\text {SCSO}}$	${\text {LCA}}$
${\text {F31}}$	Mean	–2600	–2519.999983	–2600	–2580	–2593.528239	–2510	–2600	–2600	$-2600$
	Std	1.119E–11	1.005E+02	0	61.55870113	6.035411821	102.0835571	0	3.49179E–07	0.6545
	Best	–2600	–2600	–2600	–2600	–2599.959733	–2600	–2600	–2600	$-2600$
	Worst	–2600	–2399.999998	–2600	–2400	–2576.88762	–2400	–2600	–2599.999998	$-2600$
	Median	–2600	–2599.999954	–2600	–2600	–2594.802697	–2600	–2600	–2600	$-2600$
${\text {F32}}$	Mean	1.513E–125	4.062E–158	9.256E–225	2.301E–63	0	3.147E–188	1.340E–234	1.891E–261	0
	Std	6.191E–125	0	0	9.040E–63	0	0	0	0	0
	Best	1.384E–131	4.719E–173	2.284E–228	1.763E–70	0	3.332E–212	1.215E–252	1.143E–286	0
	Worst	2.777E–124	6.563E–157	1.145E–223	4.036E–62	0	6.195E–187	2.679E–233	3.782E–260	0
	Median	4.947E–128	3.070E–164	1.258E–226	2.639E–67	0	2.077E–200	8.613E–246	7.253E–276	0
${\text {F33}}$	Mean	2.328E–05	2.007E–06	1.240E–06	3.695E–03	0	5.520E–189	7.414E–20	2.249E–205	0
	Std	2.341E–05	3.478E–06	2.519E–06	7.070E–03	0	0	3.314E–19	0	0
	Best	6.034E–07	6.492E–24	2.408E–09	1.681E–04	0	1.324E–209	7.169E–79	1.748E–223	0
	Worst	9.158E–05	1.354E–05	8.784E–06	2.862E–02	0	1.017E–187	1.482E–18	2.604E–204	0
	Median	1.398E–05	4.760E–07	9.067E–08	6.301E–04	0	3.412E–196	1.955E–39	2.576E–209	0
${\text {F34}}$	Mean	4.207E–08	9.121E–05	1.095E–09	1.103E–07	9.939E–04	1.374E–06	0	2.354E–09	0
	Std	8.795E–08	3.906E–04	4.863E–09	4.815E–07	1.486E–03	5.915E–06	0	6.571E–09	0
	Best	9.802E–13	2.739E–13	1.758E–29	7.216E–29	5.463E–05	6.312E–19	0	1.378E–12	0
	Worst	3.007E–07	1.750E–03	2.176E–08	2.155E–06	6.081E–03	2.649E–05	0	2.880E–08	0
	Median	2.558E–09	7.092E–08	9.042E–15	8.723E–18	3.248E–04	7.162E–11	0	5.137E–11	0
${\text {F35}}$	Mean	0	0	0	0	0	0	0	0	0
	Std	0	0	0	0	0	0	0	0	0
	Best	0	0	0	0	0	0	0	0	0
	Worst	0	0	0	0	0	0	0	0	0
	Median	0	0	0	0	0	0	0	0	0
${\text {F36}}$	Mean	0	1.846E–04	0	0	1.316E–02	1.351E–05	0	1.507E–09	0
	Std	0	1.829E–04	0	0	1.094E–02	1.962E–05	0	1.625E–09	0
	Best	0	1.547E–06	0	0	2.826E–04	5.889E–07	0	1.735E–10	0
	Worst	0	6.420E–04	0	0	4.312E–02	8.616E–05	0	6.555E–09	0
	Median	0	1.277E–04	0	0	1.146E–02	7.420E–06	0	9.423E–10	0
${\text {F37}}$	Mean	–1.801E+00	–1.761E+00	–1.80130341	–1.80130341	–1.792931404	–1.801303341	–1.80130341	–1.801303393	–1.8013
	Std	2.278E–16	1.792E–01	2.27813E–16	2.27813E–16	0.007577534	1.0454E–07	2.27813E–16	2.12997E–08	2.27813E–16
	Best	–1.801E+00	–1.801E+00	–1.80130341	–1.80130341	–1.801118771	–1.80130341	–1.80130341	–1.80130341	$-1.8013$
	Worst	–1.801E+00	–1.000E+00	–1.80130341	–1.80130341	–1.778829412	–1.801303089	–1.80130341	–1.801303338	$-1.8013$
	Median	–1.801E+00	–1.801E+00	–1.80130341	–1.80130341	–1.795093528	–1.801303403	–1.80130341	–1.801303402	$-1.8013$
${\text {F38}}$	Mean	–4.688E+00	–3.931E+00	–4.599170064	–4.525632059	–4.533729173	–3.972500532	–4.619902565	–3.838872176	–3.25754
	Std	7.889E–06	4.832E–01	0.077337752	0.238929809	0.070670424	0.444660327	0.075397429	0.575059478	0.126028177
	Best	–4.688E+00	–4.685E+00	–4.687658179	–4.687658179	–4.642856136	–4.644847481	–4.687658179	–4.687648188	–3.426
	Worst	–4.688E+00	–2.985E+00	–4.495893207	–3.694589796	–4.424495995	–3.42015395	–4.483135734	–2.79789007	–3.0562
	Median	–4.688E+00	–3.709E+00	–4.644837995	–4.645895026	–4.518993699	–3.972060041	–4.645895368	–3.694589523	–3.22785
${\text {F39}}$	Mean	–9.068E+00	–6.008E+00	–9.01522002	–8.732242041	–6.070391308	–6.110441526	–9.046555997	–6.43592972	–5.66115
	Std	3.435E–01	6.219E–01	0.321545056	0.504127326	0.508509705	0.696997939	0.275474881	0.98654883	0.431730696
	Best	–9.550E+00	–7.019E+00	–9.425864026	–9.343178782	–7.736882995	–7.190986668	–9.384751108	–8.79614711	–6.3366
	Worst	–7.953E+00	–4.714E+00	–8.356104448	–7.569585751	–5.446550181	–4.883048814	–8.458962888	–4.650984325	–5.2273
	Median	–9.098E+00	–6.045E+00	–9.079140438	–8.78895874	–6.008318134	–6.078816149	–9.066799789	–6.312001237	–5.4497
${\text {F40}}$	Mean	0	0	0	0	0	0	0	0	0
	Std	0	0	0	0	0	0	0	0	0
	Best	0	0	0	0	0	0	0	0	0
	Worst	0	0	0	0	0	0	0	0	0
	Median	0	0	0	0	0	0	0	0	0

${\text {F41}}$	Mean	0	0	0	0	0	0	0	0	0
	Std	0	0	0	0	0	0	0	0	0
	Best	0	0	0	0	0	0	0	0	0
	Worst	0	0	0	0	0	0	0	0	0
	Median	0	0	0	0	0	0	0	0	0
${\text {F42}}$	Mean	0	9.714E–17	0	0	0	0	0	0	0
	Std	0	1.669E–16	0	0	0	0	0	0	0
	Best	0	0	0	0	0	0	0	0	0
	Worst	0	5.551E–16	0	0	0	0	0	0	0
	Median	0	0	0	0	0	0	0	0	0
${\text {F43}}$	Mean	–24.99985224	–24.99999928	–24.99871775	–24.99822699	–24.99994621	–24.99999994	–25	–24.99999945	$-25$
	Std	0.000327874	2.16995E–06	0.000874902	0.002140763	7.30727E–05	1.73922E–07	0	1.09684E–06	0
	Best	–24.99999926	–25	–24.9999503	–24.99999999	–24.99999993	–25	–25	–25	$-25$
	Worst	–24.99851988	–24.99999037	–24.99727191	–24.99074036	–24.99970548	–24.99999924	–25	–24.99999561	$-25$
	Median	–24.99995762	–24.99999998	–24.99867017	–24.99867989	–24.99996887	–25	–25	–24.99999998	$-25$
${\text {F44}}$	Mean	0	0	0	0	0	0	0	0	0
	Std	0	0	0	0	0	0	0	0	0
	Best	0	0	0	0	0	0	0	0	0
	Worst	0	0	0	0	0	0	0	0	0
	Median	0	0	0	0	0	0	0	0	0
${\text {F45}}$	Mean	0	4.735E–12	0	0	0.000462133	0	0	3.76103E–08	0
	Std	0	1.073E–11	0	0	0.000831292	0	0	5.74829E–08	0
	Best	0	2.251E–16	0	0	1.20274E–06	0	0	1.26264E–10	0
	Worst	0	3.764E–11	0	0	0.003421027	0	0	2.1425E–07	0
	Median	0	1.066E–13	0	0	7.17244E–05	0	0	9.08508E–09	0
${\text {F46}}$	Mean	–1.034	–1.014105252	–1.034	–1.034	–1.029926715	–1.034	–1.034	–1.008149999	$-1.0341$
	Std	0	0.088972017	0	0	0.006712129	1.51796E–11	0	0.115604714	0
	Best	–1.034	–1.034	–1.034	–1.034	–1.033991487	–1.034	–1.034	–1.034	$-1.034$
	Worst	–1.034	–0.636105042	–1.034	–1.034	–1.006485061	–1.034	–1.034	–0.517	$-1.034$
	Median	–1.034	–1.034	–1.034	–1.034	–1.032821904	–1.034	–1.034	–1.034	$-1.034$
${\text {F47}}$	Mean	–4.825	–3.868825722	–2.234366896	–4.776750076	–4.799467405	–4.82143072	–4.825	–4.017799574	–4.62031
	Std	9.11252E–16	0.680018044	0.062194668	0.215780221	0.014149513	0.002303213	9.11252E–16	0.649473866	0.288855
	Best	–4.825	–4.824984594	–2.248633351	–4.825	–4.823591842	–4.824955173	–4.825	–4.824999894	–4.6849
	Worst	–4.825	–2.894993803	–1.970212321	–3.860001516	–4.775673138	–4.817382988	–4.825	–2.865683603	–3.3931
	Median	–4.825	–3.859971954	–2.248633351	–4.825	–4.801538318	–4.821446897	–4.825	–3.860003321	–4.6849
${\text {F48}}$	Mean	–8.759999994	–5.087587315	–8.453450888	–7.806566271	–4.473652447	–7.395415441	–8.7162	–5.814601868	–5.838515
	Std	1.02509E–08	0.708116778	0.428608666	0.53971358	0.270184415	0.540574512	0.195879555	0.843970291	0.2436643
	Best	–8.76	–6.130314235	–8.76	–8.76	–4.937067039	–8.667871498	–8.76	–7.010837453	–5.893
	Worst	–8.759999958	–3.504007998	–7.884000001	–7.009513304	–3.918868486	–6.856856991	–7.884	–3.989170484	–4.8033
	Median	–8.759999999	–5.241225781	–8.76	–7.884009291	–4.483067125	–7.190286478	–8.76	–6.022940734	–5.893
${\text {F49}}$	Mean	0	1.899E–13	0	2.426E–27	7.656E–06	0	0	7.467E–10	0
	Std	0	3.730E–13	0	1.085E–26	2.051E–05	0	0	1.196E–09	0
	Best	0	1.140E–17	0	0	8.644E–10	0	0	1.181E–12	0
	Worst	0	1.408E–12	0	4.852E–26	8.843E–05	0	0	5.049E–09	0
	Median	0	1.728E–14	0	0	7.588E–07	0	0	2.711E–10	0
${\text {F50}}$	Mean	0	1.926E–13	0	0	5.142E–05	0	0	2.632E–09	0
	Std	0	4.177E–13	0	0	7.299E–05	0	0	6.959E–09	0
	Best	0	1.223E–16	0	0	3.136E–09	0	0	6.075E–14	0
	Worst	0	1.793E–12	0	0	2.227E–04	0	0	3.115E–08	0
	Median	0	1.516E–14	0	0	1.174E–05	0	0	3.446E–10	0
${\text {F51}}$	Mean	0	1.331E–13	0	0	1.181E–06	0	0	2.443E–09	0
	Std	0	2.884E–13	0	0	2.723E–06	0	0	8.081E–09	0
	Best	0	2.803E–17	0	0	4.846E–10	0	0	2.021E–13	0
	Worst	0	1.185E–12	0	0	1.194E–05	0	0	3.535E–08	0
	Median	0	1.094E–15	0	0	7.483E–08	0	0	1.222E–10	0

### Comparison results of LCA with well-known & top-performing algorithms for 51 benchmark functions

The comparison results of 51 functions are shown in Tables 5, 6 and 7, where LCA delivers the global optimal results of all the algorithms on 38 functions and is competitive on the other functions. In LCA, 44 out of 51 benchmark function’s average results are the best. In PSO, 17 out of 51 benchmark functions provide the best average results. Moreover, LCA and PSO provide the same best average results on some functions such as *F*16, *F*18, *F*27, *F*35, *F*36, $F40-F46$, and *F*50. In TSA, 6 out of 51 benchmark function’s average results are the best. Furthermore, LCA and TSA provide the same best average results on some functions such as *F*16, *F*18, *F*35, *F*40, *F*41, and *F*44. In SSA, 6 out of 51 benchmark function’s average results are the best. Furthermore, LCA and SSA provide the same best average results on some functions such as *F*16, *F*18, *F*27, *F*43, and *F*46. In MVO, 10 out of 51 benchmark functions provide the best average results. Moreover, LCA and MVO provide the same best average results on some functions such as *F*16, *F*18, *F*24, *F*30, *F*37, *F*43, *F*44, and *F*46. In GWO, 12 out of 51 benchmark function’s average results are the best. Moreover, LCA and GWO provide the same best average results on some functions such as *F*16, *F*18, *F*24, *F*27, *F*35, *F*37, $F40-F42$, *F*44, and *F*46. In WOA, 14 out of 51 benchmark function’s average results are the best. Furthermore, LCA and WOA provide the same best average results on some functions such as *F*9, *F*11, *F*16, *F*18, *F*24, *F*27, *F*28, *F*35, *F*37, *F*40, *F*41, *F*43, *F*44, and *F*46. In GJO, 13 out of 51 benchmark functions provide the best average results. Moreover, LCA and GJO provide the same best average results on some functions such as *F*9, *F*11, *F*16, *F*18, *F*24, *F*27, *F*28, *F*35, *F*37, $F40-F42$, and *F*44. In LSHADE, 27 out of 51 benchmark function’s average results are the best. Furthermore, LCA and LSHADE provide the same best average results on some functions *F*16, *F*18, $F22-F24$, *F*27, *F*29, *F*30, $F34-F37$, $F40-F42$, $F44-F46$, and $F49-F51$. In CMAES, 11 out of 51 benchmark functions provide the best average results. Furthermore, LCA and CMAES provide the same best average results on some functions *F*11, *F*16, $F21-F24$, *F*27, *F*35, *F*36, and *F*46. The results of the implementation of LCA and nine competitor algorithms on the functions *F*1 to *F*15 are reported in Table 5. The simulation results show that LCA has been able to achieve global optimality in optimizing benchmark functions such as *F*1, *F*2, *F*3, *F*4, *F*5, *F*6, *F*8, *F*9, *F*11, *F*14, and *F*15. Furthermore, LCA performed better in optimizing benchmark functions *F*7, *F*10, *F*12, and *F*13. The optimization results show that LCA is the best of all the optimizers when compared to handling the functions from *F*1 to *F*15. The optimization results of the LCA, and nine competitor algorithms on the functions from *F*16 to *F*30 are presented in Table 6. The simulation results show that LCA has been able to achieve global optimality in optimizing the benchmark functions $F16-F18$, $F21-F25$, and $F27-F30$. Furthermore, LCA performed worst in optimizing the benchmark functions *F*19, *F*20, and *F*26. According to the simulation results, PSO has been able to provide the global optimal in optimizing the benchmark functions *F*19, and *F*26. The global optimal in the function *F*19 is provided by SSA. And then MVO algorithm provides the global optimal in the functions *F*19, and *F*20. The optimization results of the LCA algorithm and nine competitor algorithms on the functions from *F*31 to *F*51 are presented in Table 7. The simulation results show that LCA has been able to provide the global optimal in optimizing the benchmark functions $F31-F37$, $F40-F46$, and $F49-F51$. Furthermore, LCA performed worst in optimizing the benchmark functions *F*38, *F*39, *F*47, and *F*48. According to the simulation results, PSO has been able to provide the global optimal in optimizing benchmark functions *F*34, *F*36, *F*47, and *F*48. Analysis of the simulation results shows that the LCA algorithm has superior and much more competitive performance than the other nine compared algorithms.

### Comparison results of LCA with recent algorithms for 51 benchmark functions

The comparison results of 51 functions are shown in Tables 8, 9 and 10, where LCA delivers the global optimal results of all the algorithms on 38 functions and is competitive on the other functions. In LCA, 44 out of 51 benchmark function’s average results are the best. In IGWO, 22 out of 51 benchmark functions provide the best average results. Moreover, LCA and IGWO provide the same best average results on some functions such as *F*16, *F*18, *F*23, *F*24, *F*27, *F*31, $F35-F37$, $F40-F42$, $F44-F46$, and $F49-F51$. In MWOA, 7 out of 51 benchmark function’s average results are the best. Furthermore, LCA and MWOA provide the same best average results on some functions such as *F*9, *F*24, *F*28, *F*35, *F*40, *F*41, and *F*44. In TLBO, 20 out of 51 benchmark function’s average results are the best. Furthermore, LCA and TLBO provide the same best average results on some functions such as *F*10, *F*11, *F*16, *F*18, *F*24, *F*26, *F*27, *F*31, $F35-F37$, $F40-F42$, $F44-F46$, and $F49-F51$. In MTBO, 17 out of 51 benchmark functions provide the best average results. Moreover, LCA and MTBO provide the same best average results on some functions such as *F*16, *F*18, *F*24, *F*26, *F*27, $F35-F37$, $F40-F42$, $F44-F46$, and $F49-F51$. In BWO, 18 out of 51 benchmark function’s average results are the best. Moreover, LCA and BWO provide the same best average results on some functions such as *F*1, *F*3, $F8-F11$, *F*16, *F*18, *F*24, *F*25, *F*28, *F*32, *F*33, *F*35, *F*37, $F40-F42$, and *F*44. In HHO, 15 out of 51 benchmark functions provide the best average results. Furthermore, LCA and HHO provide the same best average results on some functions such as $F8-F11$, *F*24, *F*35, $F40-F42$, $F44-F46$, and $F49-F51$. In MGO, 27 out of 51 benchmark functions provide the best average results. Moreover, LCA and MGO provide the same best average results on some functions such as $F8-F11$, *F*16, *F*18, *F*23, *F*24, *F*27, *F*28, *F*30, *F*31, $F34-F37$, $F40-F47$, and $F49-F51$. In SCSO, 11 out of 51 benchmark functions provide the best average results. Furthermore, LCA and SCSO provide the same best average results on some functions $F9-F11$, *F*24, *F*28, *F*31, *F*35, $F40-F42$, and *F*44. The results of the implementation of LCA and eight competitor algorithms on the functions *F*1 to *F*15 are reported in Table 8. The simulation results show that LCA has been able to achieve global optimality in optimizing benchmark functions such as $F1-F6$, *F*8, *F*9, *F*11, *F*14, and *F*15. Furthermore, LCA performed better in optimizing benchmark functions *F*7, *F*10, *F*12, and *F*13. The optimization results show that LCA is the best of all the optimizers when compared to handling the functions from *F*1 to *F*15. The optimization results of the LCA, and eight competitor algorithms on the functions from *F*16 to *F*30 are presented in Table 9. The simulation results show that LCA has been able to achieve global optimality in optimizing the benchmark functions $F16-F18$, $F21-F25$, and $F27-F30$. Furthermore, LCA performed worst in optimizing the benchmark functions *F*19, *F*20, and *F*26. According to the simulation results, the TLBO has been able to provide the global optimal in optimizing the benchmark function *F*19 and *F*26. The MTBO has been able to provide the global optimal in optimizing the benchmark function *F*26. Then the SCSO has been able to provide the global optimal in optimizing the benchmark function *F*19. The optimization results of the LCA algorithm and eight competitor algorithms on the functions from *F*31 to *F*51 are presented in Table 10. The simulation results show that LCA has been able to provide the global optimal in optimizing the benchmark functions $F31-F37$, $F40-F46$, and $F49-F51$. Furthermore, LCA performed worst in optimizing the benchmark functions *F*38, *F*39, *F*47, and *F*48. According to the simulation results, IGWO has been able to provide the global optimal in optimizing the benchmark functions *F*38, *F*39, *F*47, and *F*48. Analysis of the simulation results shows that the LCA algorithm has superior and much more competitive performance than the other eight compared algorithms.

### Convergence analysis for 51 benchmark functions

**Figure 4:**
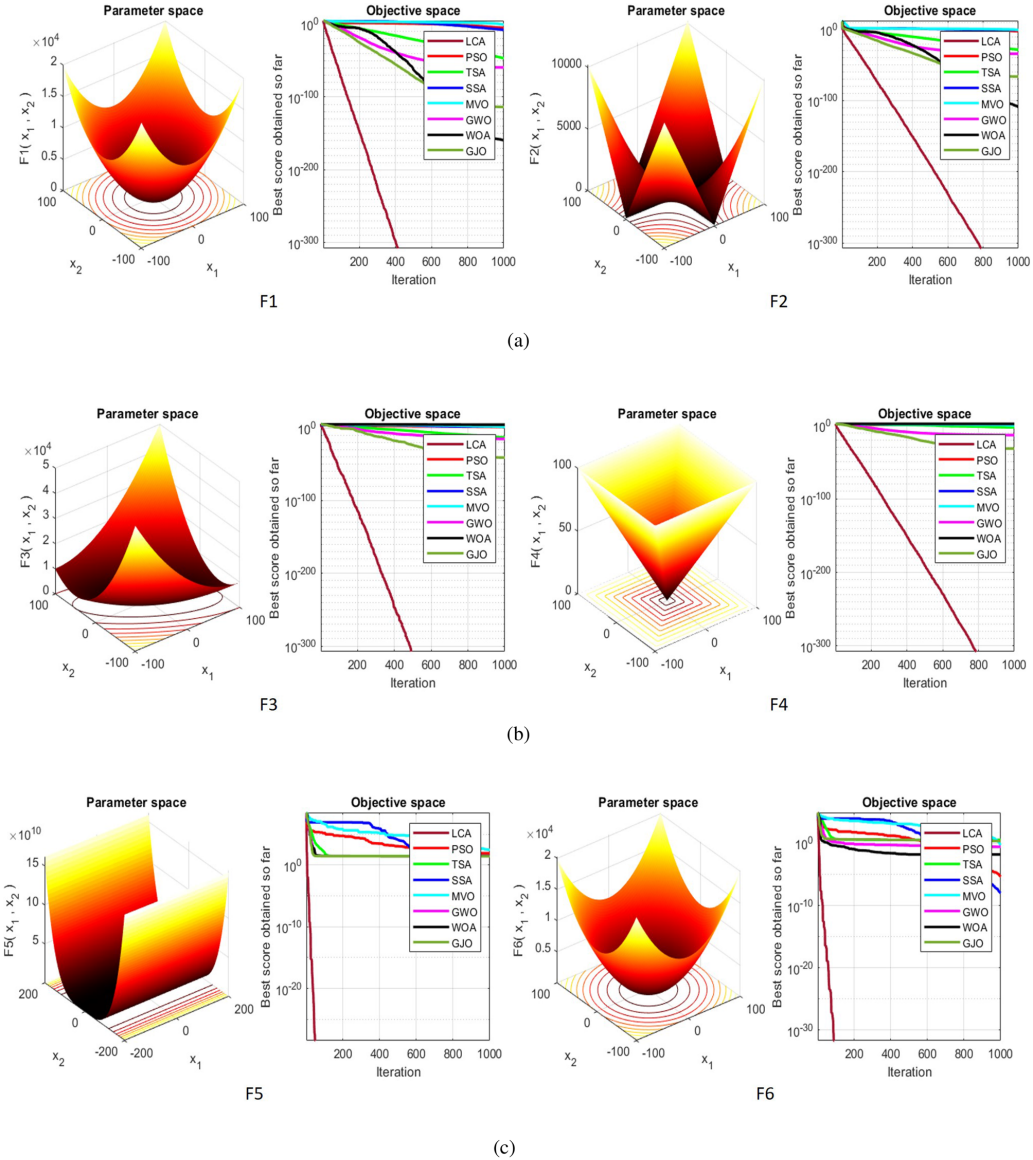

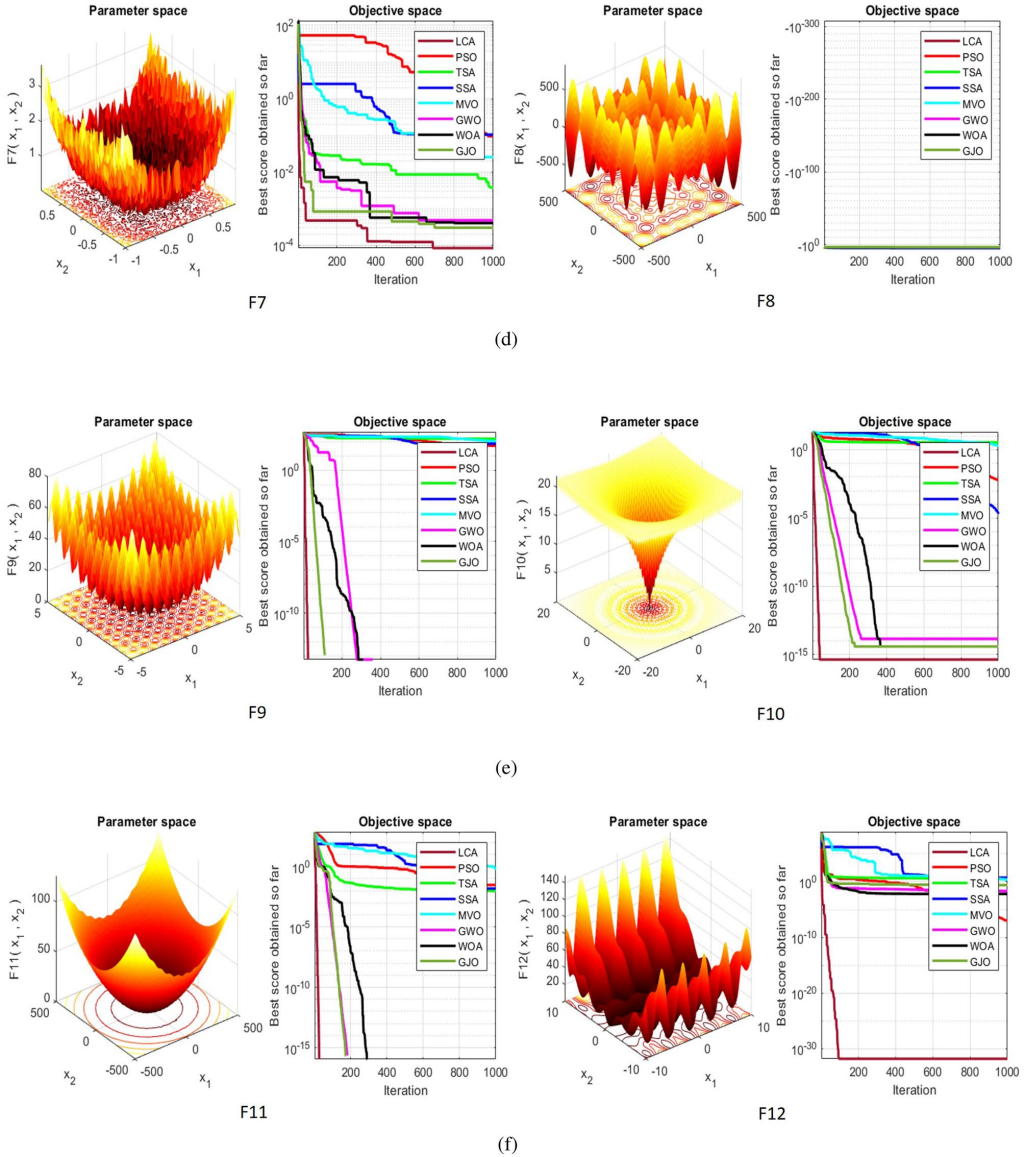

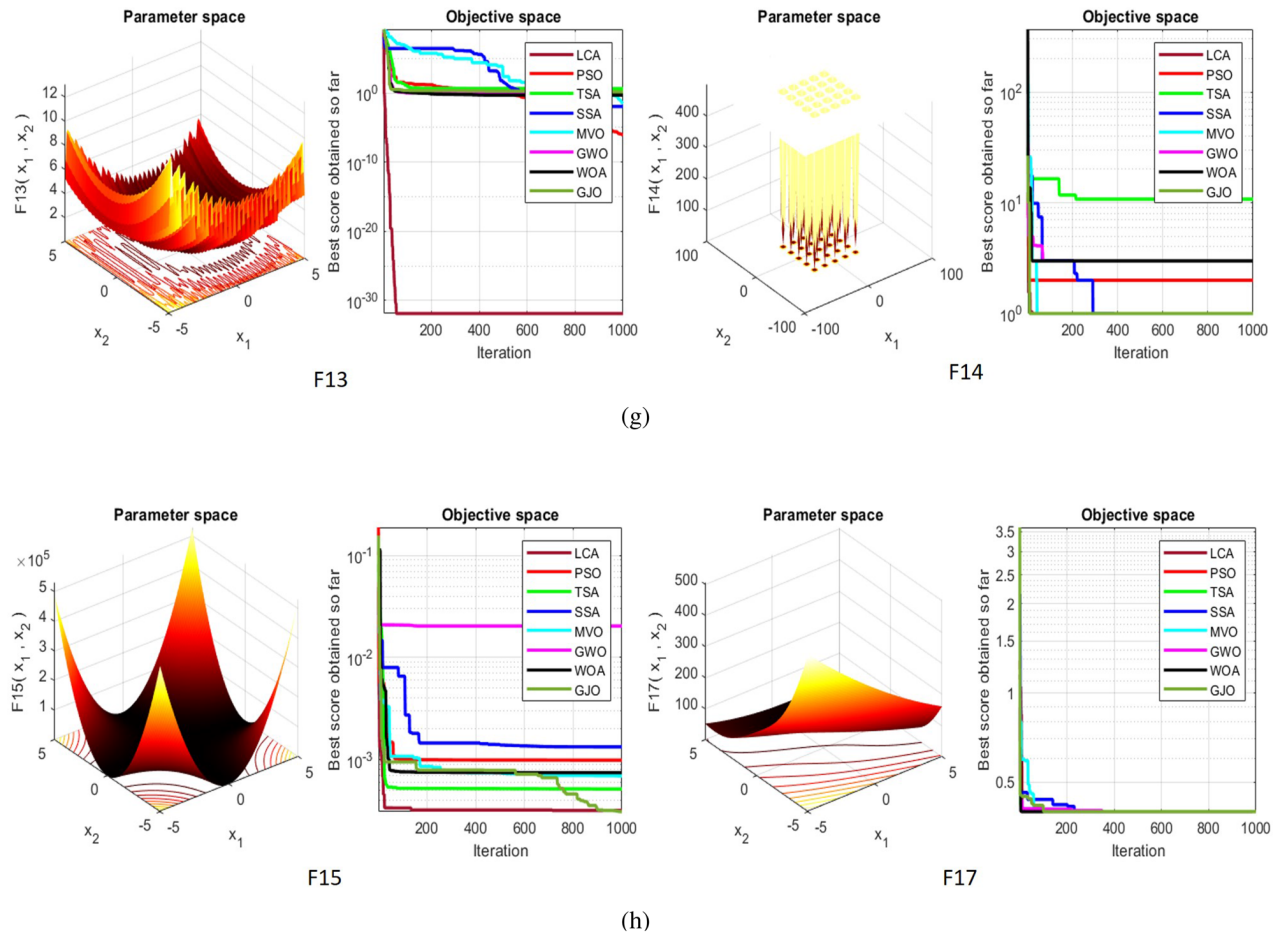

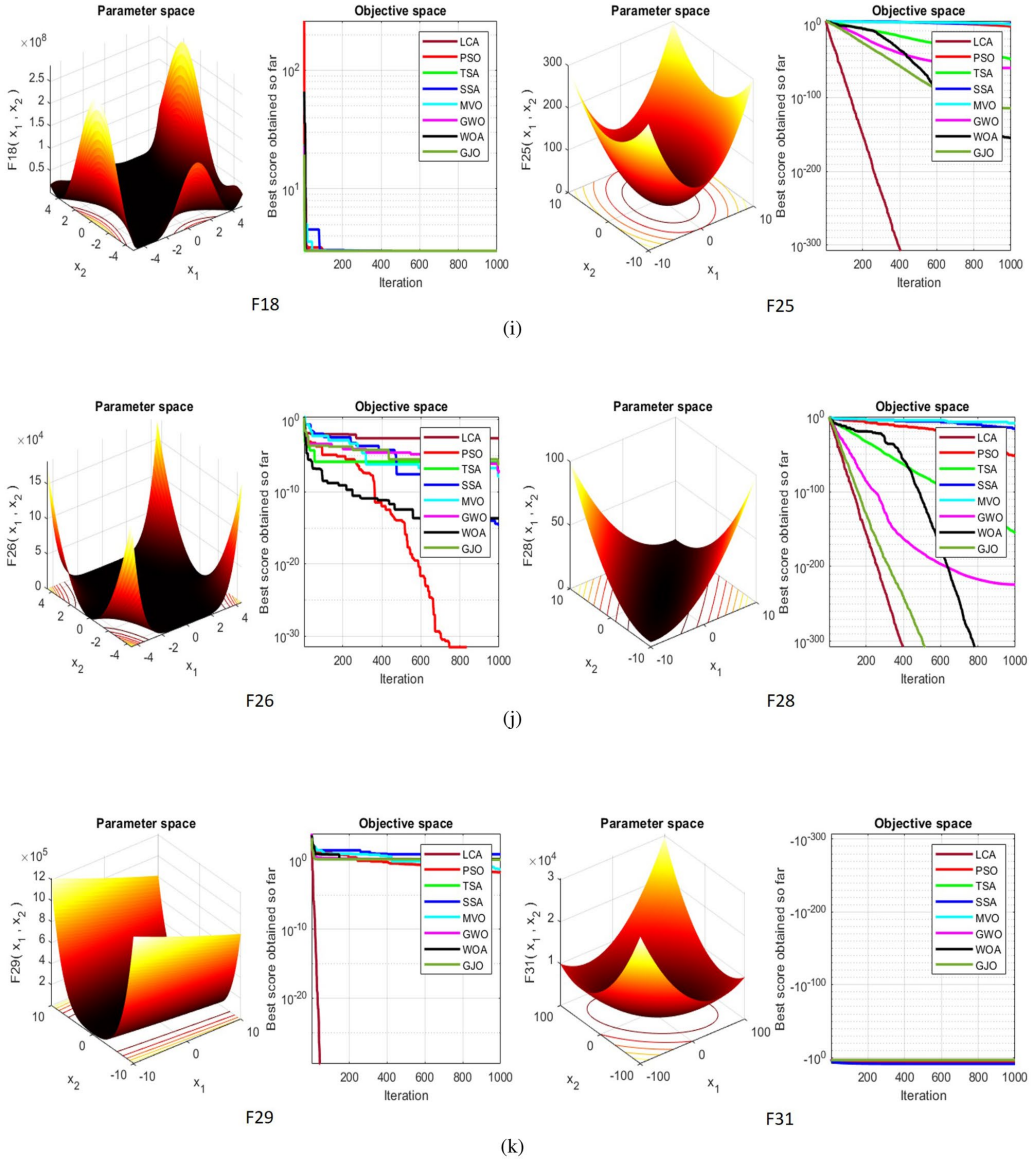

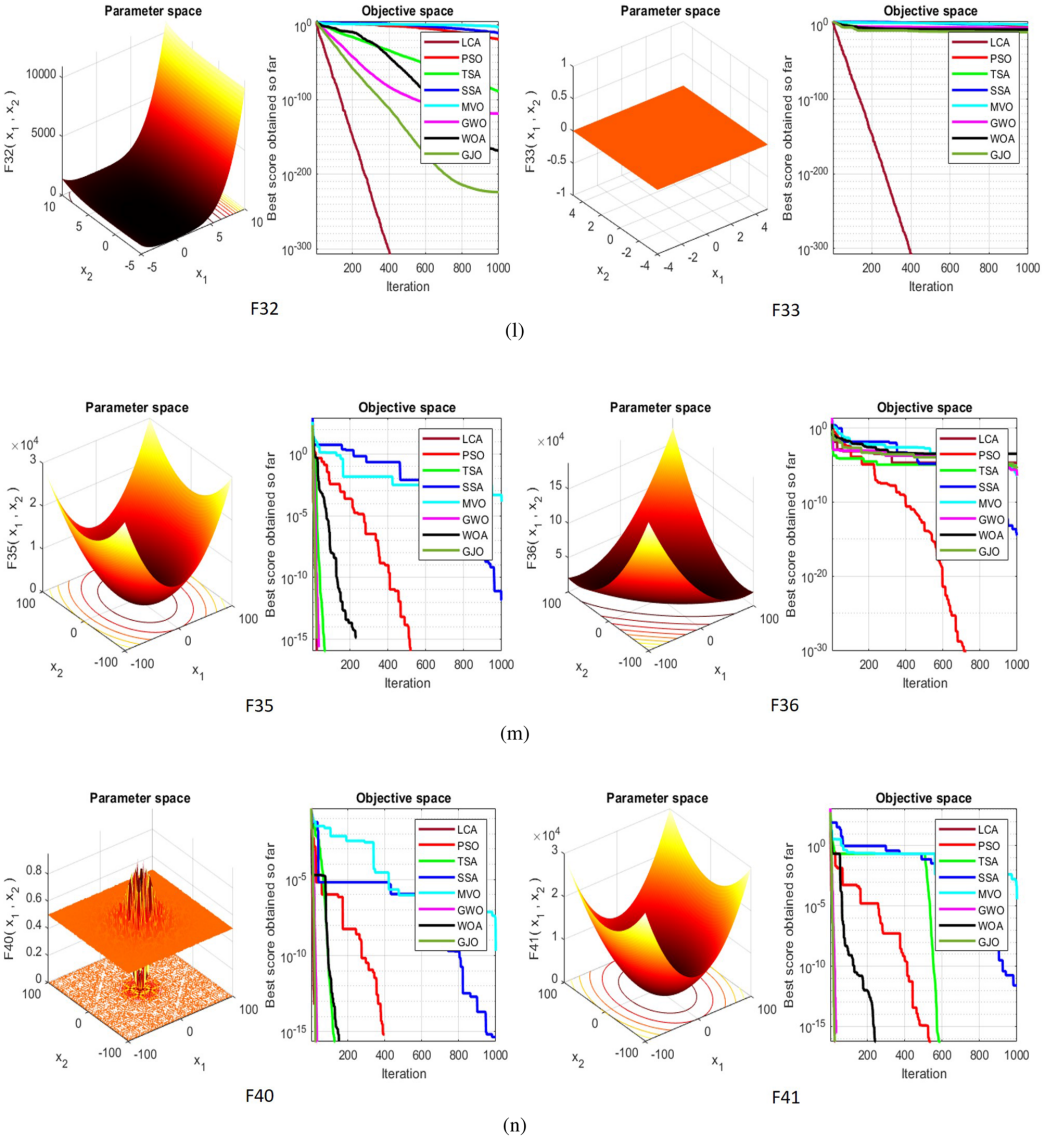

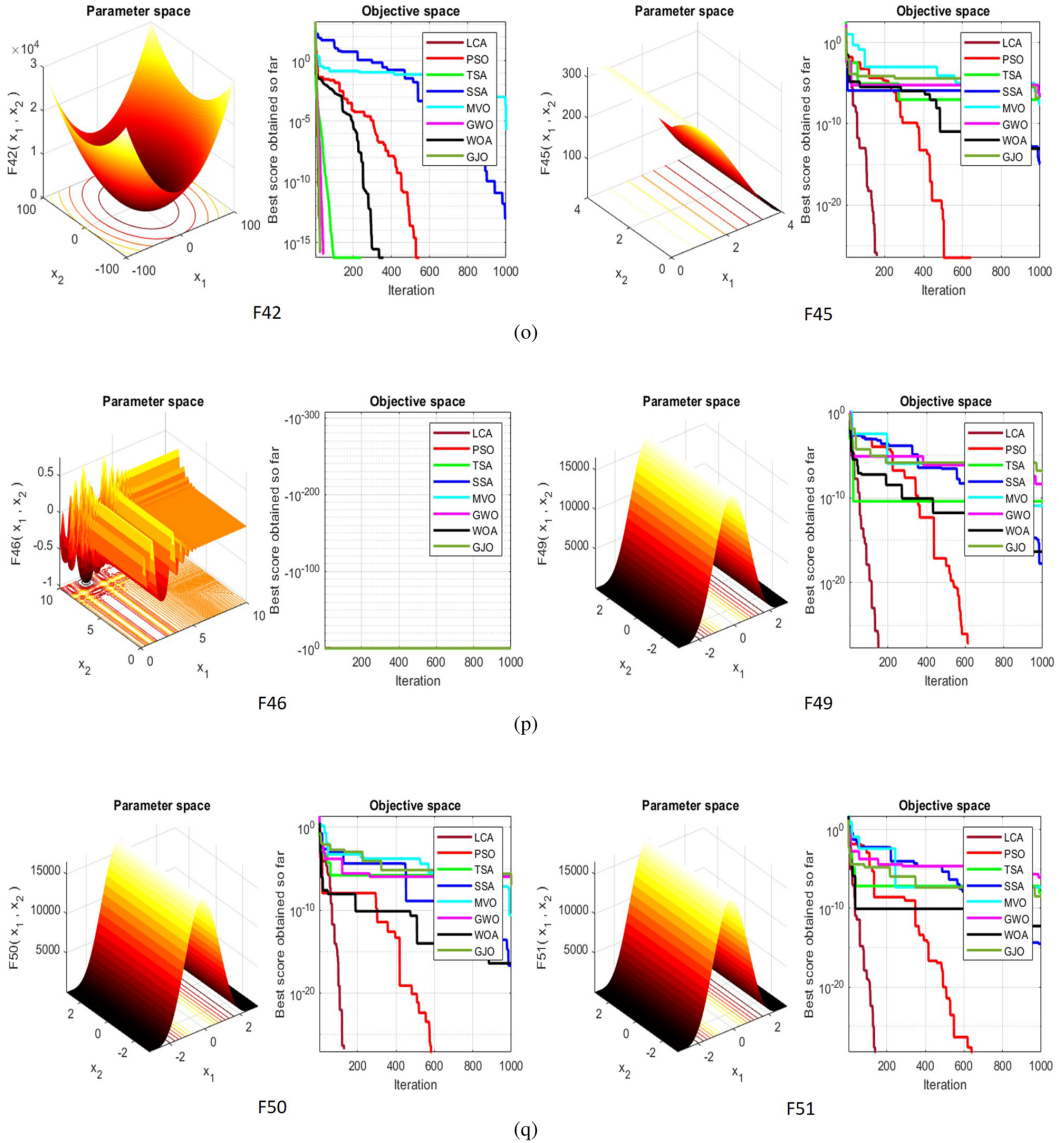
Convergence graph of LCA with well-known algorithms on the different benchmark functions.

**Figure 5:**
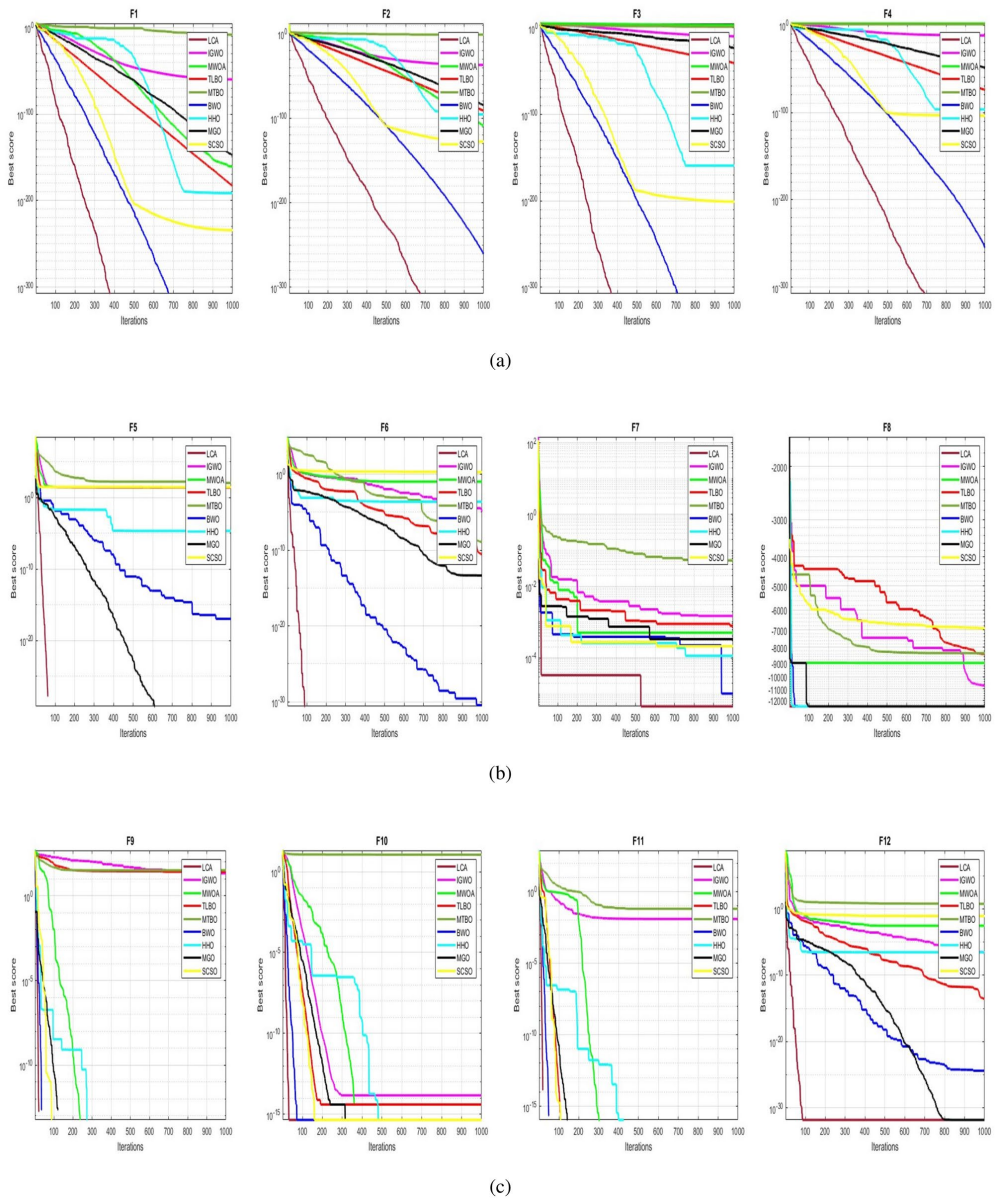

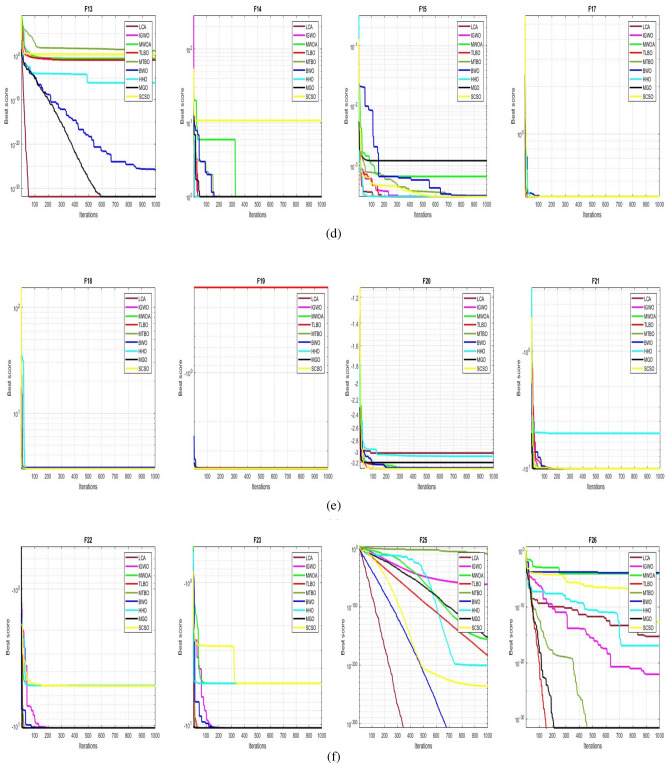

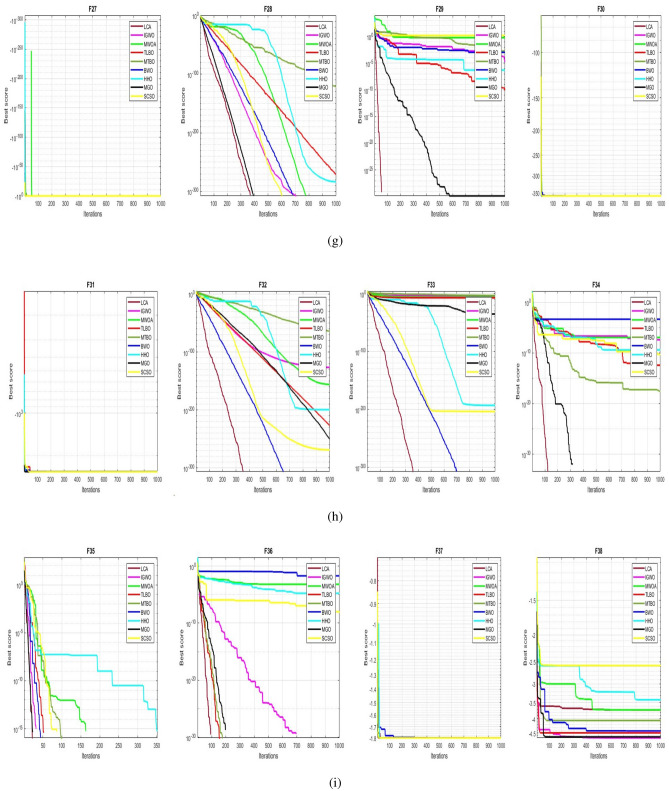

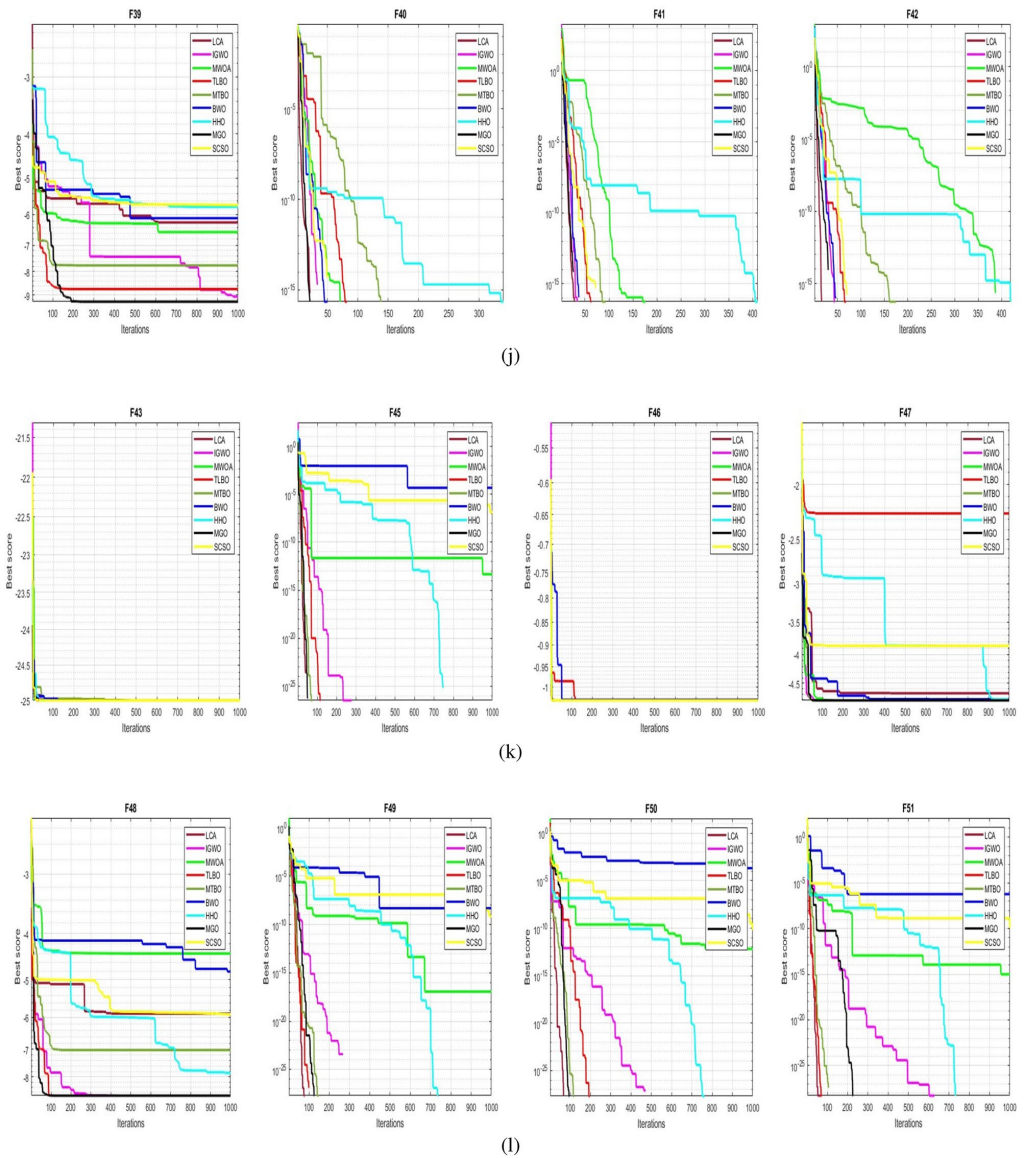
Convergence graph of LCA with recent algorithms on the different benchmark functions.

The convergence curve represents a relation between the fitness function value and the number of iterations. The search agent explores the search area and deviates rapidly in the beginning stage of the optimization process. The main objective behind the convergence analysis is to understand the behaviour and graphical representation of the proposed method. Figure 4 shows the convergence curves of LCA with well-known algorithms for different test functions. From Fig. 4 it is observed that the proposed method LCA converges faster among the benchmark functions except for *F*14, *F*17, *F*18, *F*26, and *F*36. Moreover, the LCA technique has a larger effect on the convergence of the other algorithms, especially compared with the PSO, TSA, SSA, MVO, GWO, WOA, and GJO. Figure 5 shows the convergence curves of LCA with recent algorithms for different test functions. From Fig. 5 it is observed that the proposed method LCA converges faster among the benchmark functions except for *F*14, $F17-F20$, *F*27, *F*30, *F*31, *F*37, *F*43, and *F*46. Moreover, the LCA technique has a larger effect on the convergence of the other algorithms, especially compared with the IGWO, MWOA, TLBO, MTBO, BWO, HHO, MGO, and SCSO. Thus, it is proven with the improvements that the proposed LCA can achieve a higher search accuracy and faster convergence.

**Table 11:** Evaluation results of well–known & top–performing algorithms for the CEC 2019 benchmark functions.

${\text {Functions}}$		${\text {PSO}}$	${\text {TSA}}$	${\text {SSA}}$	${\text {MVO}}$	${\text {GWO}}$	${\text {WOA}}$	${\text {GJO}}$	${\text {LSHADE}}$	${\text {CMAES}}$	${\text {LCA}}$
${\text {CEC19-1}}$	Mean	5.736E+11	1.291E+08	7.488E+09	2.351E+09	1.441E+08	2.685E+10	1.503E+07	1.445E+07	2.552E+08	8.214E+04
	Std	5.385E+11	4.965E+08	1.490E+10	1.522E+09	3.900E+08	3.348E+10	3.137E+07	2.981E+07	1.695E+08	1.500E+04
	Best	3.562E+10	4.417E+04	1.480E+08	6.789E+08	6.207E+04	1.177E+06	3.941E+04	8.727E+05	3.449E+07	5.556E+04
	Worst	2.678E+12	2.232E+09	6.778E+10	6.630E+09	1.363E+09	1.039E+11	1.237E+08	1.264E+08	7.858E+08	1.139E+05
	Median	5.077E+11	4.612E+05	3.122E+09	1.865E+09	6.839E+06	5.031E+09	6.357E+05	2.866E+06	2.206E+08	8.036E+04
${\text {CEC19-2}}$	Mean	1.040E+04	1.824E+01	1.734E+01	1.798E+01	1.734E+01	1.734E+01	1.741E+01	1.734E+01	1.098E+02	1.751E+01
	Std	2.253E+03	8.136E–01	9.461E–04	2.479E–01	1.835E–04	1.577E–03	1.387E–01	3.656E–14	5.120E+01	6.248E–02
	Best	7.586E+03	1.735E+01	1.734E+01	1.756E+01	1.734E+01	1.734E+01	1.734E+01	1.734E+01	4.401E+01	1.744E+01
	Worst	1.321E+04	1.956E+01	1.735E+01	1.826E+01	1.734E+01	1.735E+01	1.768E+01	1.734E+01	2.683E+02	1.759E+01
	Median	9.735E+03	1.797E+01	1.734E+01	1.797E+01	1.734E+01	1.734E+01	1.734E+01	1.734E+01	9.422E+01	1.753E+01
${\text {CEC19-3}}$	Mean	1.270E+01	1.270E+01	1.270E+01	1.270E+01	1.270E+01	1.270E+01	1.270E+01	1.270E+01	1.270E+01	1.270E+01
	Std	3.645E–15	2.124E–03	1.559E–03	3.645E–15	3.645E–15	3.645E–15	7.797E–04	5.468E–15	1.622E–04	3.645E–15
	Best	1.270E+01	1.270E+01	1.270E+01	1.270E+01	1.270E+01	1.270E+01	1.270E+01	1.270E+01	1.270E+01	1.270E+01
	Worst	1.270E+01	1.271E+01	1.271E+01	1.270E+01	1.270E+01	1.270E+01	1.270E+01	1.270E+01	1.270E+01	1.270E+01
	Median	1.270E+01	1.270E+01	1.270E+01	1.270E+01	1.270E+01	1.270E+01	1.270E+01	1.270E+01	1.270E+01	1.270E+01
${\text {CEC19-4}}$	Mean	1.393E+01	3.084E+03	4.895E+01	2.731E+01	6.104E+01	2.852E+02	5.413E+02	8.897E+00	2.365E+01	1.468E+04
	Std	4.134E+00	2.654E+03	2.256E+01	5.939E+00	1.310E+01	1.349E+02	6.189E+02	2.794E+00	4.062E+00	3.496E+03
	Best	8.955E+00	9.851E+01	2.985E+01	1.838E+01	4.738E+01	1.230E+02	4.559E+01	3.214E+00	1.319E+01	9.195E+03
	Worst	1.990E+01	7.697E+03	8.955E+01	3.501E+01	8.216E+01	4.492E+02	1.496E+03	1.393E+01	2.941E+01	1.948E+04
	Median	1.492E+01	2.499E+03	3.582E+01	2.705E+01	5.441E+01	3.454E+02	8.236E+01	8.180E+00	2.397E+01	1.570E+04
${\text {CEC19-5}}$	Mean	1.119E+00	2.412E+00	1.281E+00	1.262E+00	1.447E+00	1.737E+00	1.513E+00	1.033E+00	1.015E+00	4.441E+00
	Std	9.187E–02	6.133E–01	1.568E–01	5.654E–02	2.296E–01	4.503E–01	2.751E–01	2.789E–02	3.104E–02	5.932E–01
	Best	1.027E+00	1.773E+00	1.153E+00	1.193E+00	1.184E+00	1.368E+00	1.191E+00	1.000E+00	1.000E+00	3.312E+00
	Worst	1.268E+00	3.504E+00	1.580E+00	1.358E+00	1.794E+00	2.546E+00	1.819E+00	1.108E+00	1.076E+00	4.893E+00
	Median	1.108E+00	2.308E+00	1.229E+00	1.257E+00	1.404E+00	1.470E+00	1.574E+00	1.029E+00	1.000E+00	4.633E+00
${\text {CEC19-6}}$	Mean	6.249E+00	1.092E+01	4.311E+00	7.065E+00	1.042E+01	8.131E+00	1.029E+01	7.493E+00	1.040E+01	1.008E+01
	Std	1.487E+00	5.521E–01	1.416E+00	9.508E–01	5.330E–01	8.608E–01	3.207E–01	1.288E+00	5.982E–01	6.086E–01
	Best	4.540E+00	1.037E+01	1.709E+00	6.090E+00	1.000E+01	6.715E+00	9.879E+00	4.781E+00	9.467E+00	9.146E+00
	Worst	8.356E+00	1.186E+01	5.361E+00	8.620E+00	1.130E+01	9.189E+00	1.067E+01	1.141E+01	1.167E+01	1.091E+01
	Median	6.605E+00	1.067E+01	5.192E+00	6.687E+00	1.004E+01	8.399E+00	1.014E+01	7.406E+00	1.032E+01	1.025E+01
${\text {CEC19-7}}$	Mean	1.447E+02	6.204E+02	3.097E+02	2.353E+02	1.666E+02	8.144E+02	7.109E+02	–5.339E+01	3.170E+02	7.229E+02
	Std	1.620E+02	2.291E+02	2.098E+02	1.242E+02	2.100E+02	3.491E+02	2.902E+02	8.355E+01	2.543E+02	1.282E+02
	Best	–3.788E+01	2.955E+02	3.335E+01	8.290E+01	–8.679E+01	4.574E+02	3.776E+02	–1.695E+02	6.247E–01	5.753E+02
	Worst	4.154E+02	9.176E+02	6.048E+02	4.319E+02	4.484E+02	1.290E+03	1.068E+03	6.334E+01	7.868E+02	8.552E+02
	Median	1.236E+02	5.598E+02	2.810E+02	2.381E+02	1.462E+02	7.154E+02	7.882E+02	–6.668E+01	3.193E+02	7.495E+02
${\text {CEC19-8}}$	Mean	4.831E+00	6.588E+00	5.091E+00	4.381E+00	4.926E+00	5.817E+00	4.802E+00	4.642E+00	5.129E+00	6.144E+00
	Std	8.981E–01	2.275E–01	5.419E–01	9.540E–01	5.200E–01	9.091E–01	1.018E+00	5.651E–01	1.620E+00	3.161E–01
	Best	3.671E+00	6.273E+00	4.427E+00	3.161E+00	4.233E+00	4.200E+00	3.663E+00	3.247E+00	1.700E+00	5.722E+00
	Worst	6.197E+00	6.921E+00	5.726E+00	5.842E+00	5.586E+00	6.642E+00	6.155E+00	5.535E+00	6.968E+00	6.486E+00
	Median	4.895E+00	6.650E+00	5.380E+00	4.212E+00	4.944E+00	6.120E+00	4.330E+00	4.731E+00	5.717E+00	6.150E+00
${\text {CEC19-9}}$	Mean	2.357E+00	6.082E+01	2.450E+00	2.383E+00	3.967E+00	4.379E+00	5.036E+00	2.451E+00	2.393E+00	2.310E+03
	Std	1.227E–02	1.135E+02	4.750E–02	9.900E–03	8.216E–01	4.976E–01	5.327E–01	1.000E–01	1.657E–02	3.663E+02
	Best	2.344E+00	3.780E+00	2.376E+00	2.372E+00	3.038E+00	3.877E+00	4.386E+00	2.356E+00	2.365E+00	1.758E+03
	Worst	2.372E+00	2.821E+02	2.506E+00	2.401E+00	4.958E+00	5.224E+00	5.576E+00	2.807E+00	2.439E+00	2.694E+03
	Median	2.354E+00	6.005E+00	2.464E+00	2.380E+00	3.944E+00	4.239E+00	5.249E+00	2.426E+00	2.393E+00	2.351E+03
${\text {CEC19-10}}$	Mean	2.006E+01	2.047E+01	2.000E+01	2.005E+01	2.047E+01	2.018E+01	2.046E+01	2.009E+01	2.042E+01	2.048E+01
	Std	5.323E–02	7.487E–02	4.104E–05	3.456E–02	5.762E–02	5.076E–02	7.551E–02	8.036E–02	9.261E–02	8.779E–02
	Best	2.001E+01	2.038E+01	2.000E+01	2.001E+01	2.035E+01	2.014E+01	2.034E+01	2.000E+01	2.025E+01	2.037E+01
	Worst	2.014E+01	2.060E+01	2.000E+01	2.011E+01	2.050E+01	2.027E+01	2.054E+01	2.040E+01	2.057E+01	2.060E+01
	Median	2.004E+01	2.044E+01	2.000E+01	2.004E+01	2.048E+01	2.015E+01	2.044E+01	2.007E+01	2.043E+01	2.047E+01

### Comparison results of LCA with well-known & top-performing algorithms for CEC 2019 test functions

In this subsection, we discuss the efficiency of the proposed LCA and compare it with well-known algorithms and top-performing algorithms. Table 11 demonstrates the evaluation results of competitive algorithms. In comparison to PSO, LCA produces better results in 2 functions, such as CEC19-1, and CEC19-2 . In comparison to TSA, LCA produces better results in 5 functions, such as CEC19-1, CEC19-2, CEC19-3, CEC19-6, and CEC19-8. In comparison to SSA, LCA produces better results in 2 functions, such as CEC19-1 and CEC19-3. In comparison to MVO, LCA produces better results in 2 functions, such as CEC19-1 and CEC19-2. In comparison to GWO, LCA produces better results in 2 functions, such as CEC19-1 and CEC19-6. In comparison to WOA, LCA produces better results in 2 functions, such as CEC19-1 and CEC19-7. In comparison to GJO, LCA produces better results in 3 functions, such as CEC19-1, CEC19-3, and CEC19-6. In comparison to LSHADE, LCA produces better results in 2 functions, such as CEC19-1 and CEC19-3. In comparison to CMAES, LCA produces better results in 4 functions, such as CEC19-1, CEC19-2, CEC19-3, and CEC19-6. Hence, the proposed LCA algorithm has a good exploitation ability and a good spatial exploration ability, which makes it possible for it to handle optimization problems successfully.

**Table 12:** Evaluation results of recent algorithms for the CEC 2019 benchmark functions.

${\text {Functions}}$		${\text {IGWO}}$	${\text {MWOA}}$	${\text {TLBO}}$	${\text {MTBO}}$	${\text {BWO}}$	${\text {HHO}}$	${\text {MGO}}$	${\text {SCSO}}$	${\text {LCA}}$
${\text {CEC19-1}}$	Mean	3.168E+07	7.839E+09	6.080E+07	2.000E+08	5.874E+04	4.815E+04	4.304E+04	4.335E+04	8.214E+04
	Std	3.243E+07	1.054E+10	7.794E+07	2.511E+08	5.625E+03	3.019E+03	3.108E+03	2.426E+03	1.500E+04
	Best	3.121E+05	1.332E+06	5.452E+04	4.406E+06	4.777E+04	4.475E+04	3.879E+04	3.932E+04	5.556E+04
	Worst	1.110E+08	3.010E+10	3.485E+08	1.108E+09	6.847E+04	5.563E+04	4.921E+04	4.977E+04	1.139E+05
	Median	2.064E+07	2.452E+09	3.873E+07	1.127E+08	5.814E+04	4.731E+04	4.298E+04	4.294E+04	8.036E+04
${\text {CEC19-2}}$	Mean	1.734E+01	1.735E+01	1.734E+01	1.734E+01	1.750E+01	1.736E+01	1.734E+01	1.739E+01	1.751E+01
	Std	1.684E–11	1.730E–03	0	0	4.670E–02	9.001E–03	0	1.227E–01	6.248E–02
	Best	1.734E+01	1.734E+01	1.734E+01	1.734E+01	1.744E+01	1.734E+01	1.734E+01	1.734E+01	1.744E+01
	Worst	1.734E+01	1.735E+01	1.734E+01	1.734E+01	1.759E+01	1.738E+01	1.734E+01	1.769E+01	1.759E+01
	Median	1.734E+01	1.734E+01	1.734E+01	1.734E+01	1.750E+01	1.736E+01	1.734E+01	1.734E+01	1.753E+01
${\text {CEC19-3}}$	Mean	1.270E+01	1.270E+01	1.270E+01	1.270E+01	1.270E+01	1.270E+01	1.270E+01	1.270E+01	1.270E+01
	Std	3.868E–09	8.406E–08	5.468E–15	4.558E–14	1.139E–04	3.072E–06	2.474E–10	9.152E–06	3.645E–15
	Best	1.270E+01	1.270E+01	1.270E+01	1.270E+01	1.270E+01	1.270E+01	1.270E+01	1.270E+01	1.270E+01
	Worst	1.270E+01	1.270E+01	1.270E+01	1.270E+01	1.270E+01	1.270E+01	1.270E+01	1.270E+01	1.270E+01
	Median	1.270E+01	1.270E+01	1.270E+01	1.270E+01	1.270E+01	1.270E+01	1.270E+01	1.270E+01	1.270E+01
${\text {CEC19-4}}$	Mean	3.339E+01	2.491E+02	4.138E+01	3.582E+01	7.046E+03	1.350E+02	3.040E+01	5.662E+02	1.468E+04
	Std	6.460E+00	1.294E+02	1.677E+01	1.859E+01	2.061E+03	4.801E+01	1.312E+01	9.662E+02	3.496E+03
	Best	1.924E+01	1.121E+02	1.691E+01	1.691E+01	2.758E+03	5.326E+01	7.960E+00	3.134E+01	9.195E+03
	Worst	4.731E+01	6.172E+02	8.457E+01	8.557E+01	1.110E+04	2.206E+02	5.472E+01	4.106E+03	1.948E+04
	Median	3.291E+01	2.054E+02	3.681E+01	3.234E+01	7.184E+03	1.443E+02	2.587E+01	1.214E+02	1.570E+04
${\text {CEC19-5}}$	Mean	1.371E+00	1.637E+00	1.114E+00	1.158E+00	3.527E+00	2.383E+00	1.144E+00	1.378E+00	4.441E+00
	Std	1.958E–01	3.398E–01	6.334E–02	1.079E–01	4.190E–01	4.883E–01	1.104E–01	2.504E–01	5.932E–01
	Best	1.016E+00	1.214E+00	1.031E+00	1.022E+00	2.210E+00	1.473E+00	1.027E+00	1.117E+00	3.312E+00
	Worst	1.676E+00	2.572E+00	1.295E+00	1.396E+00	4.005E+00	3.305E+00	1.436E+00	2.300E+00	4.893E+00
	Median	1.428E+00	1.561E+00	1.095E+00	1.128E+00	3.594E+00	2.357E+00	1.119E+00	1.335E+00	4.633E+00
${\text {CEC19-6}}$	Mean	1.034E+01	8.822E+00	1.027E+01	1.042E+01	1.046E+01	8.530E+00	8.938E+00	6.929E+00	1.008E+01
	Std	6.420E–01	1.512E+00	4.960E–01	7.811E–01	8.659E–01	1.274E+00	1.671E+00	1.378E+00	6.086E–01
	Best	8.415E+00	5.916E+00	9.231E+00	8.836E+00	8.449E+00	6.227E+00	5.059E+00	4.741E+00	9.146E+00
	Worst	1.127E+01	1.103E+01	1.106E+01	1.167E+01	1.164E+01	1.046E+01	1.048E+01	9.907E+00	1.091E+01
	Median	1.044E+01	8.647E+00	1.029E+01	1.044E+01	1.070E+01	8.795E+00	9.313E+00	7.054E+00	1.025E+01
${\text {CEC19-7}}$	Mean	4.356E+02	4.339E+02	4.764E+02	5.391E+02	8.756E+02	3.630E+02	1.190E+02	4.187E+02	7.229E+02
	Std	1.623E+02	2.091E+02	1.758E+02	2.465E+02	1.219E+02	1.561E+02	1.998E+02	2.255E+02	1.282E+02
	Best	3.336E+01	–6.536E+00	9.752E+01	–5.586E+01	6.337E+02	5.773E+01	–2.273E+02	8.903E+01	5.753E+02
	Worst	6.270E+02	8.335E+02	7.403E+02	1.068E+03	1.078E+03	6.236E+02	4.620E+02	8.580E+02	8.552E+02
	Median	4.950E+02	4.506E+02	5.121E+02	5.738E+02	8.918E+02	3.716E+02	1.157E+02	4.388E+02	7.495E+02
${\text {CEC19-8}}$	Mean	2.807E+00	5.961E+00	3.722E+00	5.032E+00	6.346E+00	5.657E+00	3.973E+00	5.217E+00	6.144E+00
	Std	9.412E–01	4.483E–01	9.623E–01	8.083E–01	1.942E–01	6.645E–01	1.099E+00	7.103E–01	3.161E–01
	Best	1.357E+00	5.119E+00	2.461E+00	3.554E+00	5.952E+00	4.521E+00	2.061E+00	3.305E+00	5.722E+00
	Worst	5.240E+00	6.821E+00	5.157E+00	6.375E+00	6.753E+00	6.716E+00	5.437E+00	6.279E+00	6.486E+00
	Median	2.792E+00	5.916E+00	3.674E+00	4.971E+00	6.330E+00	5.864E+00	4.039E+00	5.354E+00	6.150E+00
${\text {CEC19-9}}$	Mean	2.378E+00	4.464E+00	2.343E+00	2.371E+00	1.330E+03	2.775E+00	2.384E+00	4.449E+00	2.310E+03
	Std	1.464E–02	8.972E–01	3.298E–03	4.333E–02	3.853E+02	2.262E–01	3.096E–02	1.174E+00	3.663E+02
	Best	2.345E+00	3.297E+00	2.339E+00	2.341E+00	7.510E+02	2.504E+00	2.351E+00	2.685E+00	1.758E+03
	Worst	2.411E+00	6.094E+00	2.350E+00	2.518E+00	2.083E+03	3.401E+00	2.444E+00	6.547E+00	2.694E+03
	Median	2.376E+00	4.364E+00	2.342E+00	2.352E+00	1.365E+03	2.739E+00	2.370E+00	4.151E+00	2.351E+03
${\text {CEC19-10}}$	Mean	1.835E+01	2.018E+01	1.862E+01	2.042E+01	2.047E+01	2.015E+01	1.839E+01	2.011E+01	2.048E+01
	Std	6.276E+00	1.232E–01	5.241E+00	6.169E–02	1.015E–01	1.185E–01	5.826E+00	9.775E–02	8.779E–02
	Best	1.852E–03	2.004E+01	1.646E+00	2.031E+01	2.013E+01	2.002E+01	1.552E–05	1.999E+01	2.037E+01
	Worst	2.054E+01	2.057E+01	2.048E+01	2.052E+01	2.058E+01	2.042E+01	2.042E+01	2.040E+01	2.060E+01
	Median	2.038E+01	2.017E+01	2.041E+01	2.042E+01	2.048E+01	2.012E+01	2.026E+01	2.009E+01	2.047E+01

### Comparison results of LCA with recent algorithms for CEC 2019 test functions

In this subsection, we discuss the efficiency of the proposed LCA and compare it with recent algorithms. Table 12 demonstrates the evaluation results of competitive algorithms. In comparison to IGWO, LCA produces better results in 3 functions, such as CEC19-1, CEC19-3, and CEC19-6 . In comparison to MGWO, LCA produces better results in 2 functions, such as CEC19-1 and CEC19-3. In comparison to TLBO, LCA produces better results in 3 functions, such as CEC19-1, CEC19-3, and CEC19-6. In comparison to MTBO, LCA produces better results in 3 functions, such as CEC19-1, CEC19-3, and CEC19-6. In comparison to BWO, LCA produces better results in 5 functions, such as CEC19-2, CEC19-3, CEC19-6, CEC19-7, and CEC19-8. In comparison to HHO, LCA produces better results in 1 function, such as CEC19-3. In comparison to MGO, LCA produces better results in 1 function, such as CEC19-3. In comparison to SCSO, LCA produces better results in 1 function, such as CEC19-3. Hence, the proposed LCA algorithm has a good exploitation ability and a good spatial exploration ability, which makes it possible for it to handle optimization problems successfully.

### Convergence analysis for CEC 2019 benchmark functions

**Figure 6:**
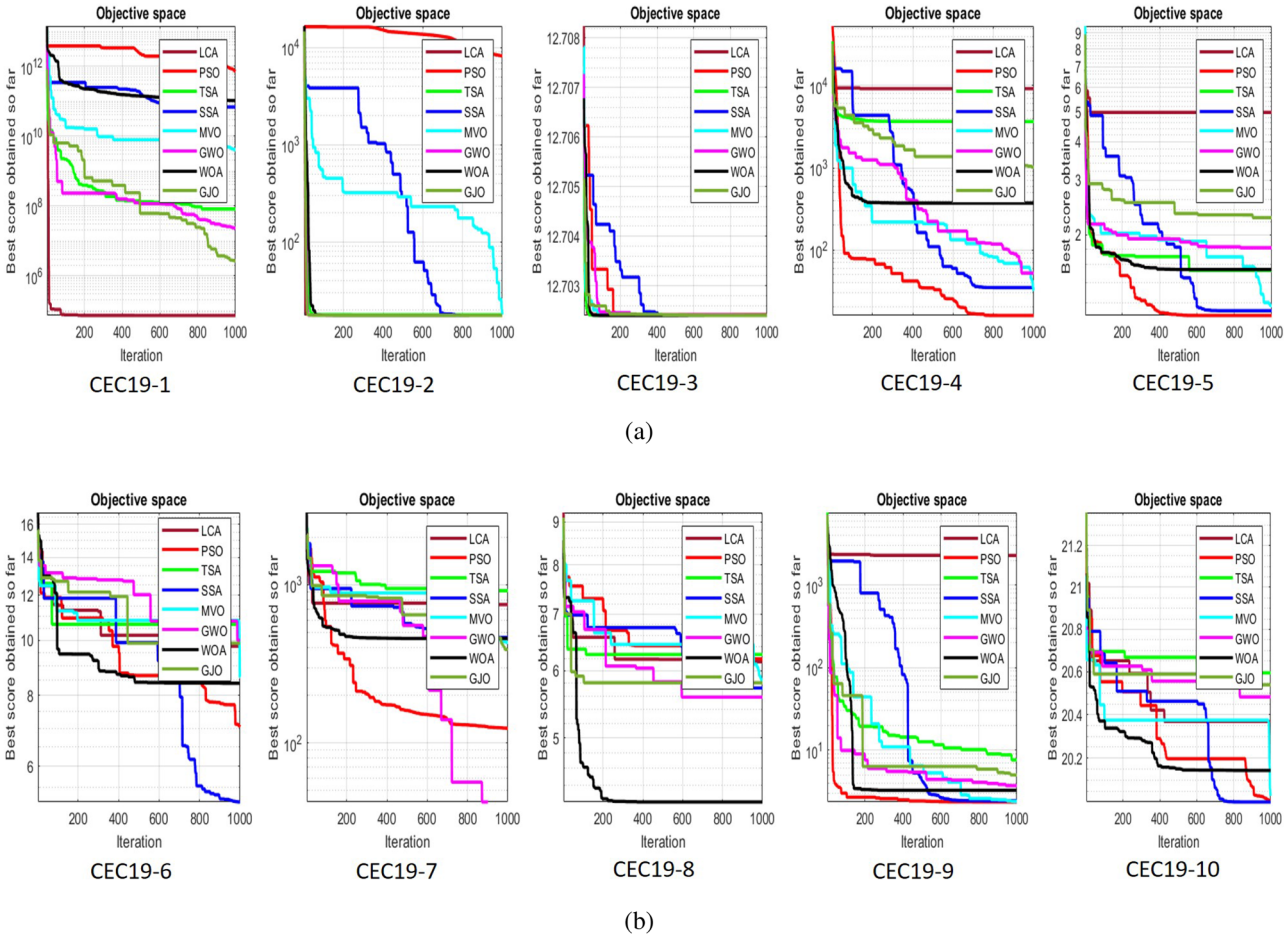
Convergence graph of LCA with well-known algorithms on the CEC 2019 benchmark functions.

**Figure 7:**
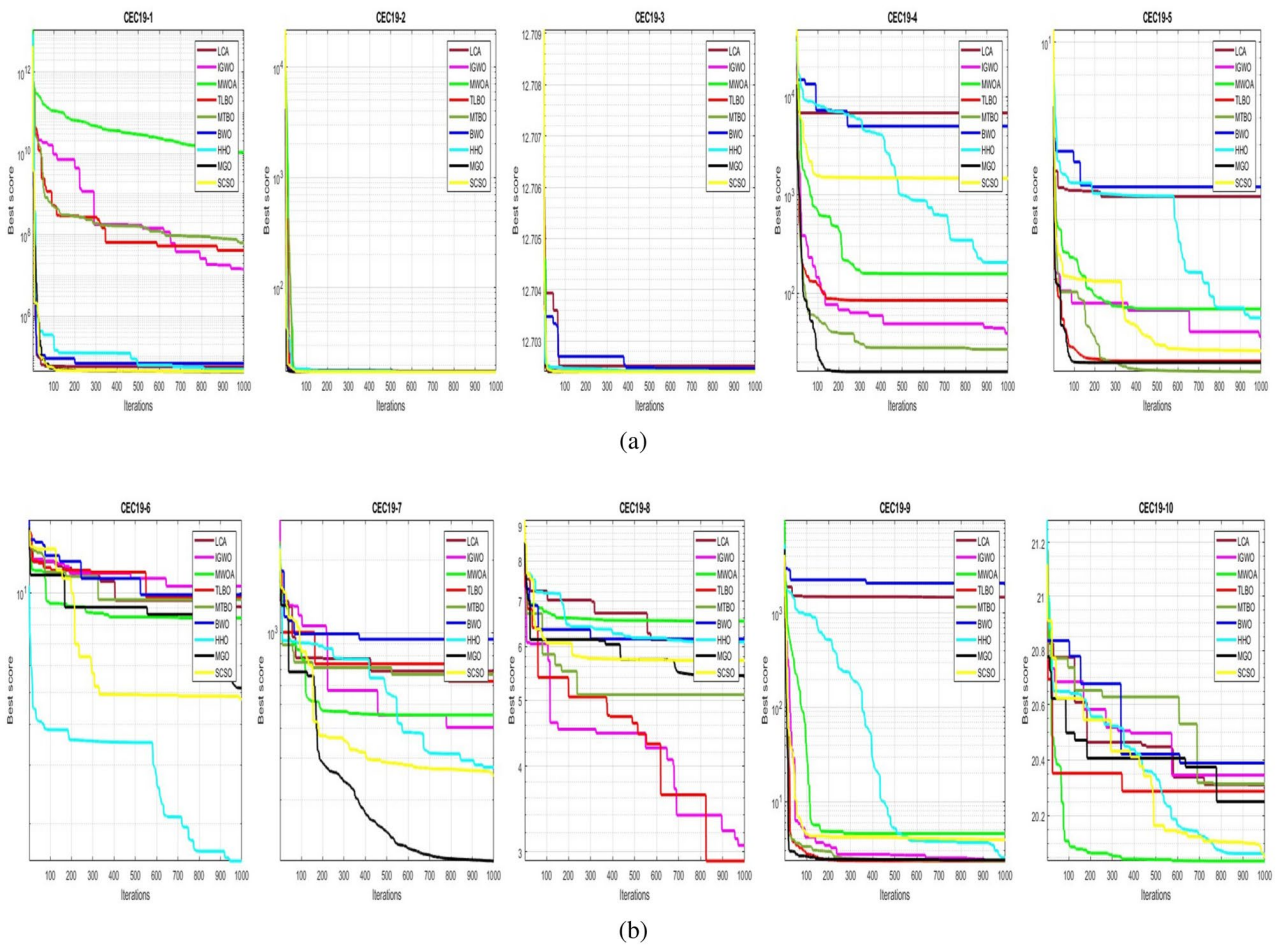
Convergence graph of LCA with recent algorithms on the CEC 2019 benchmark functions.

The main objective behind the convergence analysis is to understand the behaviour and graphical representation of the proposed method. Figure 6 shows the convergence curves of LCA with well-known algorithms for CEC 2019 benchmark functions. From the Fig. 6, it is observed that the proposed method LCA converges faster among CEC 2019 benchmark functions except for $CEC19-2$, $CEC19-3$, $CEC19-4$, $CEC19-5$, $CEC19-5$, $CEC19-5$, $CEC19-6$, $CEC19-7$, $CEC19-8$, $CEC19-9$, and $CEC19-19$. Moreover, the LCA technique has a larger effect on the convergence of the other algorithms, especially compared with the PSO, TSA, SSA, MVO, GWO, WOA, and GJO. Figure 7 shows the convergence curves of LCA with recent algorithms for CEC 2019 benchmark functions. From the Fig. 7 it is observed that the proposed method LCA converges faster among CEC 2019 benchmark functions except for $CEC19-2$, $CEC19-3$, $CEC19-4$, $CEC19-5$, $CEC19-5$, $CEC19-5$, $CEC19-6$, $CEC19-7$, $CEC19-8$, $CEC19-9$, and $CEC19-19$. Moreover, the LCA technique has a larger effect on the convergence of the other algorithms, especially compared with the IGWO, MWOA, TLBO, MTBO, BWO, HHO, MGO, and SCSO.

### Statistical analysis

The Wilcoxon rank-sum test^95^ is used to provide statistical analysis of LCA performance in comparison to competing algorithms. Based on a statistic known as the *p* value, The Wilcoxon rank-sum test evaluates if the superiority of one approach over another is statistically significant. The results of performing the Wilcoxon rank-sum test to LCA are compared to each of the competing algorithms. The results show that LCA is statistically better than a similar competitor algorithm in any case where the *p* value is estimated to be less than 0.05. The symbol *P* denotes the hypothesis. Two-tailed *t*-tests have been used to compare different statistical results at a significance level of 0.05. The *t* values are provided with the help of mean values and standard deviations. A negative *t* value indicates that the statistical outcomes of the LCA optimization errors are significantly less, and vice versa. The corresponding *t* value is highlighted if the difference is a statistically significant error. The symbols *w*/*t*/*l* denote that LCA wins in *w* functions, ties in *t* functions, and loses in *l* functions. Table 13 shows the Wilcoxon rank-sum test and t-test results for LCA versus well-known and top-performing algorithms for 51 benchmark functions. Table 14 shows the Wilcoxon rank-sum test and t-test results for LCA versus recent algorithms for 51 benchmark functions. Table 15 shows the Wilcoxon rank-sum test and t-test results for LCA versus well-known and top-performing algorithms for CEC 2019 benchmark functions. Table 16 shows the Wilcoxon rank-sum test and t-test results for LCA versus recent algorithms for CEC 2019 benchmark functions. Table 17 shows the average run time of algorithms for the 23 benchmark functions. Table 18 shows the Wilcoxon rank-sum test and t-test validation for LCA versus well-known and top-performing algorithms for 51 benchmark functions. Table 19 shows the Wilcoxon rank-sum test and *t*-test validation for LCA versus recent algorithms for 51 benchmark functions. Table 20 shows the Wilcoxon rank-sum test and *t*-test validation for LCA versus well-known and top-performing algorithms for CEC 2019 benchmark functions. Table 21 shows the Wilcoxon rank-sum test and *t*-test validation for LCA versus recent algorithms for CEC 2019 benchmark functions. The statistical results of the optimization errors demonstrate that LCA has a much superior overall performance when compared with the other algorithms.

**Table 13:** Wilcoxon rank–sum test and t-test results for LCA versus well–known and top-performing algorithms for 51 benchmark functions.

Functions		LCA vs	LCA vs	LCA vs	LCA vs	LCA vs	LCA vs	LCA vs	LCA vs	LCA vs
		PSO	TSA	SSA	MVO	GWO	WOA	GJO	LSHADE	CMAES
${\text {F1}}$	$p-value$	9.119E–05	9.110E–05	9.110E–05	9.110E–05	9.110E–05	9.110E–05	9.110E–05	9.190E–05	9.120E–05
	$t-test$	–3.935468	–2.185655	–20.00023	–15.98234	–2.6901698	0	–1.805788	–3.915415	–4.914035
${\text {F2}}$	$p-value$	9.110E–05	9.110E–05	9.110E–05	9.110E–05	9.110E–05	9.110E–05	9.110E–05	9.140E–05	9.130E–05
	$t-test$	–8.304950	–2.3503565	–3.656791	–13.62703	–5.797903	–1.462105	–3.059250	–2.431003	–11.34986
${\text {F3}}$	$p-value$	9.110E–05	9.110E–05	9.110E–05	9.110E–05	9.110E–05	9.110E–05	9.110E–05	9.160E–05	9.090E–05
	$t-test$	–9.509E+00	–2.192E+00	–5.670E+00	–1.043E+01	–1.481E+00	–8.789E+00	–1.501E+00	–5.716330	–6.599026
${\text {F4}}$	$p-value$	9.110E–05	9.101E–05	9.101E–05	9.100E–05	9.110E–05	9.101E–05	9.110E–05	9.160E–05	9.110E–05
	$t-test$	–1.979E+01	–2.090E+00	–9.698E+00	–1.069E+01	–6.560E+00	–5.699E+00	–1.646E+00	–17.18328	–15.38482
${\text {F5}}$	$p-value$	9.110E–05	9.110E–05	9.120E–05	9.100E–05	9.101E–05	9.110E–05	9.120E–05	9.120E–05	9.140E–05
	$t-test$	–5.222E+00	–1.546E+02	–2.351E+00	–3.373E+00	–1.991E+02	–1.634E+02	–1.628E+02	–7.078189	–19.44548
${\text {F6}}$	$p-value$	9.110E–05	9.110E–05	9.100E–05	9.110E–05	9.120E–05	9.110E–05	9.110E–05	9.170E–05	9.130E–05
	$t-test$	–7.445E+00	–3.806E+01	–1.059E+01	–1.637E+01	–1.144E+01	–3.256E+00	–2.339E+01	–1.511763	–5.993604
${\text {F7}}$	$p-value$	9.110E–05	9.110E–05	9.120E–05	9.110E–05	9.110E–05	9.110E–05	9.120E–05	9.210E–05	9.210E–05
	$t-test$	–2.011E+01	–1.487E+01	–1.073E+01	–1.666E+01	–9.593E+00	–4.683E+00	–9.720E+00	–10.48742	–9.983163
${\text {F8}}$	$p-value$	9.10E–05	9.10E–05	9.10E–05	9.10E–05	9.10E–05	9.10E–05	9.10E–05	9.63E–05	9.12E–05
	$t-test$	–2.752E+01	–3.992E+01	1.833E+01	–6.688E+01	–5.972E+01	–3.539E+00	–3.386E+01	–7.709736	0
${\text {F9}}$	$p-value$	9.120E–05	9.110E–05	9.130E–05	9.110E–05	*NA*	*NA*	*NA*	9.160E–05	9.130E–05
	$t-test$	–24.34744	–20.97211	–22.28598	–13.95689	–1.452966	0	0	–1.001548	–96.23406
${\text {F10}}$	$p-value$	9.110E–05	8.701E–05	9.120E–05	9.110E–05	3.101E–05	1.220E–04	8.110E–06	9.170E–05	9.140E–05
	$t-test$	–1.566E+00	–5.276E+00	–7.926E+00	–8.549E+00	–2.397E+01	–5.811E+00	–6.520E+15	–20.25979	–7.952658
${\text {F11}}$	$p-value$	9.10E–05	*NA*	9.10E–05	9.10E–05	*NA*	*NA*	*NA*	9.18E–05	*NA*
	$t-test$	–7.880752	–4.248139	–6.080502	–25.43402	–1.452966	0	0	–3.348001	0
${\text {F12}}$	$p-value$	9.110E–05	9.120E–05	9.120E–05	9.130E–05	9.110E–05	9.120E–05	9.110E–05	9.160E–05	9.131E–05
	$t-test$	–6.544E+00	–1.282E+01	–8.636E+00	–7.885E+00	–8.596E+00	–5.554E+00	–1.318E+01	–1.836609	–1.907916
${\text {F13}}$	$p-value$	9.112E–05	9.111E–05	9.101E–05	9.103E–05	9.110E–05	9.100E–05	9.104E–05	9.170E–05	9.160E–05
	$t-test$	–2.184E+00	–1.924E+01	–2.716E+00	–8.278E+00	–1.117E+01	–7.612E+00	–4.083E+01	–1.270109	–3.599126
${\text {F14}}$	$p-value$	0.000185	1.935E–04	*NA*	*NA*	*NA*	*NA*	3.836E–04	8.140E–06	9.140E–05
	$t-test$	–4.885E+00	–5.617E+00	–1.452E+00	0	–3.078E+00	–4.358E+00	–6.120E+00	–5889555	–5.871148
${\text {F15}}$	$p-value$	9.210E–05	1.761E–03	9.210E–05	9.110E–05	3.176E–01	2.616E–04	8.753E–02	1.340E–02	9.190E–05
	$t-test$	–8.627E+00	–2.371E+00	–1.197E+01	–2.452E+00	–2.301E+00	–4.317E+00	–1.490E+00	–1.043669	–6.670370
${\text {F16}}$	$p-value$	*NA*	*NA*	*NA*	*NA*	*NA*	*NA*	*NA*	*NA*	*NA*
	$t-test$	0	0	0	0	0	0	0	0	0
${\text {F17}}$	$p-value$	0.000122	0.68782	0.000122	0.000122	0.1189	0.000122	0.039403	8.57E–05	0.000122
	$t-test$	5.097E+00	–9.910E–01	5.097E+00	5.097E+00	–5.554E–01	5.097E+00	–6.535E–01	5.432E+00	5.097E+00
${\text {F18}}$	$p-value$	*NA*	*NA*	*NA*	*NA*	*NA*	*NA*	*NA*	*NA*	*NA*
	$t-test$	0	0	0	0	0	0	0	0	$-2.664508$
${\text {F19}}$	$p-value$	6.629E–05	2.823E–03	6.629E–05	6.629E–05	4.464E–03	5.813E–01	5.802E–01	4.978350	6.629E–05
	$t-test$	4.997E+00	3.549E+00	4.997E+00	4.997E+00	2.593E+00	1.025E+00	–7.341E–01	4.978E+00	4.997E+00
${\text {F20}}$	$p-value$	0.000085	9.10E–05	9.10E–05	8.673E–05	8.673E–05	1.435E–04	9.10E–05	7.191E–05	8.131E–05
	$t-test$	1.125E+01	1.001E+01	1.059E+01	1.124E+01	1.016E+01	5.524E+00	9.920E+00	1.332E+01	1.208E+01
${\text {F21}}$	$p-value$	1.220E–04	9.10E–05	3.125E–02	1.651E–04	8.773E–05	4.515E–04	9.10E–05	8.139E–06	*NA*
	$t-test$	–6.304E+00	–6.656E+00	–2.797E+00	–3.559E+00	–1.454E+00	–1.474E+00	–3.565E+00	–7.881E+08	0
${\text {F22}}$	$p-value$	1.953E–03	9.10E–05	3.125E–02	3.908E–04	8.673E–05	9.10E–05	8.673E–05	8.140E–06	*NA*
	$t-test$	–4.214E+00	–3.772E+00	–2.787E+00	–4.358E+00	–7.756E+00	–3.059E+00	–1.463E+00	2.761E+10	0
${\text {F23}}$	$p-value$	3.125E–02	9.10E–05	NA	1.854E–04	8.283E–05	8.975E–05	9.10E–05	8.140E–06	*NA*
	$t-test$	–2.853E+00	–5.448E+00	–2.138E+00	–4.359E+00	–1.453E+00	–4.389E+00	–1.461E+00	5.677E+09	0
${\text {F24}}$	$p-value$	9.10E–05	1.651E–04	9.10E–05	*NA*	*NA*	*NA*	*NA*	*NA*	*NA*
	$t-test$	4.325E+02	–9.124E+00	2.746E+02	0	0	0	0	0	0
${\text {F25}}$	$p-value$	9.121E–05	9.113E–05	9.098E–05	9.090E–05	9.110E–05	9.130E–05	9.099E–05	9.1013E–05	9.121E–05
	$t-test$	–2.044E+00	–2.818E+00	–4.536E+00	–4.385E+00	–3.459E+00	–1.48E+00	–1.945E+00	–3.536E+00	–5.204E+00
${\text {F26}}$	$p-value$	4.569E–05	1.007E+00	2.505E–01	1.402E–02	1.402E–02	9.10E–05	9.10E–05	4.569E–05	1.070E–04
	$t-test$	1.388E+01	–2.840E+00	–2.174E+00	–1.448E+00	–1.448E+00	1.388E+01	1.386E+01	1.388E+01	9.901E+00
${\text {F27}}$	$p-value$	NA	0.00006103	NA	0.0009765	NA	NA	NA	NA	NA
	$t-test$	0	–2.516	0	–1.448	0	0	0	0	0

${\text {F28}}$	$p-value$	9.077E–05	9.077E–05	9.077E–05	9.077E–05	9.077E–05	NA	NA	9.207E–05	9.116E–05
	$t-test$	–2.26	0	–4.789	–7.09	0	0	0	–1.001894843	0
${\text {F29}}$	$p-value$	9.110E–05	9.120E–05	9.100E–05	9.130E–05	9.140E–05	9.110E–05	9.110E–05	*NA*	9.160E–05
	$t-test$	–6.454E+00	–3.562E+00	–1.536E+00	–3.318E+00	–6.512E+00	–3.927E+00	–7.644E+00	0	–1.701E+00
${\text {F30}}$	$p-value$	9.103E–05	1.005E–02	9.103E–05	6.370E–05	6.177E–02	2.455E–01	2.455E–01	6.370E–05	9.16E–05
	$t-test$	7.862E+02	–5.338E+00	1.338E+02	0	–4.358E+00	–2.179E+00	–2.179E+00	0	2.39E+307
${\text {F31}}$	$p-value$	9.120E–05	8.670E–02	9.110E–05	1.007E+00	1.007E+00	2.310E–01	1.007E+00	3.071E–01	9.180E–05
	$t-test$	5.556E+02	–3.547E+00	2.041E+02	–2.833E+00	–2.833E+00	–2.156E+00	–2.833E+00	–3.539E+00	2.08E+302
${\text {F32}}$	$p-value$	9.120E–05	9.110E–05	9.120E–05	9.100E–05	9.130E–05	9.110E–05	9.150E–05	9.140E–05	9.091E–05
	$t-test$	–3.23822	–1.47163	–8.62344	–6.91458	–1.75204	0	0	–1.27038	–6.64696
${\text {F33}}$	$p-value$	9.120E–05	9.098E–05	9.100E–05	9.110E–05	9.130E–05	9.120E–05	9.100E–05	9.190E–05	9.140E–05
	$t-test$	–5.611E+00	–4.319E+00	–4.521E+00	–1.069E+01	–4.00E+00	–2.283E+00	–1.577E+00	–5.179E+00	–6.842E+00
${\text {F34}}$	$p-value$	9.120E–05	9.120E–05	9.120E–05	9.120E–05	9.120E–05	9.120E–05	9.120E–05	*NA*	9.120E–05
	$t-test$	–2.185E+00	–5.339E+00	–4.389E+00	–3.175E+00	–7.751E+00	–2.467E+00	–2.850E+00	0	–3.335E+00
${\text {F35}}$	$p-value$	*NA*	*NA*	9.120E–05	9.130E–05	*NA*	*NA*	*NA*	*NA*	*NA*
	$t-test$	0	0	–5.725E+00	–5.671E+00	0	0	0	0	0
${\text {F36}}$	$p-value$	*NA*	9.120E–05	9.120E–05	9.120E–05	9.120E–05	9.120E–05	9.120E–05	*NA*	*NA*
	$t-test$	0	–1.453E+00	–4.238E+00	–5.266E+00	–6.003E+00	–5.496E+00	–4.192E+00	0	0
${\text {F37}}$	$p-value$	4.882E–04	7.813E–03	1.953E–03	*NA*	*NA*	*NA*	*NA*	*NA*	9.130E–05
	$t-test$	5.320E+00	$-3.559E+00$	4.350E+00	0	0	0	0	0	6.810E+00
${\text {F38}}$	$p-value$	9.10E–05	4.600E–04	4.600E–04	9.10E–05	9.10E–05	2.254E–03	9.10E–05	8.67E–05	9.19E–05
	$t-test$	4.698E+01	5.897E+00	5.241E+00	1.199E+01	4.094E+01	4.252E+00	9.474E+00	5.074E+01	2.537E+01
${\text {F39}}$	$p-value$	9.120E–05	9.110E–05	9.130E–05	9.101E–05	9.110E–05	2.814E–02	3.724E–02	8.975E–05	1.250E–04
	$t-test$	2.081E+01	5.084E+00	4.484E+00	1.099E+01	7.793E+00	1.814E+00	2.086E+00	4.142E+01	7.393E+00
${\text {F40}}$	$p-value$	*NA*	*NA*	*NA*	9.130E–05	*NA*	*NA*	*NA*	*NA*	9.210E–05
	$t-test$	0	0	–3.942E+00	–4.098E+00	0	0	0	0	–5.435E+00
${\text {F41}}$	$p-value$	*NA*	*NA*	9.120E–05	9.130E–05	*NA*	*NA*	*NA*	*NA*	9.206E–05
	$t-test$	0	0	–3.761E+00	–4.321E+00	0	0	0	0	–4.039E+00
${\text {F42}}$	$p-value$	*NA*	*NA*	9.130E–05	9.110E–05	*NA*	*NA*	*NA*	*NA*	9.170E–05
	$t-test$	0	–2.179E+00	–4.487E+00	–5.256E+00	0	–5.318E+00	0	0	–7.089E+00
${\text {F43}}$	$p-value$	*NA*	9.10E–05	*NA*	*NA*	4.436E–04	*NA*	1.990E–04	8.139E–06	8.641E–05
	$t-test$	0	–5.743E+00	0	0	–2.997E+00	0	–2.719E+00	–2.748E+08	–6.281E+00
${\text {F44}}$	$p-value$	*NA*	*NA*	9.120E–05	*NA*	*NA*	*NA*	*NA*	*NA*	9.160E–05
	$t-test$	0	0	–3.521E+00	0	0	0	0	0	–1.679E+00
${\text {F45}}$	$p-value$	*NA*	9.120E–05	9.130E–05	9.110E–05	9.100E–05	9.120E–05	9.160E–05	*NA*	9.130E–05
	$t-test$	0	–2.524E+00	–2.330E+00	–4.212E+00	–4.812E+00	–1.912E+00	–2.950E+00	0	–2.096E+00
${\text {F46}}$	$p-value$	*NA*	*NA*	*NA*	*NA*	*NA*	*NA*	*NA*	*NA*	*NA*
	$t-test$	0	–2.179E+00	0	0	0	0	–2.179E+00	0	0
${\text {F47}}$	$p-value$	9.150E–05	9.120E–05	9.130E–05	9.110E–05	9.160E–05	1.435E–04	1.435E–04	9.180E–05	9.083E–05
	$t-test$	3.169E+00	–3.485E+00	–3.838E+00	–7.052E–01	–1.436E+00	–3.487E+00	–2.047E+00	3.169E+00	3.164E+00
${\text {F48}}$	$p-value$	9.130E–05	9.150E–05	3.256E–03	9.160E–05	9.140E–05	9.120E–05	6.966E–04	9.140E–05	9.210E–05
	$t-test$	5.362E+01	5.186E+00	–1.993E+00	3.729E+00	9.452E+00	–2.954E+00	–1.258E+00	5.362E+01	2.851E+01
${\text {F49}}$	$p-value$	*NA*	9.110E–05	9.130E–05	9.160E–05	9.120E–05	9.110E–05	9.150E–05	*NA*	9.140E–05
	$t-test$	–2.171E+00	–2.849E+00	–3.201E+00	–3.418E+00	–3.390E+00	–1.959E+00	–2.656E+00	0	–2.087E+00
${\text {F50}}$	$p-value$	*NA*	9.120E–05	9.130E–05	9.140E–05	9.160E–05	9.130E–05	9.110E–05	*NA*	9.120E–05
	$t-test$	0	–2.298E+00	–3.114E+00	–1.540E+00	–1.710E+00	–1.599E+00	–3.317E+00	0	–1.712E+00
${\text {F51}}$	$p-value$	*NA*	9.140E–05	9.160E–05	9.120E–05	9.110E–05	9.180E–05	9.120E–05	*NA*	9.140E–05
	$t-test$	–1.452E+00	–3.039E+00	–4.451E+00	–3.149E+00	–2.739E+00	–3.139E+00	–4.335E+00	0	–1.559E+00

**Table 14:** Wilcoxon rank–sum test and t-test results for LCA versus recent algorithms for 51 benchmark functions.

Functions		LCA vs	LCA vs	LCA vs	LCA vs	LCA vs	LCA vs	LCA vs	LCA vs
		IGWO	MWOA	TLBO	MTBO	BWO	HHO	MGO	SCSO
${\text {F1}}$	$p-value$	9.207E–05	9.207E–05	9.207E–05	9.207E–05	*NA*	9.207E–05	9.207E–05	9.207E–05
	$t-test$	–1.763E+00	–1.000E+00	0	–1.022E+00	0	0	–1.370E+00	0
${\text {F2}}$	$p-value$	9.207E–05	9.207E–05	9.207E–05	9.207E–05	9.207E–05	9.207E–05	9.207E–05	9.207E–05
	$t-test$	–5.016E+00	–1.238E+00	–2.380E+00	–1.603E+00	0	–1.368E+00	–2.021E+00	–1.170E+00
${\text {F3}}$	$p-value$	9.207E–05	9.207E–05	9.207E–05	9.207E–05	*NA*	9.207E–05	9.207E–05	9.207E–05
	$t-test$	–1.530E+00	–9.865E+00	–3.498E+00	–3.487E+00	0	–1.000E+00	–1.150E+00	0
${\text {F4}}$	$p-value$	9.207E–05	9.207E–05	9.207E–05	9.207E–05	9.207E–05	9.207E–05	9.207E–05	9.207E–05
	$t-test$	–3.149E+00	–6.969E+00	–3.963E+00	–1.489E+01	0	–1.000E+00	–1.531E+00	–1.017E+00
${\text {F5}}$	$p-value$	9.207E–05	9.207E–05	9.207E–05	9.207E–05	9.207E–05	9.207E–05	9.207E–05	9.207E–05
	$t-test$	–5.212E+02	–3.337E+02	–1.552E+02	–8.142E+00	–1.193E+00	–4.900E+00	–1.916E+00	–1.650E+02
${\text {F6}}$	$p-value$	9.207E–05	9.207E–05	9.207E–05	9.207E–05	9.207E–05	9.207E–05	9.207E–05	9.207E–05
	$t-test$	–9.998E+00	–3.888E+00	–1.866E+00	–1.462E+00	–2.308E+00	–2.829E+00	–1.208E+00	–2.114E+01
${\text {F7}}$	$p-value$	9.207E–05	1.251E–04	9.207E–05	9.207E–05	8.259E–03	9.207E–05	9.207E–05	9.207E–05
	$t-test$	–1.429E+01	–3.814E+00	–1.097E+01	–9.742E+00	–3.488E+00	–1.615E+00	–6.536E+00	–2.104E+00
${\text {F8}}$	$p-value$	9.207E–05	9.207E–05	9.207E–05	9.207E–05	8.139E–06	1.074E–04	8.139E–06	9.207E–05
	$t-test$	–1.163E+01	–1.279E+01	–3.337E+01	–2.168E+01	1.948E+07	–2.639E+00	5.682E+05	–4.581E+01
${\text {F9}}$	$p-value$	9.207E–05	NA	1.373E–04	9.207E–05	*NA*	*NA*	*NA*	*NA*
	$t-test$	–1.136E+01	0	–9.747E+00	–1.854E+01	0	0	0	0
${\text {F10}}$	$p-value$	3.813E–05	3.813E–05	5.276E–05	9.207E–05	8.139E–06	8.139E–06	8.139E–06	8.139E–06
	$t-test$	–2.433E+01	–4.765E+00	–1.424E+01	–2.239E+01	–1.949E+00	–1.949E+00	–2.179E+00	–1.949E+00
${\text {F11}}$	$p-value$	4.883E–04	1.000E+00	NA	9.207E–05	NA	NA	NA	NA
	$t-test$	–3.599E+00	–1.000E+00	0	–3.597E+00	0	0	0	0
${\text {F12}}$	$p-value$	9.207E–05	9.207E–05	9.194E–05	9.207E–05	9.207E–05	9.207E–05	NA	9.207E–05
	$t-test$	–1.332E+01	–3.689E+00	–1.453E+00	–4.331E+00	–3.078E+00	–4.427E+00	–3.189E+11	–1.239E+01
${\text {F13}}$	$p-value$	9.207E–05	9.207E–05	9.142E–05	9.207E–05	9.207E–05	9.207E–05	NA	9.207E–05
	$t-test$	–1.832E+00	–6.255E+00	–4.574E+00	–5.870E+00	–2.635E+00	–2.462E+00	–1.154E+11	–3.808E+01
${\text {F14}}$	$p-value$	8.139E–06	7.022E–05	8.139E–06	8.139E–06	8.139E–06	1.792E–05	8.139E–06	6.312E–05
	$t-test$	–7.534E+10	–2.386E+00	–7.534E+10	–7.534E+10	–1.478E+07	–1.000E+00	–7.534E+10	–3.410E+00
${\text {F15}}$	$p-value$	1.504E–03	1.455E–04	7.245E–02	1.555E–03	2.630E–04	3.508E–04	7.159E–02	2.510E–01
	$t-test$	–9.253E–01	–3.063E+00	–9.984E–01	–1.280E+00	–4.042E+00	–4.427E+00	–1.785E+00	–1.449E+00
${\text {F16}}$	$p-value$	8.139E–06	8.139E–06	8.139E–06	8.139E–06	9.216E–03	8.139E–06	8.139E–06	8.139E–06
	$t-test$	2.793E+11	8.951E+05	2.793E+11	2.793E+11	–2.608E+00	2.849E+06	2.793E+11	1.262E+06

${\text {F17}}$	$p-value$	8.575E–05	9.181E–05	8.575E–05	8.575E–05	9.207E–05	9.181E–05	8.575E–05	9.181E–05
	$t-test$	5.426E+00	5.318E+00	5.426E+00	5.426E+00	–4.235E+00	5.402E+00	5.426E+00	5.424E+00
${\text {F18}}$	$p-value$	NA	1.373E–04	NA	NA	9.207E–05	NA	NA	9.207E–05
	$t-test$	3.879E+02	–1.571E+00	3.879E+02	3.879E+02	–3.427E+00	–1.710E+00	1.369E+02	–4.464E+00
${\text {F19}}$	$p-value$	6.629E–05	1.587E–01	6.629E–05	6.629E–05	4.168E–01	4.166E–03	6.629E–05	1.191E–01
	$t-test$	4.978E+00	1.206E+00	–3.815E+03	4.978E+00	9.358E–01	3.901E+00	4.978E+00	1.543E+00
${\text {F20}}$	$p-value$	8.824E–05	9.207E–05	7.225E–05	8.612E–05	9.207E–05	1.074E–04	8.649E–05	9.207E–05
	$t-test$	1.251E+01	1.060E+01	1.301E+01	1.140E+01	1.239E+01	8.124E+00	1.100E+01	1.002E+01
${\text {F21}}$	$p-value$	1.792E–05	9.207E–05	1.252E–05	7.118E–05	9.129E–05	9.207E–05	8.139E–06	9.207E–05
	$t-test$	–1.000E+00	–4.238E+00	–1.000E+00	–5.286E+00	–6.203E+00	–3.939E+04	–7.875E+08	–7.917E+00
${\text {F22}}$	$p-value$	1.252E–05	9.207E–05	5.427E–02	5.957E–01	9.194E–05	9.207E–05	8.139E–06	4.043E–04
	$t-test$	3.922E+05	–4.777E+00	–1.445E+00	–3.183E+00	1.213E+02	–1.345E+01	4.977E+10	–7.719E+00
${\text {F23}}$	$p-value$	8.139E–06	9.207E–05	4.088E–04	5.659E–01	9.207E–05	9.207E–05	8.139E–06	1.961E–04
	$t-test$	1.871E+09	–5.936E+00	–1.000E+00	–2.512E+00	4.571E+01	–1.434E+01	5.678E+09	–1.038E+01
${\text {F24}}$	$p-value$	*NA*	*NA*	*NA*	*NA*	*NA*	*NA*	*NA*	*NA*
	$t-test$	0	0	0	0	0	0	0	0
${\text {F25}}$	$p-value$	9.207E–05	9.207E–05	9.207E–05	9.207E–05	NA	9.207E–05	9.207E–05	9.207E–05
	$t-test$	–2.451E+00	–1.001E+00	0	–1.284E+00	0	0	–1.041E+00	0
${\text {F26}}$	$p-value$	4.570E–05	1.410E–02	4.570E–05	4.570E–05	1.820E–01	9.078E–05	4.570E–05	9.207E–05
	$t-test$	1.388E+01	–1.448E+00	1.388E+01	1.388E+01	–2.192E+00	1.388E+01	1.388E+01	1.388E+01
${\text {F27}}$	$p-value$	NA	2.033E–04	NA	NA	NA	9.194E–05	NA	1.780E–04
	$t-test$	0	–1.961E+00	0	0	0	–3.215E+00	0	–3.886E+00
${\text {F28}}$	$p-value$	9.207E–05	NA	9.207E–05	9.207E–05	NA	9.207E–05	NA	NA
	$t-test$	0	0	0	–1.013E+00	0	0	0	0
${\text {F29}}$	$p-value$	9.207E–05	9.207E–05	9.207E–05	9.207E–05	9.207E–05	9.207E–05	9.207E–05	9.207E–05
	$t-test$	–5.334E+00	–3.372E+00	–2.291E+00	–1.877E+00	–3.875E+00	–4.152E+00	–3.365E+00	–5.027E+00
${\text {F30}}$	$p-value$	1.217E–03	3.173E–01	1.273E–03	1.273E–03	9.207E–05	2.451E–01	6.371E–05	8.416E–05
	$t-test$	–9.997E–01	–3.559E+00	–9.997E–01	–9.997E–01	–4.094E–02	–2.179E+00	0	–7.670E–07
${\text {F31}}$	$p-value$	1.788E–05	3.163E–01	1.788E–05	8.926E–03	2.273E–04	1.391E–01	1.788E–05	2.415E–05
	$t-test$	1.083E–05	–3.559E+00	1.083E–05	–1.453E+00	–4.768E+00	–3.943E+00	1.083E–05	9.499E–06
${\text {F32}}$	$p-value$	9.207E–05	9.207E–05	9.207E–05	9.207E–05	NA	9.207E–05	9.207E–05	9.207E–05
	$t-test$	–1.093E+00	0	0	–1.138E+00	0	0	0	0
${\text {F33}}$	$p-value$	9.207E–05	9.207E–05	9.207E–05	9.207E–05	NA	9.207E–05	9.207E–05	9.207E–05
	$t-test$	–4.446E+00	–2.581E+00	–2.200E+00	–2.337E+00	0	0	–1.000E+00	0

${\text {F34}}$	$p-value$	9.207E–05	9.207E–05	9.207E–05	9.194E–05	9.207E–05	9.207E–05	*NA*	9.207E–05
	$t-test$	–2.139E+00	–1.044E+00	–1.007E+00	–1.025E+00	–2.991E+00	–1.038E+00	0	–1.602E+00
${\text {F35}}$	$p-value$	*NA*	*NA*	*NA*	*NA*	*NA*	*NA*	*NA*	*NA*
	$t-test$	0	0	0	0	0	0	0	0
${\text {F36}}$	$p-value$	9.207E–05	9.207E–05	9.207E–05	9.207E–05	9.207E–05	9.207E–05	9.207E–05	9.207E–05
	$t-test$	0	–3.164E+00	0	0	–5.381E+00	–3.079E+00	0	–4.146E+00
${\text {F37}}$	$p-value$	8.139E–06	1.551E–03	8.139E–06	8.139E–06	9.207E–05	9.014E–05	8.139E–06	9.103E–05
	$t-test$	4.734E+10	–9.999E–01	4.734E+10	4.734E+10	–4.939E+00	1.429E+02	4.734E+10	7.124E+02
${\text {F38}}$	$p-value$	9.207E–05	1.691E–04	9.129E–05	9.168E–05	9.207E–05	9.207E–05	9.039E–05	7.042E–04
	$t-test$	5.075E+01	6.035E+00	4.058E+01	2.099E+01	3.950E+01	6.918E+00	4.149E+01	4.416E+00
${\text {F39}}$	$p-value$	9.207E–05	3.412E–02	9.207E–05	9.207E–05	1.732E–02	2.274E–03	9.207E–05	5.900E–03
	$t-test$	2.761E+01	2.051E+00	2.786E+01	2.069E+01	2.744E+00	2.451E+00	2.956E+01	3.218E+00
${\text {F40}}$	$p-value$	*NA*	*NA*	*NA*	*NA*	*NA*	*NA*	*NA*	*NA*
	$t-test$	0	0	0	0	0	0	0	0
${\text {F41}}$	$p-value$	*NA*	*NA*	*NA*	*NA*	*NA*	*NA*	*NA*	*NA*
	$t-test$	0	0	0	0	0	0	0	0
${\text {F42}}$	$p-value$	NA	7.813E–03	*NA*	*NA*	*NA*	*NA*	*NA*	*NA*
	$t-test$	0	–2.603E+00	0	0	0	0	0	0
${\text {F43}}$	$p-value$	9.207E–05	4.883E–04	9.207E–05	9.207E–05	9.207E–05	1.563E–02	NA	4.456E–04
	$t-test$	–2.015E+00	–1.475E+00	–6.554E+00	–3.704E+00	–3.292E+00	–1.602E+00	0	–2.259E+00
${\text {F44}}$	$p-value$	*NA*	*NA*	*NA*	*NA*	*NA*	*NA*	*NA*	*NA*
	$t-test$	0	0	0	0	0	0	0	0
${\text {F45}}$	$p-value$	NA	9.207E–05	NA	NA	9.207E–05	NA	NA	9.207E–05
	$t-test$	0	–1.973E+00	0	0	–2.486E+00	0	0	–2.926E+00
${\text {F46}}$	$p-value$	NA	1.000E+00	NA	NA	9.207E–05	NA	NA	7.813E–03
	$t-test$	0	–1.000E+00	0	0	–2.714E+00	–1.540E+00	0	–1.000E+00
${\text {F47}}$	$p-value$	9.078E–05	9.207E–05	9.207E–05	9.091E–05	9.207E–05	9.207E–05	9.078E–05	9.207E–05
	$t-test$	3.169E+00	–4.549E+00	–3.611E+01	1.940E+00	2.770E+00	3.114E+00	3.169E+00	–3.791E+00
${\text {F48}}$	$p-value$	9.181E–05	9.207E–05	9.168E–05	9.207E–05	9.207E–05	9.207E–05	9.091E–05	9.207E–05
	$t-test$	5.362E+01	–4.484E+00	2.372E+01	1.486E+01	–1.678E+01	1.174E+01	4.116E+01	–1.217E–01
${\text {F49}}$	$p-value$	NA	9.207E–05	NA	1.000E+00	9.207E–05	NA	NA	9.207E–05
	$t-test$	0	–2.277E+00	0	–1.000E+00	–1.669E+00	0	0	–2.793E+00
${\text {F50}}$	$p-value$	NA	9.207E–05	NA	NA	9.207E–05	NA	NA	9.207E–05
	$t-test$	0	–2.062E+00	0	0	–3.150E+00	0	0	–1.692E+00
${\text {F51}}$	$p-value$	NA	9.207E–05	NA	NA	9.207E–05	NA	NA	9.207E–05
	$t-test$	0	–2.064E+00	0	0	–1.940E+00	0	0	–1.352E+00

**Table 15:** Wilcoxon rank–sum test and t-test results of well–known & top-performing algorithms for the CEC 2019 benchmark functions.

Functions		LCA vs	LCA vs	LCA vs	LCA vs	LCA vs	LCA vs	LCA vs	LCA vs	LCA vs
		PSO	TSA	SSA	MVO	GWO	WOA	GJO	LSHADE	CMAES
${\text {CEC19-1}}$	$p-value$	9.155E–05	4.024E–04	9.155E–05	9.155E–05	1.244E–04	9.155E–05	2.569E–02	9.207E–05	9.207E–05
	$t-test$	–4.764E+00	–1.162E+00	–2.248E+00	–6.907E+00	–1.651E+00	–3.586E+00	–2.132E+00	–2.155E+00	–6.732E+00
${\text {CEC19-2}}$	$p-value$	8.575E–05	3.812E–04	7.815E–05	8.575E–05	8.575E–05	7.815E–05	7.961E–02	7.815E–05	9.207E–05
	$t-test$	–2.061E+01	–4.010E+00	1.192E+01	–8.177E+00	1.192E+01	1.185E+01	2.818E+00	1.197E+01	–8.057E+00
${\text {CEC19-3}}$	$p-value$	*NA*	*NA*	*NA*	*NA*	*NA*	*NA*	*NA*	8.139E–06	9.207E–05
	$t-test$	0	–2.232E+00	–2.179E+00	0	0	0	–2.179E+00	–2.871E+09	–1.903E+00
${\text {CEC19-4}}$	$p-value$	8.575E–05	8.575E–05	8.575E–05	8.575E–05	8.575E–05	8.575E–05	8.575E–05	9.194E–05	9.207E–05
	$t-test$	1.877E+01	1.182E+01	1.872E+01	1.875E+01	1.871E+01	1.841E+01	1.782E+01	1.877E+01	1.876E+01
${\text {CEC19-5}}$	$p-value$	8.575E–05	3.812E–04	8.575E–05	8.575E–05	8.575E–05	8.575E–05	8.575E–05	9.181E–05	8.874E–05
	$t-test$	2.474E+01	1.063E+01	2.302E+01	2.385E+01	2.105E+01	1.623E+01	2.002E+01	2.566E+01	2.579E+01
${\text {CEC19-6}}$	$p-value$	8.575E–05	8.575E–05	8.575E–05	8.575E–05	1.863E–02	8.575E–05	7.961E–02	2.630E–04	5.814E–02
	$t-test$	1.067E+01	–4.543E+00	1.675E+01	1.196E+01	–1.864E+00	8.281E+00	–1.363E+00	8.134E+00	–1.648E+00
${\text {CEC19-7}}$	$p-value$	8.575E–05	1.863E–02	8.575E–05	8.575E–05	8.575E–05	3.159E–01	8.586E–01	9.194E–05	1.071E–04
	$t-test$	1.251E+01	1.747E+00	7.513E+00	1.221E+01	1.011E+01	–1.100E+00	1.692E–01	2.268E+01	6.374E+00
${\text {CEC19-8}}$	$p-value$	3.812E–04	8.575E–05	8.575E–05	8.575E–05	8.575E–05	1.682E–01	8.575E–05	9.207E–05	3.412E–02
	$t-test$	6.168E+00	–5.096E+00	7.508E+00	7.847E+00	8.951E+00	1.522E+00	5.632E+00	1.038E+01	2.750E+00
${\text {CEC19-9}}$	$p-value$	8.575E–05	8.575E–05	8.575E–05	8.575E–05	8.575E–05	8.575E–05	8.575E–05	9.207E–05	9.207E–05
	$t-test$	2.818E+01	2.624E+01	2.818E+01	2.818E+01	2.816E+01	2.816E+01	2.815E+01	2.818E+01	2.818E+01
${\text {CEC19-10}}$	$p-value$	8.575E–05	2.489E–01	8.575E–05	8.575E–05	8.586E–01	8.575E–05	1.682E–01	1.052E–04	1.915E–02
	$t-test$	1.821E+01	6.101E–01	2.452E+01	2.045E+01	6.958E–01	1.349E+01	1.010E+00	1.487E+01	2.018E+00

**Table 16:** Wilcoxon rank–sum test and t-test results of recent algorithms for the CEC 2019 benchmark functions.

Functions		LCA vs	LCA vs	LCA vs	LCA vs	LCA vs	LCA vs	LCA vs	LCA vs
		IGWO	MWOA	TLBO	MTBO	BWO	HHO	MGO	SCSO
${\text {CEC19-1}}$	$p-value$	9.207E–05	9.207E–05	1.074E–04	9.207E–05	1.074E–04	9.207E–05	9.207E–05	9.207E–05
	$t-test$	–4.358E+00	–3.325E+00	–3.484E+00	–3.560E+00	6.533E+00	9.935E+00	1.141E+01	1.142E+01
${\text {CEC19-2}}$	$p-value$	7.815E–05	9.207E–05	7.815E–05	7.815E–05	5.565E–01	9.207E–05	7.815E–05	3.701E–03
	$t-test$	1.197E+01	1.181E+01	1.197E+01	1.197E+01	5.551E–01	1.088E+01	1.197E+01	3.796E+00
${\text {CEC19-3}}$	$p-value$	2.401E–05	8.356E–05	8.139E–06	8.139E–06	9.207E–05	9.207E–05	8.139E–06	4.592E–05
	$t-test$	–4.880E+03	–2.269E+02	–2.871E+09	–4.125E+08	–7.272E+00	–1.055E+01	–7.626E+04	–3.220E+00
${\text {CEC19-4}}$	$p-value$	9.207E–05	9.207E–05	9.207E–05	9.207E–05	1.074E–04	9.207E–05	9.207E–05	9.207E–05
	$t-test$	1.874E+01	1.845E+01	1.873E+01	1.874E+01	8.417E+00	1.861E+01	1.875E+01	1.741E+01
${\text {CEC19-5}}$	$p-value$	9.207E–05	9.207E–05	9.207E–05	9.207E–05	4.043E–04	9.207E–05	9.207E–05	9.207E–05
	$t-test$	2.198E+01	1.834E+01	2.493E+01	2.435E+01	5.628E+00	1.198E+01	2.443E+01	2.127E+01
${\text {CEC19-6}}$	$p-value$	1.105E–01	2.006E–03	3.002E–01	1.024E–01	1.587E–01	6.142E–04	6.142E–04	9.207E–05
	$t-test$	–1.305E+00	3.461E+00	–1.080E+00	–1.531E+00	–1.604E+00	4.920E+00	2.880E+00	9.367E+00
${\text {CEC19-7}}$	$p-value$	1.961E–04	7.042E–04	7.042E–04	1.564E–02	3.701E–03	1.074E–04	9.207E–05	1.368E–03
	$t-test$	6.212E+00	5.269E+00	5.066E+00	2.958E+00	–3.861E+00	7.966E+00	1.138E+01	5.245E+00
${\text {CEC19-8}}$	$p-value$	9.207E–05	1.282E–01	9.207E–05	2.630E–04	4.096E–02	2.571E–02	9.207E–05	3.039E–04
	$t-test$	1.503E+01	1.492E+00	1.070E+01	5.732E+00	–2.431E+00	2.963E+00	8.491E+00	5.335E+00
${\text {CEC19-9}}$	$p-value$	9.207E–05	9.207E–05	9.207E–05	9.207E–05	9.207E–05	9.207E–05	9.207E–05	9.207E–05
	$t-test$	2.818E+01	2.816E+01	2.818E+01	2.818E+01	8.250E+00	2.818E+01	2.818E+01	2.816E+01
${\text {CEC19-10}}$	$p-value$	1.915E–02	1.074E–04	5.261E–03	1.270E–02	6.882E–01	9.207E–05	1.691E–04	9.207E–05
	$t-test$	1.516E+00	8.839E+00	1.589E+00	2.449E+00	4.510E–01	1.014E+01	1.607E+00	1.259E+01

**Table 17:** Average run time of algorithms for the 23 benchmark functions.

${\text {Functions}}$	${\text {PSO}}$	${\text {TSA}}$	${\text {SSA}}$	${\text {MVO}}$	${\text {GWO}}$	${\text {WOA}}$	${\text {GJO}}$	${\text {LSHADE}}$	${\text {CMAES}}$	${\text {IGWO}}$	${\text {MWOA}}$	${\text {TLBO}}$	${\text {MTBO}}$	${\text {BWO}}$	${\text {HHO}}$	${\text {MGO}}$	${\text {SCSO}}$	${\text {LCA}}$
F1	0.9576	0.1904	1.1758	3.4021	2.5490	3.1023	4.2120	0.7416	14.9193	13.4804	3.0892	14.9186	14.6459	3.5496	0.1812	20.3308	24.0783	7.0453
F2	0.9637	0.1754	1.2951	3.8382	2.3273	3.1838	3.6444	0.8112	16.0486	12.8719	3.0017	18.8409	13.5022	3.3428	0.1827	21.1138	24.8237	6.8798
F3	4.0986	0.4806	4.5122	5.9907	4.9915	8.5326	6.8330	0.9498	18.6081	21.9677	7.7960	23.3111	19.4771	7.1754	0.9417	33.6400	27.8261	13.9928
F4	0.9280	0.1675	1.2089	3.5064	1.9737	2.8278	3.0305	0.7504	16.1463	14.1519	3.0726	14.3570	14.3763	3.3440	0.2422	17.9461	23.7196	6.9197
F5	1.2191	0.2027	1.8679	3.6273	2.6580	3.2631	4.0253	0.7842	15.4064	15.8434	3.6479	15.8077	14.9568	3.3726	0.3733	24.7069	24.8905	7.8832
F6	0.9494	0.2482	1.4127	3.4057	2.4129	2.5866	3.6100	0.7583	16.0930	13.5480	2.9222	14.4743	13.7824	3.3609	0.2502	18.6578	22.9800	6.6388
F7	2.3244	0.4846	3.0177	5.1039	3.3412	5.0753	5.2573	0.9290	16.1532	15.8331	5.2201	20.8236	14.9042	4.9759	0.5326	24.9511	24.8621	9.1726
F8	1.3379	0.3120	1.6000	3.1931	2.9149	3.4783	3.9817	0.8733	15.6633	14.4551	3.8753	17.9256	13.5210	3.7703	0.3781	22.0281	24.6120	7.3900
F9	1.1361	0.1960	1.5697	3.3751	2.4434	2.8592	3.1879	0.8801	16.0788	13.4405	2.9459	15.8248	13.6856	3.4589	0.3038	20.2277	24.1072	7.0511
F10	1.2151	0.1827	1.8193	3.3804	2.7190	3.2624	3.2063	0.7634	15.0838	13.0447	3.1396	13.7098	13.6893	3.4267	0.3125	21.2541	23.7918	6.2017
F11	1.5008	0.2035	2.1827	4.2303	2.9037	3.7668	4.0750	1.0330	15.6576	14.2476	3.4948	13.8233	15.7720	3.8023	0.3365	22.1899	23.0459	7.8959
F12	4.7839	0.5455	5.1757	7.1462	6.5099	10.2604	8.2729	1.0850	18.6410	20.9325	10.3695	29.3525	24.2418	8.5333	1.2518	36.1212	28.4109	18.4887
F13	4.8872	0.5154	5.0948	13.6101	6.3576	10.3803	8.5042	1.1318	17.8957	22.4832	10.3139	29.3791	24.2126	8.0861	1.2028	36.9934	31.4010	15.9456
F14	8.6220	0.7985	9.2351	10.3337	9.4738	19.0502	11.6154	1.4361	17.8779	28.9677	19.3700	39.0952	31.5669	12.0991	2.4027	52.3612	11.6124	24.7273
F15	0.5821	0.0682	0.7475	1.4385	1.0706	2.8505	2.4235	0.6818	11.2006	11.1687	2.7305	13.9538	13.0399	2.1882	0.2312	17.3324	3.6218	5.9637
F16	0.5232	0.0619	0.7291	1.3190	0.7903	2.7374	2.2368	0.6690	11.2089	10.1304	2.6929	13.5535	11.7972	1.8329	0.2077	16.7226	1.9182	5.3508
F17	0.4066	0.0615	0.6319	1.5058	0.6725	2.0865	1.6348	0.6361	10.3822	9.8581	2.5298	12.2707	11.7723	1.5677	0.1836	15.5005	1.7988	4.9092
F18	0.4236	0.0865	0.6437	1.1986	0.6706	2.0697	1.8471	0.6911	10.4928	10.5208	2.5110	13.4106	11.5885	1.4528	0.1791	14.8699	2.0259	5.4934
F19	0.6888	0.0767	1.1119	1.3258	0.9779	2.5666	2.4019	0.6446	11.2635	11.5435	2.9165	15.3222	13.0433	2.0313	0.2568	17.4653	3.2594	6.4857
F20	0.7740	0.0993	0.9512	1.5388	1.1115	4.2550	2.6869	0.6969	10.7753	11.2991	3.0741	14.9380	13.0585	2.3543	0.3022	16.9430	5.4830	6.2252
F21	0.8050	0.1026	1.0418	1.8951	1.2122	5.0498	2.5520	0.7386	11.9403	11.2515	3.2478	15.9028	13.3741	2.6103	0.2994	18.6094	4.2174	6.2316
F22	0.9464	0.1421	1.1691	1.9977	1.2588	4.8136	2.8493	0.7697	11.9496	12.4454	3.4699	18.3558	13.7556	2.4140	0.3212	19.4354	4.2482	7.1654
F23	1.1186	0.1532	1.5387	2.1915	1.5397	3.6954	3.1444	0.7900	13.2197	12.2025	3.8097	19.4761	14.7273	2.8115	0.3664	19.6873	4.9575	7.4409

**Table 18:** Wilcoxon rank-sum test and t-test validation for LCA versus well-known & top-performing algorithms of 51 test functions.

Functions	LCA vs		LCA vs		LCA vs		LCA vs		LCA vs		LCA vs		LCA vs		LCA vs		LCA vs	
	PSO		TSA		SSA		MVO		GWO		WOA		GJO		LSHADE		CMAES	
	P	w/t/l	P	w/t/l	P	w/t/l	P	w/t/l	P	w/t/l	P	w/t/l	*P*	w/t/l	*P*	w/t/l	P	w/t/l
F1	$P+$	*w*	$P+$	*w*	$P+$	*w*	$P+$	*w*	$P+$	*w*	$P+$	*w*	$P+$	*w*	$P+$	*w*	$P+$	*w*
F2	$P+$	*w*	$P+$	*w*	$P+$	*w*	$P+$	*w*	$P+$	*w*	$P+$	*w*	$P+$	*w*	$P+$	*w*	$P+$	*w*
F3	$P+$	*w*	$P+$	*w*	$P+$	*w*	$P+$	*w*	$P+$	*w*	$P+$	*w*	$P+$	*w*	$P+$	*w*	$P+$	*w*
F4	$P+$	*w*	$P+$	*w*	$P+$	*w*	$P+$	*w*	$P+$	*w*	$P+$	*w*	$P+$	*w*	$P+$	*w*	$P+$	*w*
F5	$P+$	*w*	$P+$	*w*	$P+$	*w*	$P+$	*w*	$P+$	*w*	$P+$	*w*	$P+$	*w*	$P+$	*w*	$P+$	*w*
F6	$P+$	*w*	$P+$	*w*	$P+$	*w*	$P+$	*w*	$P+$	*w*	$P+$	*w*	$P+$	*w*	$P+$	*w*	$P+$	*w*
F7	$P+$	*w*	$P+$	*w*	$P+$	*w*	$P+$	*w*	$P+$	*w*	$P+$	*w*	$P+$	*w*	$P+$	*w*	$P+$	*w*
F8	$P+$	*w*	$P+$	*w*	$P+$	*w*	$P+$	*w*	$P+$	*w*	$P+$	*w*	$P+$	*w*	$P+$	*w*	$P+$	*w*
F9	$P+$	*w*	$P+$	*w*	$P+$	*w*	$P+$	*w*	$P+$	*w*	$P+$	*t*	$P+$	*t*	$P+$	*t*	$P+$	*w*
F10	$P+$	*w*	$P+$	*w*	$P+$	*w*	$P+$	*w*	$P+$	*w*	$P+$	*t*	$P+$	*t*	$P+$	*w*	$P+$	*t*
F11	$P+$	*w*	$P+$	*w*	$P+$	*w*	$P+$	*w*	$P+$	*w*	$P+$	*w*	$P+$	*w*	$P+$	*w*	$P+$	*w*
F12	$P+$	*w*	$P+$	*w*	$P+$	*w*	$P+$	*w*	$P+$	*w*	$P+$	*w*	$P+$	*w*	$P+$	*w*	$P+$	*w*
F13	$P+$	*w*	$P+$	*w*	$P+$	*w*	$P+$	*w*	$P+$	*w*	$P+$	*w*	$P+$	*w*	$P+$	*w*	$P+$	*w*
F14	$P+$	*w*	$P+$	*w*	$P+$	*w*	$P+$	*t*	$P+$	*w*	$P+$	*w*	$P+$	*w*	$P+$	*w*	$P+$	*w*
F15	$P+$	*w*	$P+$	*w*	$P+$	*w*	$P+$	*w*	$P-$	*w*	$P+$	*w*	$P-$	*w*	$P+$	*w*	$P+$	*w*
F16	$P+$	*t*	$P+$	*t*	$P+$	*t*	$P+$	*t*	$P+$	*t*	$P+$	*t*	$P+$	*t*	$P+$	*t*	$P+$	*t*
F17	$P+$	*l*	$P-$	*t*	$P+$	*l*	$P+$	*l*	$P-$	*t*	$P+$	*l*	$P+$	*t*	$P+$	*l*	$P+$	*l*
F18	$P+$	*t*	$P+$	*t*	$P+$	*t*	$P+$	*t*	$P+$	*t*	$P+$	*t*	$P+$	*t*	$P+$	*t*	$P+$	*w*
F19	$P+$	*l*	$P+$	*l*	$P+$	*l*	$P+$	*l*	$P+$	*l*	$P-$	*t*	$P-$	*t*	$P-$	*l*	$P+$	*l*
F20	$P+$	*l*	$P+$	*l*	$P+$	*l*	$P+$	*l*	$P+$	*l*	$P+$	*l*	$P+$	*l*	$P+$	*l*	$P+$	*l*
F21	$P+$	*w*	$P+$	*w*	$P+$	*w*	$P+$	*w*	$P+$	*w*	$P+$	*w*	$P+$	*w*	$P+$	*t*	$P+$	*t*
F22	$P+$	*w*	$P+$	*w*	$P+$	*w*	$P+$	*w*	$P+$	*w*	$P+$	*w*	$P+$	*w*	$P+$	*l*	$P+$	*w*
F23	$P+$	*w*	$P+$	*w*	$P+$	*w*	$P+$	*w*	$P+$	*w*	$P+$	*w*	$P+$	*w*	$P+$	*l*	$P+$	*t*
F24	$P+$	*l*	$P+$	*w*	$P+$	*l*	$P+$	*t*	$P+$	*t*	$P+$	*t*	$P+$	*t*	$P+$	*t*	$P+$	*t*
F25	$P+$	*w*	$P+$	*w*	$P+$	*w*	$P+$	*w*	$P+$	*w*	$P+$	*w*	$P+$	*w*	$P+$	*w*	$P+$	*w*
F26	$P+$	*l*	$P-$	*w*	$P-$	*w*	$P+$	*w*	$P+$	*w*	$P+$	*w*	$P+$	*l*	$P+$	*l*	$P+$	*l*
F27	$P+$	*t*	$P+$	*w*	$P+$	*t*	$P+$	*w*	$P+$	*w*	$P+$	*w*	$P+$	*w*	$P+$	*w*	$P+$	*w*
F28	$P+$	*w*	$P+$	*t*	$P+$	*w*	$P+$	*w*	$P+$	*t*	$P+$	*t*	$P+$	*w*	*t*	*t*	$P+$	*w*
F29	$P+$	*w*	$P+$	*w*	$P+$	*w*	$P+$	*w*	$P+$	*w*	$P+$	*w*	$P+$	*w*	$P+$	*t*	$P+$	*w*
F30	$P+$	*l*	$P+$	*w*	$P+$	*l*	$P+$	*t*	$P-$	*w*	$P-$	*w*	$P+$	*w*	$P+$	*t*	$P+$	*l*
F31	$P+$	*l*	$P-$	*w*	$P+$	*l*	$P-$	*w*	$P-$	*w*	$P-$	*w*	$P-$	*w*	$P-$	*w*	$P-$	*l*
F32	$P+$	*w*	$P+$	*w*	$P+$	*w*	$P+$	*w*	$P+$	*w*	$P+$	*w*	$P+$	*t*	$P+$	*w*	$P+$	*w*
F33	$P+$	*w*	$P+$	*w*	$P+$	*w*	$P+$	*w*	$P+$	*w*	$P+$	*w*	$P+$	*w*	$P+$	*w*	$P+$	*w*
F34	$P+$	*w*	$P+$	*w*	$P+$	*w*	$P+$	*w*	$P+$	*w*	$P+$	*w*	$P+$	*w*	$P+$	*t*	$P+$	*w*
F35	$P+$	*t*	$P+$	*t*	$P+$	*w*	$P+$	*w*	$P+$	*t*	$P+$	*t*	$P+$	*t*	$P+$	*t*	$P+$	*t*
F36	$P+$	*t*	$P+$	*w*	$P+$	*w*	$P+$	*w*	$P+$	*w*	$P+$	*w*	$P+$	*w*	$P+$	*t*	$P+$	*t*
F37	$P+$	*l*	$P+$	*w*	$P+$	*l*	$P+$	*t*	$P+$	*t*	$P+$	*t*	$P+$	*t*	$P+$	*t*	$P+$	*t*
F38	$P+$	*l*	$P+$	*l*	$P+$	*l*	$P+$	*l*	$P+$	*l*	$P+$	*l*	$P+$	*l*	$P+$	*l*	$P+$	*l*
F39	$P+$	*l*	$P+$	*l*	$P+$	*l*	$P+$	*l*	$P+$	*l*	$P+$	*l*	$P+$	*l*	$P+$	*l*	$P+$	*l*
F40	$P+$	*t*	$P+$	*t*	$P+$	*w*	$P+$	*w*	$P+$	*t*	$P+$	*t*	$P+$	*t*	$P+$	*t*	$P+$	*w*
F41	$P+$	*t*	$P+$	*t*	$P+$	*w*	$P+$	*w*	$P+$	*t*	$P+$	*t*	$P+$	*t*	$P+$	*t*	$P+$	*w*
F42	$P+$	*t*	$P+$	*w*	$P+$	*w*	$P+$	*w*	$P+$	*t*	$P+$	*w*	$P+$	*t*	$P+$	*t*	$P+$	*w*
F43	$P+$	*t*	$P+$	*w*	$P+$	*t*	$P+$	*t*	$P+$	*w*	$P+$	*t*	$P+$	*w*	$P+$	*w*	$P+$	*w*
F44	$P+$	*t*	$P+$	*t*	$P+$	*w*	$P+$	*t*	$P+$	*t*	$P+$	*t*	$P+$	*t*	$P+$	*t*	$P+$	*w*
F45	$P+$	*t*	$P+$	*w*	$P+$	*w*	$P+$	*w*	$P+$	*w*	$P+$	*w*	$P+$	*w*	$P+$	*t*	$P+$	*w*
F46	$P+$	*t*	$P+$	*w*	$P+$	*t*	$P+$	*t*	$P+$	*t*	$P+$	*t*	$P+$	*w*	$P+$	*t*	$P+$	*t*
F47	$P+$	*l*	$P+$	*l*	$P+$	*l*	$P+$	*l*	$P+$	*l*	$P+$	*l*	$P+$	*l*	$P+$	*l*	$P+$	*l*
F48	$P+$	*l*	$P+$	*l*	$P+$	*l*	$P+$	*l*	$P+$	*l*	$P+$	*l*	$P+$	*l*	$P+$	*l*	$P+$	*l*
F49	$P+$	*w*	$P+$	*w*	$P+$	*w*	$P+$	*w*	$P+$	*w*	$P+$	*w*	$P+$	*w*	$P+$	*t*	$P+$	*w*
F50	$P+$	*w*	$P+$	*w*	$P+$	*w*	$P+$	*w*	$P+$	*w*	$P+$	*w*	$P+$	*w*	$P+$	*t*	$P+$	*w*
F51	$P+$	*w*	$P+$	*w*	$P+$	*w*	$P+$	*w*	$P+$	*w*	$P+$	*w*	$P+$	*w*	$P+$	*t*	$P+$	*w*

**Table 19:** Wilcoxon rank-sum test and t-test validation for LCA versus recent algorithms of 51 test functions.

Functions	LCA vs		LCA vs		LCA vs		LCA vs		LCA vs		LCA vs		LCA vs		LCA vs	
	IGWO		MWOA		TLBO		MTBO		BWO		HHO		MGO		**SCSO**	
	P	w/t/l	P	w/t/l	P	w/t/l	P	w/t/l	P	w/t/l	P	w/t/l	P	*w*/*t*/*l*	P	w/t/l
F1	$P+$	*w*	$P+$	*t*	$P+$	*t*	$P+$	*t*	$P+$	*t*	$P+$	*t*	$P+$	*w*	$P+$	*t*
F2	$P+$	*w*	$P+$	*w*	$P+$	*w*	$P+$	*w*	$P+$	*t*	$P+$	*w*	$P+$	*w*	$P+$	*w*
F3	$P+$	*w*	$P+$	*w*	$P+$	*w*	$P+$	*w*	$P+$	*t*	$P+$	*t*	$P+$	*w*	$P+$	*t*
F4	$P+$	*w*	$P+$	*w*	$P+$	*w*	$P+$	*w*	$P+$	*t*	$P+$	*t*	$P+$	*w*	$P+$	*w*
F5	$P+$	*w*	$P+$	*w*	$P+$	*w*	$P+$	*w*	$P+$	*w*	$P+$	*w*	$P+$	*w*	$P+$	*w*
F6	$P+$	*w*	$P+$	*w*	$P+$	*w*	$P+$	*w*	$P+$	*w*	$P+$	*w*	$P+$	*w*	$P+$	*w*
F7	$P+$	*w*	$P+$	*w*	$P+$	*w*	$P+$	*w*	$P+$	*w*	$P+$	*w*	$P+$	*w*	$P+$	*w*
F8	$P+$	*w*	$P+$	*w*	$P+$	*w*	$P+$	*w*	$P+$	*l*	$P+$	*w*	$P+$	*l*	$P+$	*t*
F9	$P+$	*w*	$P+$	*t*	$P+$	*w*	$P+$	*w*	$P+$	*t*	$P+$	*t*	$P+$	*t*	$P+$	*l*
F10	$P+$	*w*	$P+$	*w*	$P+$	*w*	$P+$	*w*	$P+$	*w*	$P+$	*w*	$P+$	*w*	$P+$	*w*
F11	$P+$	*w*	$P-$	*t*	$P+$	*t*	$P+$	*w*	$P+$	*t*	$P+$	*t*	$P+$	*t*	$P+$	*t*
F12	$P+$	*w*	$P+$	*w*	$P+$	*w*	$P+$	*w*	$P+$	*w*	$P+$	*w*	$P+$	*w*	$P+$	*w*
F13	$P+$	*w*	$P+$	*w*	$P+$	*w*	$P+$	*w*	$P+$	*w*	$P+$	*w*	$P+$	*w*	$P+$	*w*
F14	$P+$	*w*	$P+$	*w*	$P+$	*w*	$P+$	*w*	$P+$	*w*	$P+$	*t*	$P+$	*w*	$P+$	*w*
F15	$P+$	*t*	$P+$	*w*	$P+$	*t*	$P+$	*w*	$P+$	*w*	$P+$	*w*	$P-$	*w*	$P-$	*l*
F16	$P+$	*l*	$P+$	*l*	$P+$	*l*	$P+$	*l*	$P+$	*l*	$P+$	*l*	$P+$	*l*	$P+$	*l*
F17	$P+$	*l*	$P+$	*l*	$P+$	*l*	$P+$	*l*	$P+$	*w*	$P+$	*l*	$P+$	*l*	$P+$	*w*
F18	$P+$	*l*	$P+$	*w*	$P+$	*l*	$P+$	*l*	$P+$	*w*	$P+$	*w*	$P+$	*l*	$P+$	*l*
F19	$P+$	*l*	$P+$	*l*	$P+$	*w*	$P+$	*l*	$P+$	*t*	$P+$	*l*	$P+$	*l*	$P+$	*l*
F20	$P+$	*l*	$P+$	*l*	$P+$	*l*	$P+$	*l*	$P+$	*l*	$P+$	*l*	$P+$	*l*	$P+$	*w*
F21	$P+$	*t*	$P+$	*w*	$P+$	*t*	$P+$	*w*	$P+$	*w*	$P+$	*w*	$P+$	*w*	$P+$	*w*
F22	$P+$	*l*	$P+$	*w*	$P+$	*w*	$P+$	*w*	$P+$	*l*	$P+$	*w*	$P+$	*l*	$P+$	*w*
F23	$P+$	*l*	$P+$	*w*	$P+$	*t*	$P-$	*w*	$P+$	*l*	$P+$	*w*	$P+$	*l*	$P+$	*t*
F24	$P+$	*t*	$P+$	*t*	$P+$	*t*	$P+$	*t*	$P+$	*t*	$P+$	*t*	$P+$	*t*	$P+$	*t*
F25	$P+$	*w*	$P+$	*t*	$P+$	*t*	$P+$	*t*	$P+$	*t*	$P+$	*t*	$P+$	*t*	$P+$	*t*
F26	$P+$	*l*	$P+$	*w*	$P+$	*l*	$P+$	*l*	$P-$	*w*	$P+$	*l*	$P+$	*l*	$P+$	*l*
F27	$P+$	*t*	$P+$	*w*	$P+$	*t*	$P+$	*t*	$P+$	*t*	$P+$	*w*	$P+$	*t*	$P+$	*w*
F28	$P+$	*t*	$P+$	*t*	$P+$	*t*	$P+$	*t*	$P+$	*t*	$P+$	*t*	$P+$	*t*	$P+$	*t*
F29	$P+$	*w*	$P+$	*w*	$P+$	*w*	$P+$	*w*	$P+$	*w*	$P+$	*w*	$P+$	*w*	$P+$	*w*
F30	$P+$	*t*	$P-$	*w*	$P+$	*t*	$P-$	*t*	$P+$	*t*	$P-$	*w*	$P+$	*t*	$P+$	*t*
F31	$P+$	*t*	$P-$	*w*	$P+$	*t*	$P-$	*w*	$P+$	*w*	$P-$	*w*	$P+$	*t*	$P+$	*t*
F32	$P+$	*w*	$P+$	*t*	$P+$	*t*	$P+$	*w*	$P+$	*t*	$P+$	*t*	$P+$	*t*	$P+$	*t*
F33	$P+$	*w*	$P+$	*w*	$P+$	*w*	$P+$	*w*	$P+$	*t*	$P+$	*t*	$P+$	*t*	$P+$	*t*
F34	$P+$	*w*	$P-$	*t*	$P+$	*t*	$P-$	*t*	$P+$	*w*	$P+$	*t*	$P+$	*t*	$P+$	*w*
F35	$P+$	*t*	$P+$	*t*	$P+$	*t*	$P+$	*t*	$P+$	*t*	$P+$	*t*	$P+$	*t*	$P+$	*t*
F36	$P+$	*t*	$P+$	*w*	$P+$	*t*	$P+$	*t*	$P+$	*w*	$P+$	*w*	$P+$	*t*	$P+$	*w*
F37	$P+$	*l*	$P+$	*t*	$P+$	*l*	$P+$	*l*	$P+$	*l*	$P+$	*l*	$P+$	*l*	$P+$	*l*
F38	$P+$	*l*	$P+$	*l*	$P+$	*l*	$P+$	*l*	$P+$	*l*	$P+$	*l*	$P+$	*l*	$P+$	*l*
F39	$P+$	*l*	$P+$	*l*	$P+$	*l*	$P+$	*l*	$P+$	*l*	$P+$	*l*	$P+$	*l*	$P+$	*l*
F40	$P+$	*t*	$P+$	*t*	$P+$	*t*	$P+$	*t*	$P+$	*t*	$P+$	*t*	$P+$	*t*	$P+$	*t*
F41	$P+$	*t*	$P+$	*t*	$P+$	*t*	$P+$	*t*	$P+$	*t*	$P+$	*t*	$P+$	*t*	$P+$	*t*
F42	$P+$	*t*	$P+$	*w*	$P+$	*w*	$P+$	*t*	$P+$	*t*	$P+$	*t*	$P+$	*t*	$P+$	*t*
F43	$P+$	*w*	$P+$	*w*	$P+$	*t*	$P+$	*w*	$P+$	*w*	$P+$	*w*	$P+$	*t*	$P+$	*w*
F44	$P+$	*t*	$P+$	*t*	$P+$	*t*	$P-$	*t*	$P+$	*t*	$P+$	*t*	$P+$	*t*	$P+$	*t*
F45	$P+$	*t*	$P+$	*w*	$P+$	*t*	$P+$	*t*	$P+$	*w*	$P+$	*t*	$P+$	*t*	$P+$	*w*
F46	$P+$	*t*	$P-$	*t*	$P+$	*t*	$P+$	*t*	$P+$	*w*	$P+$	*w*	$P+$	*w*	$P+$	*t*
F47	$P+$	*l*	$P+$	*w*	$P+$	*w*	$P+$	*l*	$P+$	*l*	$P+$	*l*	$P+$	*l*	$P+$	*w*
F48	$P+$	*l*	$P+$	*w*	$P+$	*l*	$P+$	*l*	$P+$	*l*	$P+$	*l*	$P+$	*l*	$P+$	*w*
F49	$P+$	*t*	$P+$	*w*	$P+$	*t*	$P+$	*t*	$P+$	*t*	$P+$	*t*	$P+$	*t*	$P+$	*w*
F50	$P+$	*t*	$P+$	*w*	$P+$	*t*	$P+$	*w*	$P+$	*t*	$P+$	*t*	$P+$	*t*	$P+$	*w*
F51	$P+$	*t*	$P+$	*w*	$P+$	*t*	$P+$	*t*	$P+$	*w*	$P+$	*t*	$P+$	*t*	$P+$	*w*

**Table 20:** Results on Wilcoxon rank-sum test and t-test validation for LCA versus well-known & top-performing algorithms of CEC 2019 benchmark functions.

Functions	LCA vs		LCA vs		LCA vs		LCA vs		LCA vs		LCA vs		LCA vs		LCA vs		LCA vs	
	PSO		TSA		SSA		MVO		GWO		WOA		GJO		LSHADE		CMAES	
	P	w/t/l	P	w/t/l	P	w/t/l	P	w/t/l	P	w/t/l	P	w/t/l	P	w/t/l	P	w/t/l	P	w/t/l
CEC19-1	$P+$	*w*	$P+$	*w*	$P+$	*w*	$P+$	*w*	$P+$	*w*	$P+$	*w*	$P+$	*w*	$P+$	*w*	$P+$	*w*
CEC19-2	$P+$	*w*	$P+$	*w*	$P+$	*l*	$P+$	*w*	$P+$	*l*	$P+$	*l*	$P+$	*l*	$P+$	*l*	$P+$	*w*
CEC19-3	$P+$	*t*	$P+$	*w*	$P+$	*w*	$P+$	*t*	$P+$	*t*	$P+$	*t*	$P+$	*w*	$P+$	*w*	$P+$	*w*
CEC19-4	$P+$	*l*	$P+$	*l*	$P+$	*l*	$P+$	*l*	$P+$	*l*	$P+$	*l*	$P+$	*l*	$P+$	*l*	$P+$	*l*
CEC19-5	$P+$	*l*	$P+$	*l*	$P+$	*l*	$P+$	*l*	$P+$	*l*	$P+$	*l*	$P+$	*l*	$P+$	*l*	$P+$	*l*
CEC19-6	$P+$	*l*	$P+$	*w*	$P+$	*l*	$P+$	*l*	$P+$	*w*	$P+$	*l*	$P-$	*w*	$P+$	*l*	$P+$	*w*
CEC19-7	$P+$	*l*	$P+$	*l*	$P+$	*l*	$P+$	*l*	$P+$	*l*	$P-$	*w*	$P-$	*l*	$P+$	*l*	$P+$	*l*
CEC19-8	$P+$	*l*	$P+$	*w*	$P+$	*l*	$P+$	*l*	$P+$	*l*	$P-$	*l*	$P+$	*l*	$P+$	*l*	$P+$	*l*
CEC19-9	$P+$	*l*	$P+$	*l*	$P+$	*l*	$P+$	*l*	$P+$	*l*	$P+$	*l*	$P+$	*l*	$P+$	*l*	$P+$	*l*
CEC19-10	$P+$	*l*	$P-$	*l*	$P+$	*l*	$P+$	*l*	$P-$	*l*	$P+$	*l*	$P-$	*l*	$P+$	*l*	$P+$	*l*

**Table 21:** Results on Wilcoxon rank-sum test and t-test validation for LCA versus recent algorithms of CEC 2019 benchmark functions.

Functions	LCA vs		LCA vs		LCA vs		LCA vs		LCA vs		LCA vs		LCA vs		LCA vs	
	IGWO		MWOA		TLBO		MTBO		BWO		HHO		MGO		SCSO	
	P	w/t/l	P	w/t/l	P	w/t/l	P	w/t/l	*P*	w/t/l	P	w/t/l	P	*w*/*t*/*l*	P	w/t/l
CEC19-1	$P+$	*w*	$P+$	*w*	$P+$	*w*	$P+$	*w*	$P+$	*l*	$P+$	*l*	$P+$	*l*	$P+$	*l*
CEC19-2	$P+$	*l*	$P+$	*l*	$P+$	*l*	$P+$	*l*	$P+$	*t*	$P+$	*l*	$P+$	*l*	$P+$	*l*
CEC19-3	$P+$	*w*	$P+$	*w*	$P+$	*w*	$P+$	*w*	$P+$	*w*	$P+$	*w*	$P+$	*w*	$P+$	*w*
CEC19-4	$P+$	*l*	$P+$	*l*	$P+$	*l*	$P+$	*l*	$P+$	*l*	$P+$	*l*	$P+$	*l*	$P+$	*l*
CEC19-5	$P+$	*l*	$P+$	*l*	$P+$	*l*	$P+$	*l*	$P+$	*l*	$P+$	*l*	$P+$	*l*	$P+$	*l*
CEC19-6	$P-$	*w*	$P+$	*l*	$P-$	*w*	$P-$	*w*	$P-$	*w*	$P+$	*l*	$P+$	*l*	$P+$	*l*
CEC19-7	$P+$	*l*	$P+$	*l*	$P+$	*l*	$P+$	*l*	$P+$	*w*	$P+$	*l*	$P+$	*l*	$P+$	*l*
CEC19-8	$P+$	*l*	$P+$	*l*	$P+$	*l*	$P+$	*l*	$P+$	*w*	$P+$	*l*	$P+$	*l*	$P+$	*l*
CEC19-9	$P+$	*l*	$P-$	*l*	$P+$	*l*	$P+$	*l*	$P+$	*l*	$P+$	*l*	$P+$	*l*	$P+$	*l*
CEC19-10	$P+$	*l*	$P+$	*l*	$P+$	*l*	$P+$	*l*	$P+$	*t*	$P+$	*l*	$P+$	*l*	$P+$	*l*

In this experiment, the Friedman test^96,97^ is used to rank the performance of the algorithms under evaluation. This test ranks the value of each algorithm from lowest to greatest and evaluates if there is a significant difference between LCA and the comparative optimization algorithms. Table 22 shows the over-rank report of LCA with well-known and top-performing algorithms for 51 benchmark functions. Table 23 shows the over-rank report of LCA with recent algorithms for 51 benchmark functions. Table 24 shows the over-rank report of LCA with well-known and top-performing algorithms for CEC 2019 benchmark functions. Table 25 shows the over-rank report of LCA with recent algorithms for CEC 2019 benchmark functions. According to Table 22, LCA has the greatest overall capacity to solve these challenging problems, with a mean rank of 2.1372. According to Table 23, LCA has the greatest overall capacity to solve these challenging problems, with a mean rank of 1.9019. The results of the Friedman test again prove the superiority of LCA over the other considered optimizers.

**Table 22:** Rankings are based on t-tests of well-known & top-performing algorithms for the 51 benchmark functions.

${\text {Functions}}$	${\text {PSO}}$	${\text {TSA}}$	${\text {SSA}}$	${\text {MVO}}$	${\text {GWO}}$	${\text {WOA}}$	${\text {GJO}}$	${\text {LSHADE}}$	${\text {CMAES}}$	${\text {LCA}}$
F1	6	3	9	8	4	1	2	5	7	1
F2	8	3	6	10	7	2	5	4	9	1
F3	9	4	5	10	3	8	2	6	7	1
F4	10	3	6	7	5	4	2	9	8	1
F5	4	6	2	3	10	8	7	5	9	1
F6	6	10	7	9	8	4	3	2	5	1
F7	10	8	7	9	3	2	4	6	5	1
F8	5	7	1	9	9	3	6	4	2	2
F9	7	5	6	4	3	1	1	2	8	1
F10	2	3	5	6	9	4	10	8	7	1
F11	6	4	5	7	2	1	1	3	1	1
F12	5	9	8	6	7	4	10	2	3	1
F13	3	10	4	8	9	7	6	2	5	1
F14	5	6	2	1	3	4	9	8	7	1
F15	9	5	10	6	4	7	3	2	8	1
F16	1	1	1	1	1	1	1	1	1	1
F17	2	4	2	2	6	2	5	1	2	3
F18	1	1	1	1	1	1	1	1	2	1
F19	2	3	2	2	4	5	7	1	2	6
F20	3	7	5	4	6	9	8	1	2	10
F21	7	8	4	5	2	3	6	9	1	1
F22	6	6	4	7	8	5	3	1	2	2
F23	6	9	5	7	3	8	4	1	2	2
F24	1	4	2	3	3	3	3	3	3	3
F25	4	5	9	8	6	2	3	7	10	1
F26	1	7	6	5	5	1	2	1	3	4
F27	1	3	1	2	1	1	1	1	1	1
F28	3	1	4	5	1	1	1	2	1	1
F29	7	5	2	4	8	6	9	1	3	1
F30	2	7	3	4	6	5	5	4	1	4
F31	3	8	2	6	6	5	6	7	1	4
F32	5	3	8	7	4	1	1	2	6	1
F33	8	5	6	10	4	3	2	7	9	1
F34	2	8	7	5	9	3	4	1	6	1
F35	1	1	3	2	1	1	1	1	1	1
F36	1	2	4	5	7	6	3	1	1	1
F37	2	5	3	4	4	4	4	4	1	4
F38	2	6	7	3	8	9	5	1	4	10
F39	2	6	7	3	4	9	2	1	5	10
F40	1	1	2	3	1	1	1	1	4	1
F41	1	1	2	4	1	1	1	1	3	1
F42	1	2	3	4	1	5	1	1	6	1
F43	1	5	1	1	4	1	2	3	6	1
F44	1	1	3	1	1	1	1	1	2	1
F45	1	5	4	7	8	2	6	1	3	1
F46	1	2	1	1	1	1	2	1	1	1
F47	1	6	8	9	4	7	5	1	2	3
F48	1	4	8	5	3	9	7	1	2	6
F49	4	6	7	9	8	2	5	1	3	1
F50	1	6	7	2	4	3	8	1	5	1
F51	2	4	9	6	8	5	7	1	3	1
Sum of ranks	184	244	236	260	238	192	204	142	201	109
Mean of ranks	3.6078	4.7843	4.6274	5.0980	4.6666	3.7647	4.0000	2.7843	3.9411	2.1372
Overall ranks	3	9	7	10	8	4	6	2	5	1

**Table 23:** Rankings are based on t-tests of recent algorithms for the 51 benchmark functions.

${\text {Functions}}$	${\text {IGWO}}$	${\text {MWOA}}$	${\text {TLBO}}$	${\text {MTBO}}$	${\text {BWO}}$	${\text {HHO}}$	${\text {MGO}}$	${\text {SCSO}}$	${\text {LCA}}$
F1	5	2	1	3	1	1	4	1	1
F2	8	3	7	5	1	4	6	2	1
F3	4	7	6	5	1	2	3	1	1
F4	6	8	7	4	1	2	5	3	1
F5	8	6	3	9	2	7	5	4	1
F6	8	7	4	3	5	6	2	9	1
F7	9	5	8	7	4	2	6	3	1
F8	5	6	8	7	1	4	2	9	3
F9	3	1	2	4	1	1	1	1	1
F10	4	5	6	7	2	2	3	2	1
F11	4	2	1	3	1	1	1	1	1
F12	8	4	2	5	3	6	9	7	1
F13	2	7	5	6	4	3	9	8	1
F14	6	3	6	6	5	2	6	4	1
F15	2	7	3	4	8	9	6	5	1
F16	6	3	6	6	2	5	6	4	1
F17	2	3	2	2	5	4	2	2	1
F18	2	4	2	2	6	5	3	7	1
F19	1	4	7	1	6	2	1	3	5
F20	7	3	8	5	6	1	4	2	9
F21	2	4	2	5	6	3	7	8	1
F22	2	8	6	7	3	5	1	9	4
F23	2	7	5	6	3	9	1	8	4
F24	1	1	1	1	1	1	1	1	1
F25	5	2	1	4	1	1	3	1	1
F26	1	3	1	1	4	1	1	1	2
F27	1	2	1	1	1	3	1	4	1
F28	1	1	1	2	1	1	1	1	1
F29	9	5	3	2	6	7	4	8	1
F30	4	6	4	4	3	5	1	2	1
F31	3	5	3	4	7	6	3	2	1
F32	2	1	1	3	1	1	1	1	1
F33	6	5	3	4	1	1	2	1	1
F34	7	5	2	3	8	4	1	6	1
F35	1	1	1	1	1	1	1	1	1
F36	1	3	1	1	5	2	1	4	1
F37	2	5	2	2	6	4	2	3	1
F38	3	2	6	8	7	1	5	4	9
F39	4	8	3	7	5	6	2	1	9
F40	1	1	1	1	1	1	1	1	1
F41	1	1	1	1	1	1	1	1	1
F42	1	2	1	1	1	1	1	1	1
F43	4	2	8	7	6	3	1	5	1
F44	1	1	1	1	1	1	1	1	1
F45	1	2	1	1	3	1	1	4	1
F46	1	2	1	1	4	3	1	2	1
F47	1	8	6	4	3	2	1	7	5
F48	1	9	3	4	8	5	2	7	6
F49	1	4	1	2	3	1	1	5	1
F50	1	3	1	1	4	1	1	2	1
F51	1	4	1	1	3	1	1	2	1
Sum of ranks	172	203	167	185	173	152	136	182	97
Mean of ranks	3.3725	3.9803	3.2745	3.6274	3.3921	2.9803	2.6666	3.5686	1.9019
Overall ranks	5	9	4	8	6	3	2	7	1

**Table 24:** Rankings are based on t-tests of well-known & top-performing algorithms for the CEC 2019 benchmark functions.

${\text {Functions}}$	${\text {PSO}}$	${\text {TSA}}$	${\text {SSA}}$	${\text {MVO}}$	${\text {GWO}}$	${\text {WOA}}$	${\text {GJO}}$	${\text {LSHADE}}$	${\text {CMAES}}$	${\text {LCA}}$
CEC19-1	8	2	6	10	3	7	4	5	9	1
CEC19-2	7	6	3	9	3	4	1	2	8	5
CEC19-3	1	4	3	1	1	1	3	5	2	1
CEC19-4	1	8	4	3	5	6	7	1	2	9
CEC19-5	3	9	5	4	6	8	7	2	1	10
CEC19-6	4	9	4	3	8	1	6	2	7	5
CEC19-7	2	7	5	3	4	10	9	1	6	8
CEC19-8	5	10	4	3	2	8	6	1	7	9
CEC19-9	1	4	1	1	2	2	3	1	1	5
CEC19-10	4	10	1	2	9	6	7	5	3	8
Sum of ranks	36	69	36	39	43	53	53	25	46	61
Mean of ranks	3.6	6.9	3.6	3.9	4.3	5.3	5.3	2.5	4.6	6.1
Overall ranks	2	8	2	3	4	6	6	1	5	7

**Table 25:** Rankings are based on t-tests of recent algorithms for the CEC 2019 benchmark functions.

${\text {Functions}}$	${\text {IGWO}}$	${\text {MWOA}}$	${\text {TLBO}}$	${\text {MTBO}}$	${\text {BWO}}$	${\text {HHO}}$	${\text {MGO}}$	${\text {SCSO}}$	${\text {LCA}}$
CEC19-1	6	9	8	7	4	3	2	1	5
CEC19-2	1	2	1	1	6	3	1	4	5
CEC19-3	6	5	9	8	3	4	7	2	1
CEC19-4	2	1	3	2	6	4	1	5	7
CEC19-5	4	6	1	3	8	7	2	5	9
CEC19-6	7	3	6	8	9	2	4	1	5
CEC19-7	3	4	5	6	8	2	1	5	7
CEC19-8	1	7	2	4	6	9	3	5	8
CEC19-9	1	2	1	1	3	1	1	2	4
CEC19-10	7	3	6	4	9	2	5	1	8
Sum of ranks	38	42	42	44	62	37	27	31	59
Mean of ranks	3.8	4.2	4.2	4.4	6.2	3.7	2.7	3.1	5.9
Overall ranks	4	5	5	6	8	3	1	2	7

### Informed consent

I confirm that all these pictures belong to ourselves (the authors), and the people pictured have provided their consent for their likeness to be used in an online, open-access scientific journal. In the case of those under 16 years of age, consent has been provided by the parent/legal guardian.

## LCA for engineering optimization problems

The capability of LCA to provide the best solution for real optimization applications is discussed in this section. For this purpose, LCA and competing algorithms have been employed in seven real-life problems, such as tension/compression spring design, pressure vessel design problem, welded beam design problem, speed reducer design problem, gear train design problem, three-bar truss design, and cantilever beam problem. The performance of the LCA algorithm is compared with well-known and most recent algorithms such as PSO^14^, TSA^20^, SSA^80^, MVO^40^, GWO^15^, WOA^19^, GJO^81^, IGWO^93^, MWOA^94^, TLBO^6^, MTBO^86^, BWO^82^, HHO^83^, MGO^84^, and SCSO^85^ in order to evaluate the efficiency of the LCA results.

### Tension/compression spring design problem

**Figure 8:**
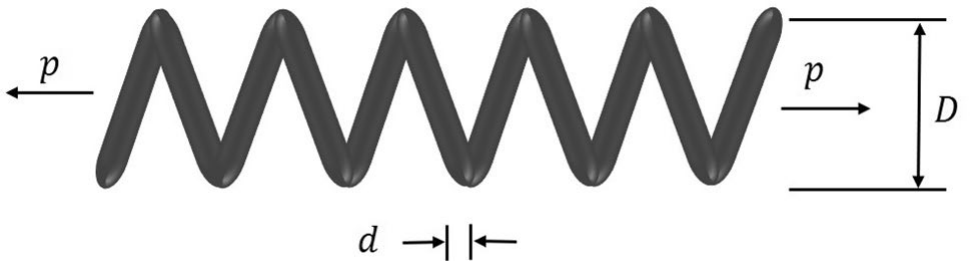
Tension/compression spring design problem.

The tension/compression spring design problem is explained in^98^, and the goal is to reduce the weight of a tension/compression spring. This problem is constrained by minimum deflection, shear stress, surge frequency, outer diameter limits, and design factors. The design factors are the mean coil diameter *D*, the wire diameter *d*, and the number of active coils *N*. Figure 8 illustrates the spring and its properties. The mathematical formulation of this design problem is as follows,

$$\begin{aligned} \text {Consider}\ \vec {z}&= [z_{1}\ z_{2}\ z_{3}] = [d\ D\ N],\\ \text {Minimize}\ f(\vec {z})&= (z_{3}+2) z_{2} z_{1}^2,\\ \text {Subject to}\ g_{1}\ (\vec {z})&= 1-\frac{z_{2}^3 z_{3}}{{71785 z_{1}^4 }} \le 0,\\ g_{2}\ (\vec {z})&= \frac{4z_{2}^2 - z_{1} z_{2}}{{12566 (z_{2}z_{1}^3 - z_{1}^4) }}+\frac{1}{{5108 z_{1}^2 }} \le 0,\\ \end{aligned}$$

$$\begin{aligned} g_{3}\ (\vec {z})&= 1-\frac{140.45 z_{1}}{{z_{2}^2 z_{3} }} \le 0,\\ g_{4}\ (\vec {z})&= \frac{z_{1}+z_{2}}{{1.5}} -1 \le 0,\\ \text {Variable range}\ 0.05&\le z_{1} \le 2.00,\\ 0.25&\le z_{2} \le 1.30,\\ 2.00&\le z_{3} \le 15.0. \end{aligned}$$

Table 26 show the comparison results of the tension/compression spring design problem. Table 27 shows the statistical results of optimization algorithms for the tension/compression spring design problem compared with different algorithms in terms of the mean, standard deviation, minimum, maximum, and median. The results shows that LCA has provided the solution to this problem with optimal values for variables of (5.566E-02, 4.591E-01, 7.194E+00) and an optimal solution of 1.250E-02. The simulation results show that the LCA is superior when compared with other competitor algorithms by providing a better solution and better statistical indicators.

**Table 26:** The comparison results of the tension/compression spring design problem.

Algorithms	Optimal values for variables			Optimum weight
	${\text {d}}$	${\text {D}}$	${\text {N}}$	
LCA	5.566E–02	4.591E–01	7.194E+00	1.250E–02
TSA	5.446E–02	4.256E–01	8.221E+00	1.290E–02
SSA	6.779E–02	1.039E+00	1.240E+00	3.231E+13
MVO	6.903E–02	9.348E–01	2.000E+00	1.782E–02
SCA	5.548E–02	4.536E–01	7.311E+00	1.300E–02
GWO	5.419E–02	4.197E–01	8.379E+00	1.279E–02
WOA	5.714E–02	5.025E–01	6.030E+00	1.317E–02
GJO	5.702E–02	4.984E–01	6.133E+00	1.318E–02
IGWO	5.118E–02	3.444E–01	1.206E+01	1.268E–02
MWOA	5.859E–02	5.467E–01	5.179E+00	1.347E–02
MTBO	5.327E–02	3.958E–01	9.328E+00	1.273E–02
BWO	5.000E–02	3.119E–01	1.500E+01	1.326E–02
HHO	6.223E–02	6.672E–01	3.624E+00	1.453E–02
MGO	5.000E–02	3.104E–01	1.500E+01	1.319E–02
SCSO	5.833E–02	5.385E–01	5.322E+00	1.342E–02

**Table 27:** Statistical results of optimization algorithms in the tension/compression spring problem.

${\text {Algorithms}}$	${\text {Mean}}$	${\text {Std}}$	${\text {Minimum}}$	${\text {Maximum}}$	${\text {Median}}$
LCA	1.260E–02	4.342E–04	1.250E–02	1.444E–02	1.250E–02
TSA	1.281E–02	7.965E–05	1.269E–02	1.297E–02	1.281E–02
SSA	5.422E+14	3.864E+14	2.230E+13	9.908E+14	6.224E+14
MVO	1.653E–02	2.016E–03	1.277E–02	1.818E–02	1.759E–02
SCA	1.303E–02	1.399E–04	1.283E–02	1.321E–02	1.302E–02
GWO	1.273E–02	7.459E–05	1.268E–02	1.303E–02	1.272E–02
WOA	1.395E–02	1.134E–03	1.268E–02	1.654E–02	1.360E–02
GJO	1.275E–02	1.024E–04	1.269E–02	1.318E–02	1.273E–02
IGWO	1.269E–02	7.246E–06	1.267E–02	1.270E–02	1.269E–02
MWOA	1.381E–02	8.973E–04	1.268E–02	1.611E–02	1.353E–02
MTBO	1.271E–02	3.626E–05	1.267E–02	1.281E–02	1.270E–02
BWO	1.422E–02	2.034E–03	1.294E–02	1.905E–02	1.322E–02
HHO	1.368E–02	1.047E–03	1.267E–02	1.687E–02	1.339E–02
MGO	1.340E–02	1.326E–03	1.268E–02	1.777E–02	1.272E–02
SCSO	1.314E–02	4.994E–04	1.270E–02	1.421E–02	1.287E–02

### Pressure vessel design problem

**Figure 9:**
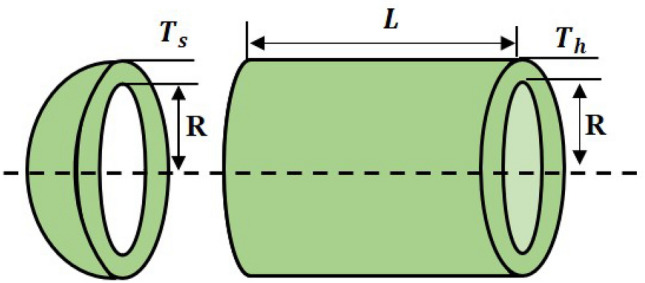
Pressure vessel design problem.

The idea is to produce a pressure vessel design with the least cost. Figure 9 illustrates the pressure vessel and the design parameters. This problem has four variables: shell thickness ($T_s$), head thickness ($T_h$), inner radius (*R*), and length of the cylindrical section excluding the head (*L*). This design problem is mathematically deposited as follows:

$$\begin{aligned} \text {Consider}\ \vec {z}&= [{z}_{1}\ {z}_{2}\ {z}_{3}\ {z}_{4} ] = [T_s\ T_h\ R\ L],\\ \text {Minimize}\ f(\vec {z})&= 0.6224{z}_{1} {z}_{3} {z}_{4}+1.7781 {z}_{2} {z}_{3}^2 +3.1661 {z}_{1}^2 {z}_{4} +19.84 {z}_{1}^2 {z}_{3},\\ \text {Subject to}\ g_{1}\ (\vec {z})&= -{z}_{1}+0.0193{z}_{3} \le 0,\\ g_{2}\ (\vec {z})&= - {z}_{1}+0.00954 {z}_{3} \le 0,\\ g_{3}\ (\vec {z})&= -\pi {z}_{3}^2 {z}_{4}-\frac{4}{3} \pi {z}_{3}^2 +1,296,000 \le 0,\\ g_{4}\ (\vec {z})&= {z}_{4} - 240 \le 0,\\ \text {Variable range}\ 0&\le {z}_{1} \le 99,\\ 0&\le {z}_{2} \le 99,\\ 10&\le {z}_{3} \le 200,\\ 10&\le {z}_{4} \le 20. \end{aligned}$$

**Table 28:** The comparison results of the pressure vessel problem.

Algorithms	Optimal values for variables				Optimum weight
	$T_s$	$T_h$	*R*	*L*	
LCA	1.406E+00	2.712E+00	6.178E+01	2.609E+01	5.886E+03
TSA	7.857E–01	3.940E–01	4.035E+01	2.000E+02	5.972E+03
SSA	6.955E+00	3.033E+01	7.386E+01	5.460E+00	3.676E+05
MVO	1.187E+00	5.890E–01	6.135E+01	2.952E+01	7.125E+03
SCA	8.637E–01	4.103E–01	4.285E+01	2.000E+02	7.054E+03
GWO	7.817E–01	3.875E–01	4.045E+01	1.982E+02	5.902E+03
WOA	1.297E+00	5.923E–01	6.184E+01	2.543E+01	7.497E+03
GJO	1.232E+00	6.052E–01	6.342E+01	1.805E+01	7.202E+03
IGWO	7.786E–01	3.853E–01	4.034E+01	1.998E+02	5.888E+03
MWOA	8.149E–01	5.384E+00	4.204E+01	1.774E+02	2.163E+04
MTBO	7.842E–01	3.876E–01	4.063E+01	1.958E+02	5.897E+03
BWO	9.974E–01	5.062E–01	4.948E+01	1.033E+02	6.678E+03
HHO	9.434E–01	4.619E–01	4.841E+01	1.115E+02	6.262E+03
MGO	8.139E–01	4.023E–01	4.217E+01	1.757E+02	5.949E+03
SCSO	1.245E+00	6.155E–01	6.451E+01	1.312E+01	7.259E+03

Table 28 show comparison results of the pressure vessel problem. Table 29 shows the statistical results of optimization algorithms for the pressure vessel problem compared with different algorithms in terms of the mean, standard deviation, minimum, maximum, and median. The results show that LCA has provided the solution to this problem with optimal values for variables of (1.406E+00, 2.712E+00, 6.178E+0, and 2.609E+01) and an optimal solution of 5.886E+03. The simulation results show that the LCA is superior when compared with other competitor algorithms by providing a better solution and better statistical indicators.

**Table 29:** Statistical results of optimization algorithms in the pressure vessel problem.

${\text {Algorithms}}$	${\text {Mean}}$	${\text {Std}}$	${\text {Minimum}}$	${\text {Maximum}}$	${\text {Median}}$
LCA	5.887E+03	9.632E–01	5.886E+03	5.890E+03	5.886E+03
TSA	6.357E+03	5.103E+02	5.918E+03	7.357E+03	6.097E+03
SSA	6.290E+05	4.818E+05	3.281E+04	1.731E+06	5.432E+05
MVO	7.009E+03	4.792E+02	6.031E+03	7.839E+03	7.146E+03
SCA	7.085E+03	6.383E+02	6.062E+03	8.359E+03	7.045E+03
GWO	6.026E+03	3.505E+02	5.892E+03	7.267E+03	5.908E+03
WOA	8.340E+03	1.747E+03	6.078E+03	1.378E+04	7.950E+03
GJO	6.422E+03	5.740E+02	5.911E+03	7.339E+03	6.061E+03
IGWO	5.890E+03	2.219E+00	5.887E+03	5.895E+03	5.889E+03
MWOA	9.650E+03	3.756E+03	6.064E+03	2.163E+04	8.196E+03
MTBO	6.008E+03	2.992E+02	5.885E+03	7.233E+03	5.916E+03
BWO	7.287E+03	5.733E+02	6.480E+03	8.450E+03	7.141E+03
HHO	6.754E+03	4.730E+02	6.016E+03	7.535E+03	6.676E+03
MGO	6.544E+03	5.746E+02	5.886E+03	7.319E+03	6.377E+03
SCSO	6.672E+03	4.893E+02	5.890E+03	7.315E+03	6.873E+03

### Welded beam design problem

**Figure 10:**
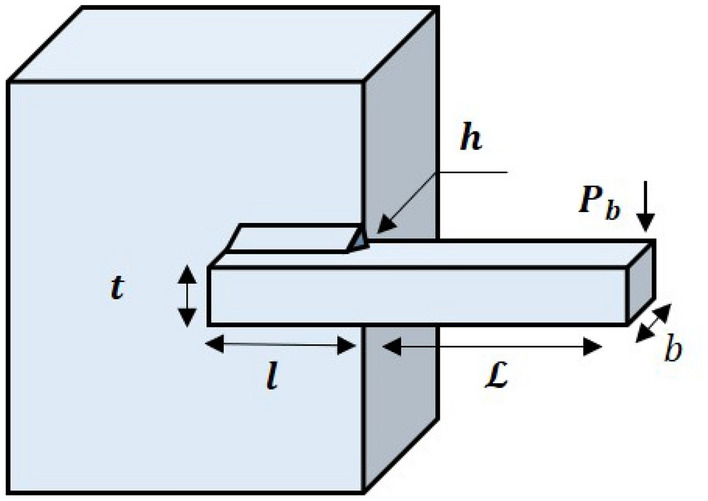
Welded beam design problem.

A popular welded beam design^99^ is given in Fig. 10 to examine the demonstration of LCA in the engineering area. The goal is to discover the optimal design factors for reducing the total manufacturing cost of a welded beam exposed to bending stress ($\sigma$), shear stress ($\tau$), beam end deflection ($\delta$), the bar’s buckling load ($P_c$) and other constraints. This problem has four variables: weld thickness (*h*), bar length (*l*), height (*t*), and thickness (*b*). This design problem is mathematically deposited as follows:

$$\begin{aligned} \text {Consider}\ \vec {z}&= [{z}_{1}\ {z}_{2}\ {z}_{3}\ {z}_{4} ] = [h\ l\ t\ b],\\ \text {Minimize}\ f(\vec {z})&= 1.10471 {z}_{1}^2 {z}_{2}+0.04811 {z}_{3} {z}_{4} (14.0+{z}_{2}),\\ \text {Subject to}\ g_{1}\ (\vec {z})&= \tau (\vec {z})-\tau _{max} \le 0,\\ g_{2}\ (\vec {z})&= \sigma (\vec {z})-\sigma _{max} \le 0,\\ g_{3}\ (\vec {z})&= \delta (\vec {z})-\delta _{max} \le 0,\\ g_{4}\ (\vec {z})&= {z}_{1} - {z}_{4} \le 0,\\ g_{5}\ (\vec {z})&= P - {P}_{c}(\vec {z}) \le 0,\\ g_{6}\ (\vec {z})&= 0.125 - {z}_{1} \le 0,\\ g_{7}\ (\vec {z})&= 1.1047{z}_{1}^2 - 0.04811{z}_{3}{z}_{4} (14.0+{z}_{2})-5.0 \le 0,\\ \text {Variable range}\ 0.1&\le {z}_{1} \le 2,\\ 0.1&\le {z}_{2} \le 10,\\ 0.1&\le {z}_{3} \le 10,\\ 0.1&\le {z}_{4} \le 2,\\ \text {where}\ \tau (\vec {z})&= \sqrt{({\tau }^{'})^{2}+2{\tau }^{'}{\tau }^{''} \frac{{z}_{2} }{2R}+({\tau }^{''})^{2}},\\ {\tau }^{'}&=\frac{P}{\sqrt{2}{z}_{1}{z}_{2}},\\ {\tau }^{''}&=\frac{MR}{J},\\ M&=P(L+\frac{{z}_{2}}{2}),\\ R&=\sqrt{\frac{{z}_{2}^2}{4}+(\frac{{z}_{1}+{z}_{3}}{2})^{2}},\\ J&=2\{\sqrt{2}{z}_{1} {z}_{2} [ \frac{{z}_{2}^{2}}{4}+(\frac{{z}_{1}+{z}_{3}}{2})^2 ] \},\\ \sigma (\vec {z})&=\frac{6PL}{{z}_{4} {z}_{3}^{2}},\\ \delta (\vec {z})&=\frac{6P{L}^3}{E{z}_{3}^{2} {z}_{4}},\\ {P}_{c}(\vec {z})&=\frac{4.013E\sqrt{\frac{{z}_{3}^{2}{z}_{4}^{6}}{36}}}{L^2} (1-\frac{{z}_{3}}{2L} \sqrt{\frac{E}{4G}} ),\\ P&=6000lb,\\ L&= 14in,\\ \delta _{max}&= 0.25in,\\ E&= 30 \times 10^{6} psi,\\ G&= 12 \times 10^{6} psi,\\ \tau _{max}&= 13,600 psi,\\ \sigma _{max}&= 13,600 psi. \end{aligned}$$

Table 30 show the comparison results of the pressure vessel problem. Table 31 shows the statistical results of optimization algorithms for the pressure vessel problem compared with different algorithms in terms of the mean, standard deviation, minimum, maximum, and median. The results show that LCA has provided the solution to this problem with optimal values for variables of (1.922E-01, 6.087E+00, 9.008E+00, and 2.087E-01) and an optimal solution of 1.715E+00. The simulation results show that the LCA is superior when compared with other competitor algorithms by providing a better solution and better statistical indicators.

**Table 30:** The comparison results of the welded beam design problem.

Algorithms	Optimal values for variables				Optimum weight
	*h*	*l*	*t*	*b*	
LCA	1.922E–01	6.087E+00	9.008E+00	2.087E–01	1.715E+00
TSA	1.953E–01	3.776E+00	8.992E+00	2.082E–01	1.760E+00
SSA	4.527E–01	1.820E+00	1.801E+00	1.494E+00	6.137E+24
MVO	2.031E–01	3.446E+00	9.266E+00	2.046E–01	1.748E+00
SCA	1.875E–01	4.230E+00	8.807E+00	2.210E–01	1.872E+00
GWO	2.056E–01	3.477E+00	9.040E+00	2.057E–01	1.726E+00
WOA	1.779E–01	6.451E+00	8.464E+00	2.345E–01	2.179E+00
GJO	2.000E–01	3.599E+00	9.038E+00	2.059E–01	1.734E+00
IGWO	2.057E–01	3.472E+00	9.037E+00	2.057E–01	1.725E+00
MWOA	2.391E–01	3.080E+00	8.411E+00	2.392E–01	1.848E+00
MTBO	2.057E–01	3.470E+00	9.037E+00	2.057E–01	1.725E+00
BWO	1.490E–01	5.884E+00	9.085E+00	2.081E–01	1.952E+00
HHO	1.835E–01	4.461E+00	8.816E+00	2.162E–01	1.858E+00
MGO	1.872E–01	3.691E+00	9.590E+00	2.031E–01	1.801E+00
SCSO	1.927E–01	3.777E+00	9.037E+00	2.058E–01	1.745E+00

**Table 31:** Statistical results of optimization algorithms in the welded beam design problem.

${\text {Algorithms}}$	${\text {Mean}}$	${\text {Std}}$	${\text {Minimum}}$	${\text {Maximum}}$	${\text {Median}}$
LCA	1.720E+00	2.248E–02	1.715E+00	1.815E+00	1.715E+00
TSA	1.743E+00	7.388E–03	1.730E+00	1.760E+00	1.742E+00
SSA	4.839E+24	2.868E+24	1.623E+24	1.321E+25	4.140E+24
MVO	1.746E+00	1.140E–02	1.728E+00	1.767E+00	1.744E+00
SCA	1.868E+00	4.178E–02	1.782E+00	1.934E+00	1.869E+00
GWO	1.728E+00	2.257E–03	1.726E+00	1.736E+00	1.727E+00
WOA	2.337E+00	4.210E–01	1.902E+00	3.505E+00	2.195E+00
GJO	1.731E+00	4.011E–03	1.726E+00	1.741E+00	1.730E+00
IGWO	1.725E+00	9.715E–05	1.725E+00	1.725E+00	1.725E+00
MWOA	2.161E+00	3.825E–01	1.802E+00	3.336E+00	2.073E+00
MTBO	1.725E+00	2.840E–06	1.725E+00	1.725E+00	1.725E+00
BWO	2.262E+00	3.469E–01	1.804E+00	2.930E+00	2.234E+00
HHO	1.889E+00	1.338E–01	1.745E+00	2.329E+00	1.848E+00
MGO	1.800E+00	1.238E–01	1.726E+00	2.253E+00	1.759E+00
SCSO	1.730E+00	5.926E–03	1.725E+00	1.745E+00	1.729E+00

### Speed reducer design problem

**Figure 11:**
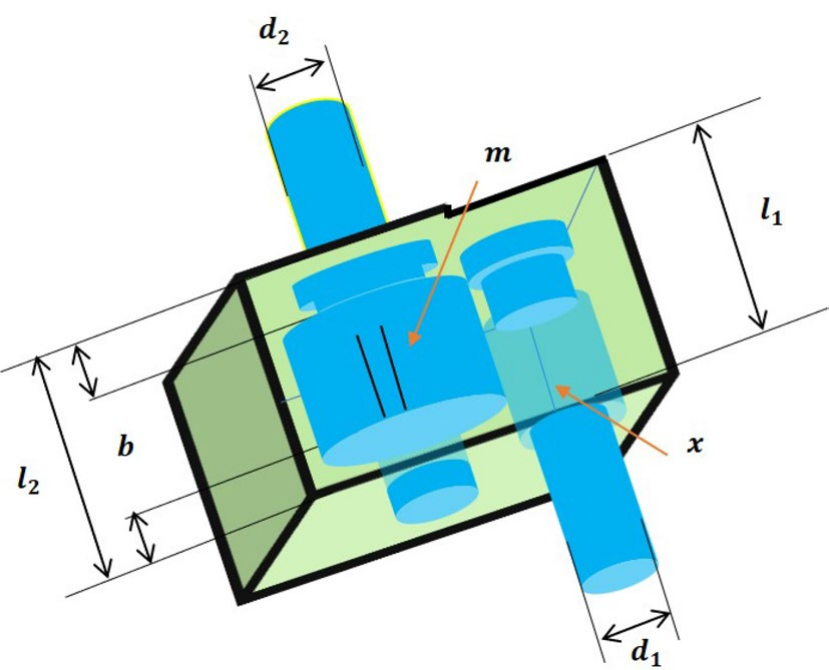
Speed reducer design problem.

The speed reducer^100^, is a crucial component of the gearbox in mechanical systems and has a wide range of uses. In this optimisation problem, the weight of the speed reducer has to be lowered with 11 constraints (Fig. 11). Seven variables make up this problem, such as *b*, *m*, *x*, $l_1$, $l_2$, $d_1$, and $d_2$. This design problem is mathematically deposited as follows:

$$\begin{aligned} \text {Consider}\ \vec {z}&= [{z}_{1}\ {z}_{2}\ {z}_{3}\ {z}_{4}\ {z}_{5}\ {z}_{6}\ {z}_{7}] = [b\ m\ x\ l_1\ l_2\ d_1\ d_2],\\ \text {Minimize}\ f(\vec {z})&= 0.7854{z}_{1} {z}_{2}^{2} (3.3333{z}_{3}^{2}+14.9334{z}_{3}-43.0934)-1.508{z}_{1}({z}_{6}^{2}+{z}_{7}^{2})\\+&7.4777({z}_{6}^{2}+{z}_{7}^{2})+ 0.7854({z}_{4}{z}_{6}^{2}+{z}_{5}{z}_{7}^{2}),\\ \text {Subject to}\ g_{1}\ (\vec {z})&= \frac{27}{{z}_{1}{z}_{2}^{2} {z}_{3}}-1 \le 0,\\ g_{2}\ (\vec {z})&= \frac{397.5}{{z}_{1}{z}_{2}^{2} {z}_{3}^{2}}-1 \le 0,\\ g_{3}\ (\vec {z})&= \frac{1.93{z}_{4}^{3}}{{z}_{2}{z}_{6}^{4} {z}_{3}}-1 \le 0,\\ g_{4}\ (\vec {z})&= \frac{1.93{z}_{5}^{3}}{{z}_{2}{z}_{7}^{4} {z}_{3}}-1 \le 0,\\ g_{5}\ (\vec {z})&= \frac{\sqrt{(\frac{745 {z}_{4}}{{z}_{2}{z}_{3}})^{2}+16.9 \times 10^{6}}}{110{z}_{6}^{3}}-1 \le 0,\\ g_{6}\ (\vec {z})&= \frac{\sqrt{(\frac{745 {z}_{5}}{{z}_{2}{z}_{3}})^{2}+157.5 \times 10^{6}}}{85{z}_{7}^{3}}-1 \le 0,\\ g_{7}\ (\vec {z})&= \frac{{z}_{2} {z}_{3}}{40}-1 \le 0,\\ g_{8}\ (\vec {z})&= \frac{5{z}_{2} }{{z}_{1} }-1 \le 0,\\ g_{9}\ (\vec {z})&= \frac{{z}_{1} }{12{z}_{2} }-1 \le 0,\\ g_{10}\ (\vec {z})&= \frac{1.5{z}_{6}+1.9}{{z}_{4}}-1 \le 0,\\ g_{11}\ (\vec {z})&= \frac{1.1{z}_{7}+1.9}{{z}_{5}}-1 \le 0,\\ \text {Variable range}\ 2.6&\le {z}_{1} \le 3.6,\\ 0.7&\le {z}_{2} \le 0.8,\\ {z}_{3}&\in \{17, 18, 19,....,28 \},\\ 7.3&\le {z}_{4},\\ {z}_{5}&\le 8.3,\\ 2.9&\le {z}_{6} \le 3.9,\\ 5&\le {z}_{7} \le 5.5. \end{aligned}$$

Table 32 show the comparison results of the speed reducer design problem. Table 33 shows the statistical results of optimization algorithms for the speed reducer design problem compared with different algorithms in terms of the mean, standard deviation, minimum, maximum, and median. The results show that LCA has provided the solution to this problem with optimal values for variables of (3.502E+00, 7.000E-01, 2.517E+01, 8.230E+00, 8.300E+00, 3.811E+00, and 5.457E+00) and an optimal solution of 2.990E+03. The simulation results show that the LCA is superior when compared with other competitor algorithms by providing a better solution and better statistical indicators.

**Table 32:** The comparison results of the speed reducer design problem.

Algorithms	Optimal values for variables							Optimum weight
	*b*	*m*	*x*	$l_1$	$l_2$	$d_1$	$d_2$	
LCA	3.502E+00	7.000E–01	2.517E+01	8.230E+00	8.300E+00	3.811E+00	5.457E+00	2.990E+03
TSA	3.515E+00	7.014E–01	1.701E+01	7.300E+00	7.799E+00	3.441E+00	5.301E+00	3.042E+03
SSA	3.490E+00	2.624E+00	2.807E+00	2.811E+00	3.406E+00	3.562E+00	3.439E+00	1.868E+16
MVO	3.501E+00	7.000E–01	1.700E+01	7.397E+00	7.905E+00	3.395E+00	5.287E+00	3.012E+03
SCA	3.517E+00	7.000E–01	1.700E+01	7.300E+00	8.300E+00	3.388E+00	5.500E+00	3.165E+03
GWO	3.504E+00	7.000E–01	1.700E+01	7.751E+00	7.841E+00	3.353E+00	5.288E+00	3.005E+03
WOA	3.600E+00	7.145E–01	1.768E+01	7.869E+00	7.718E+00	3.547E+00	5.287E+00	3.288E+03
GJO	3.512E+00	7.000E–01	1.700E+01	8.219E+00	8.140E+00	3.414E+00	5.287E+00	3.034E+03
IGWO	3.500E+00	7.000E–01	1.700E+01	7.300E+00	7.715E+00	3.350E+00	5.287E+00	2.994E+03
MWOA	3.500E+00	7.000E–01	1.700E+01	7.913E+00	7.821E+00	3.660E+00	5.335E+00	3.121E+03
MTBO	3.500E+00	7.000E–01	1.700E+01	7.300E+00	7.715E+00	3.350E+00	5.287E+00	2.994E+03
BWO	3.600E+00	7.000E–01	1.700E+01	7.300E+00	8.300E+00	3.361E+00	5.330E+00	3.077E+03
HHO	3.559E+00	7.000E–01	1.749E+01	7.300E+00	8.184E+00	3.426E+00	5.287E+00	3.134E+03
MGO	3.500E+00	7.000E–01	1.700E+01	7.300E+00	7.715E+00	3.350E+00	5.287E+00	2.994E+03
SCSO	3.500E+00	7.000E–01	1.700E+01	8.244E+00	8.072E+00	3.357E+00	5.287E+00	3.013E+03

**Table 33:** Statistical results of optimization algorithms in the speed reducer design problem.

${\text {Algorithms}}$	${\text {Mean}}$	${\text {Std}}$	${\text {Minimum}}$	${\text {Maximum}}$	${\text {Median}}$
LCA	2.991E+03	8.927E–01	2.990E+03	2.994E+03	2.990E+03
TSA	3.043E+03	9.916E+00	3.020E+03	3.058E+03	3.042E+03
SSA	1.968E+16	2.224E+15	1.641E+16	2.423E+16	1.930E+16
MVO	3.042E+03	1.998E+01	3.012E+03	3.094E+03	3.044E+03
SCA	3.117E+03	4.468E+01	3.050E+03	3.191E+03	3.110E+03
GWO	3.007E+03	4.017E+00	2.998E+03	3.013E+03	3.009E+03
WOA	3.247E+03	2.981E+02	3.019E+03	4.379E+03	3.161E+03
GJO	3.016E+03	6.880E+00	3.005E+03	3.034E+03	3.015E+03
IGWO	2.994E+03	4.440E–06	2.994E+03	2.994E+03	2.994E+03
MWOA	3.414E+03	6.050E+02	3.038E+03	5.256E+03	3.181E+03
MTBO	2.994E+03	1.348E–12	2.994E+03	2.994E+03	2.994E+03
BWO	3.078E+03	3.639E+01	3.030E+03	3.191E+03	3.074E+03
HHO	3.361E+03	4.559E+02	3.006E+03	4.492E+03	3.142E+03
MGO	2.994E+03	8.013E–13	2.994E+03	2.994E+03	2.994E+03
SCSO	3.006E+03	5.300E+00	2.997E+03	3.018E+03	3.006E+03

### Gear train design problem

**Figure 12:**
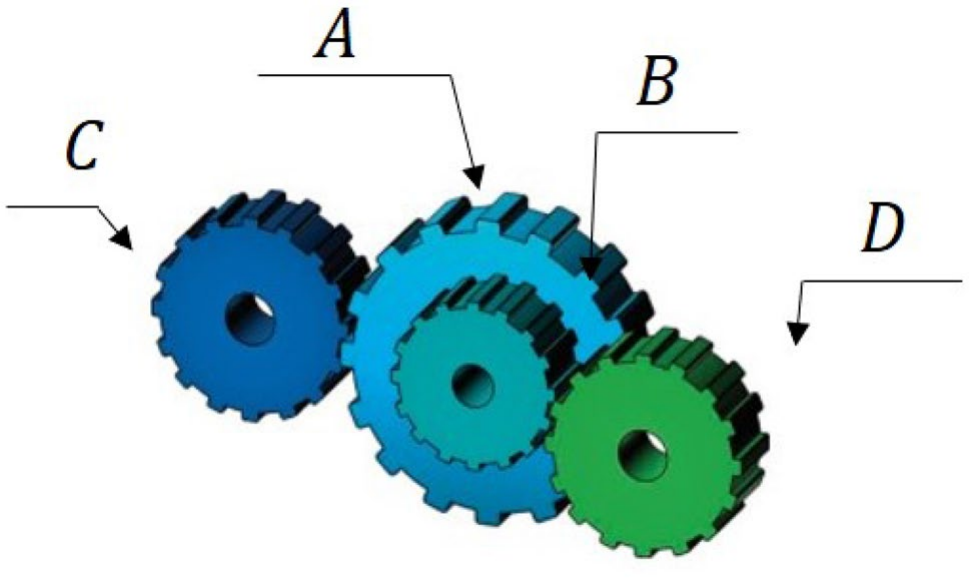
Gear train design problem.

Sandgren proposed the gear train design issue as an unconstrained discrete design problem in mechanical engineering^101^. The purpose of this benchmark task is to reduce the gear ratio, which is defined as the ratio of the output shaft’s angular velocity to the input shaft’s angular velocity. The design variables are the number of teeth of the gears $\eta _{A} (z_{1})$, $\eta _{B} (z_{2})$, $\eta _{C} (z_{3})$,and $\eta _{D} (z_{4})$, and Fig. 12 illustrates the 3D model of this problem. The mathematical formulation of the gear train design problem is as follows,

$$\begin{aligned} \text {Consider}\ \vec {z}&= [z_{1}\ z_{2}\ z_{3}\ z_{4}] = [\eta _{A}\ \eta _{B}\ \eta _{C}\ \eta _{D}],\\ \text {Minimize}\ f(\vec {z})&= \left( \frac{1}{{6.931}}-\frac{z_{3}z_{2}}{{z_{1} z_{4}}}\right) ^2,\\ \text {Variable range}\ {}&z_{1}\ z_{2}\ z_{3}\ z_{4} \in \{12, 13, 14, \cdots , 60\}. \end{aligned}$$

The optimization technique involves finding a scenario that minimises or maximises an objective function while fulfilling a predetermined set of constraints. This case is known as the optimal solution, and it is often explored through an exponential collection of candidate solutions requiring highly expensive execution time. Meta–heuristic approximation techniques have been developed to help with this practical challenge. Even though these problem–solving methods cannot guarantee that the solution is optimal, they are quite capable of providing solutions that are close to optimal^1–6^. Meta–heuristic algorithms use exploitation and exploration, which represent intensity and diversity, as their two methods for determining the optimal solution. The growth of meta–heuristic algorithms has been influenced by a variety of natural phenomena, including animals, insects, wildlife, birds, living things, plants, biomedical laws, chemical reactions, physics laws, human activities, game mechanics, and other natural biological processes. In general, meta–heuristic algorithms may be divided into five categories: evolutionary–based optimization algorithms, swarm–based optimization algorithms, chemistry and physics–based optimization algorithms, game–based optimization algorithms, and human–based optimization algorithmTable 34 show the comparison results of the gear train design problem. Table 35 shows the statistical results of optimization algorithms for the gear train design problem compared with different algorithms in terms of the mean, standard deviation, minimum, maximum, and median. The results show that LCA has provided the solution to this problem with optimal values for variables (5.593E+01, 1.435E+01, 3.146E+01, and 5.593E+01) and an optimal solution of 0.000E+00. The simulation results show that the LCA is superior when compared with other competitor algorithms by providing a better solution and better statistical indicators.

**Table 34:** Gear train design comparison results.

Algorithms	Optimal values for variables				Optimum weight
	$z_{1}$	$z_{2}$	$z_{3}$	$z_{4}$	
LCA	5.593E+01	1.435E+01	3.146E+01	5.593E+01	0.000E+00
TSA	5.535E+01	2.621E+01	1.723E+01	5.657E+01	9.901E–14
SSA	1.462E+01	2.251E+01	1.656E+01	5.790E+01	8.772E–02
MVO	4.592E+01	3.163E+01	1.257E+01	6.000E+01	8.957E–13
SCA	5.772E+01	1.203E+01	1.419E+01	2.049E+01	8.291E–10
GWO	5.037E+01	2.648E+01	1.434E+01	5.227E+01	1.240E–11
WOA	5.503E+01	3.185E+01	1.364E+01	5.470E+01	0.000E+00
GJO	5.778E+01	3.096E+01	1.200E+01	4.456E+01	5.081E–12
IGWO	5.468E+01	1.369E+01	2.879E+01	4.998E+01	8.180E–13
MWOA	5.546E+01	1.252E+01	1.995E+01	3.122E+01	0.000E+00
MTBO	5.906E+01	1.228E+01	3.253E+01	4.689E+01	1.360E–14
BWO	6.000E+01	4.328E+01	1.200E+01	6.000E+01	6.305E–12
MGO	5.308E+01	2.214E+01	1.371E+01	3.965E+01	7.861E–18
SCSO	2.829E+01	1.258E+01	1.200E+01	3.700E+01	1.112E–14

**Table 35:** Statistical results of optimization algorithms in the gear train design problem.

${\text {Algorithms}}$	${\text {Mean}}$	${\text {Std}}$	${\text {Minimum}}$	${\text {Maximum}}$	${\text {Median}}$
LCA	5.670E–37	8.886E–37	0.000E+00	1.890E–36	0.000E+00
TSA	4.218E–12	6.905E–12	1.196E–14	2.871E–11	1.140E–12
SSA	1.268E–02	2.703E–02	4.163E–06	9.163E–02	1.420E–03
MVO	2.579E–13	4.637E–13	1.320E–16	1.626E–12	2.829E–14
SCA	2.396E–10	3.358E–10	1.881E–12	1.257E–09	9.631E–11
GWO	1.184E–12	2.826E–12	2.113E–16	1.240E–11	2.005E–13
WOA	1.110E–20	4.965E–20	0.000E+00	2.220E–19	0.000E+00
GJO	2.547E–12	3.648E–12	4.165E–14	1.575E–11	7.313E–13
IGWO	1.134E–12	2.367E–12	1.322E–15	1.025E–11	1.671E–13
MWOA	1.002E–33	4.301E–33	0.000E+00	1.926E–32	0.000E+00
MTBO	1.793E–12	3.475E–12	1.366E–15	1.400E–11	2.271E–13
BWO	1.728E–11	3.593E–11	8.147E–16	1.561E–10	6.227E–12
MGO	1.064E–14	4.462E–14	0.000E+00	2.001E–13	8.414E–17
SCSO	3.212E–14	4.096E–14	1.554E–16	1.485E–13	1.457E–14

### Three-bar truss design problem

**Figure 13:**
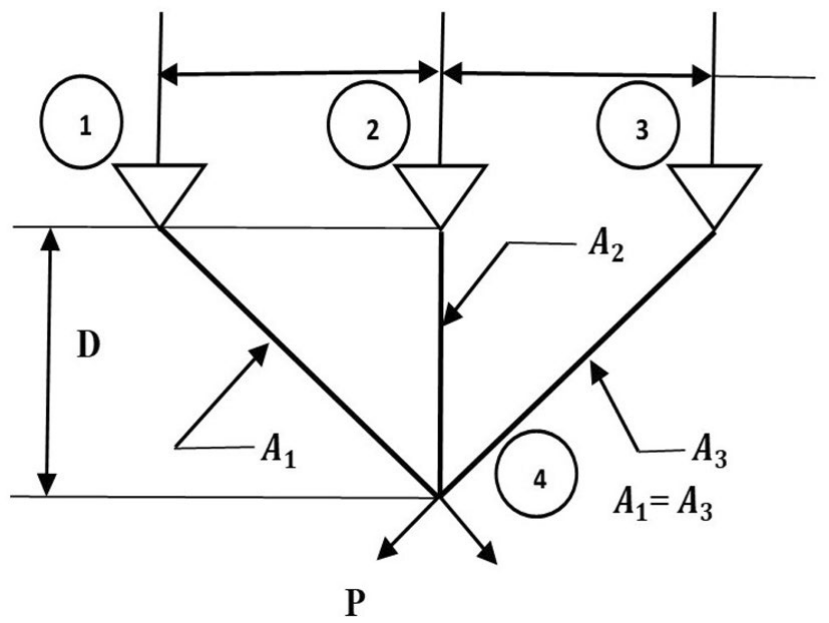
Three bar truss design.

Figure 13 illustrates a three-bar planar truss construction in this scenario. The volume of a statically loaded 3-bar truss must be reduced while stress ($\sigma$) constraints on each truss member are maintained. The aim is to find the best cross-sectional areas, $A_{1} (z_{1})$ and $A_{2} (z_{2})$. The mathematical formulation of this design problem is as follows,

$$\begin{aligned} \text {Consider}\ \vec {z}&= [z_{1}\ z_{2}] = [A_{1}\ A_{2}],\\ \text {Minimize}\ f(\vec {z})&= (2 \sqrt{2z_{1}}+z_{2}) \times l,\\ \text {Subject to}\ g_{1}\ (\vec {z})&= \frac{\sqrt{2z_{1}}+z_{2}}{\sqrt{2z_{1}^2}+2z_{1}z_{2}} P-\sigma \le 0,\\ g_{2}\ (\vec {z})&= \frac{z_{2}}{\sqrt{2z_{1}^2}+2z_{1}z_{2}} P-\sigma \le 0,\\ g_{3}\ (\vec {z})&= \frac{1}{\sqrt{2z_{2}}+z_{1}} P-\sigma \le 0,\\ l&= 100cm, P=2kN/(cm)^3, \sigma =2kN/(cm)^3,\\ \text {Variable range}\ 0&\le z_{1},z_{1} \le 1. \end{aligned}$$

**Table 36:** Three-bar truss design problem comparison results.

Algorithms	Optimal values for variables		Optimum weight
	$A_{1}$	$A_{2}$	
LCA	0.792680179	0.397975679	263.86
TSA	0.789039937	0.40727114	263.90131
SSA	0.749512637	0.612228097	273.216997
MVO	0.78779013	0.410765112	263.8972085
SCA	0.783705109	0.423643793	264.0296582
GWO	0.787712318	0.410980116	263.8967004
WOA	0.86287696	0.230560507	267.1145106
GJO	0.78727585	0.412232522	263.8984891
IGWO	0.788943126	0.407497555	263.8965692
MWOA	0.820466033	0.32502011	264.5648493
MTBO	0.788707389	0.408157403	263.8958776
BWO	0.791251712	0.401863693	263.9861498
HHO	0.807337613	0.3578668	264.1362405
MGO	0.784793549	0.419339298	263.907066
SCSO	0.786133928	0.415485186	263.9007712

**Table 37:** Statistical results of optimization algorithms in the three-bar truss design problem.

${\text {Algorithms}}$	${\text {Mean}}$	${\text {Std}}$	${\text {Minimum}}$	${\text {Maximum}}$	${\text {Median}}$
LCA	263.8619	0.008497058	263.86	263.898	263.86
TSA	263.9073	0.009841	263.898	263.9364	263.9036
SSA	275.892	7.0198	264.2431	288.0811	274.2911
MVO	263.8971	0.0016379	263.8959	263.9018	263.8965
SCA	264.9815	4.2053	263.9165	282.8427	264.0167
GWO	263.8994	0.0030419	263.8964	263.9073	263.8986
WOA	265.2	2.156	263.9012731	271.2815	264.1145
GJO	264.8532	4.2343	263.8967	282.8427	263.9034
IGWO	263.8966	0.00046767	263.896	263.898	263.8965
MWOA	264.8673	0.81709	263.896003	266.1737	264.9071
MTBO	263.8959	0.000059573	263.8958453	263.8961	263.8959
BWO	264.3462	0.35421	263.9852444	265.4122	264.301
HHO	263.9755	0.10863	263.8960107	264.2455	263.9352
MGO	263.9192	0.029241	263.8958743	263.9868	263.9047
SCSO	263.8976	0.0016558	263.8959234	263.901	263.897

Table 36 show the three-bar truss design problem comparison results. Table 37 shows the statistical results of optimization algorithms for the three-bar truss design problem compared with different algorithms in terms of the mean, standard deviation, minimum, maximum, and median. The results show that LCA has provided the solution to this problem with optimal values for variables of (0.792680179 and 0.397975679) and an optimal solution of 263.86. The simulation results show that the LCA is superior when compared with other competitor algorithms by providing a better solution and better statistical indicators.

### Cantilever beam design problem

**Figure 14:**
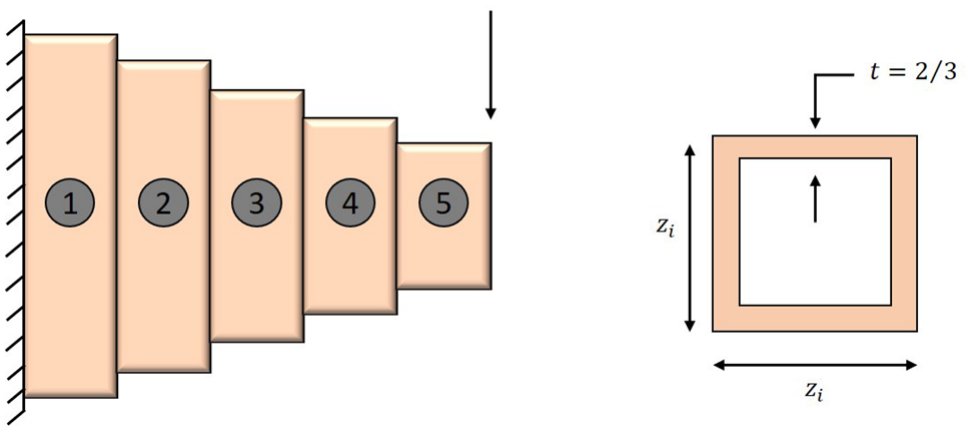
Cantilever beam design problem.

This problem belongs to the category of concrete engineering problems^102^. By maximizing the hollow square cross-section specifications, the overall weight of a cantilever beam is minimized. Figure 14 illustrates how the cantilever’s free node is subjected to a vertical force while the beam is tightly supported at one end. Five hollow square blocks of constant thickness make up the beam; their heights (or widths) are the decision variables, while the thickness remains constant (in this case, 2/3). The mathematical formulation of this design problem is as follows:

$$\begin{aligned} \text {Consider}\ \vec {z}&= [z_{1}\ z_{2}\ z_{3}\ z_{4}\ z_{5}],\\ \text {Minimize}\ f(\vec {z})&= 0.0624(z_{1}+z_{2}+z_{3}+z_{4}+z_{5}),\\ \text {Subject to}\ g\ (\vec {z})&= \frac{61}{z_{1}^3}+\frac{37}{z_{2}^3}+\frac{19}{z_{3}^3}+\frac{7}{z_{4}^3}+\frac{1}{z_{5}^3}-1 \le 0,\\ \text {Variable range}\ {}&0.01 \le z_{i} \le 100, i=1,2,\cdots , 5. \end{aligned}$$

**Table 38:** Cantilever beam comparison results.

Algorithms	Optimal values for variables					Optimum weight
	$z_{1}$	$z_{2}$	$z_{3}$	$z_{4}$	$z_{5}$	
LCA	5.627E+00	5.392E+00	4.439E+00	3.433E+00	3.204E+00	1.3289
TSA	6.070E+00	5.335E+00	4.420E+00	3.552E+00	2.129E+00	1.3419
SSA	1.116E+01	3.392E+01	1.373E+01	5.215E+01	9.018E+00	7.4865
MVO	5.951E+00	5.432E+00	4.589E+00	3.453E+00	2.069E+00	1.3412
SCA	5.531E+00	4.942E+00	4.841E+00	3.842E+00	3.101E+00	1.3888
GWO	5.998E+00	5.315E+00	4.483E+00	3.503E+00	2.175E+00	1.3400
WOA	4.660E+00	1.078E+01	5.578E+00	3.112E+00	3.376E+00	1.7160
GJO	5.975E+00	5.320E+00	4.463E+00	3.542E+00	2.177E+00	1.3401
IGWO	6.016E+00	5.331E+00	4.481E+00	3.488E+00	2.158E+00	1.3399
MWOA	6.616E+00	4.950E+00	5.635E+00	3.890E+00	1.568E+00	1.4139
MTBO	6.022E+00	5.306E+00	4.495E+00	3.495E+00	2.156E+00	1.3399
BWO	5.853E+00	5.425E+00	4.707E+00	3.252E+00	2.383E+00	1.3491
HHO	5.870E+00	5.187E+00	4.596E+00	3.649E+00	2.202E+00	1.3419
MGO	6.001E+00	5.286E+00	4.509E+00	3.525E+00	2.153E+00	1.3399
SCSO	6.061E+00	5.279E+00	4.455E+00	3.518E+00	2.162E+00	1.3400

Table 38 show the cantilever beam problem comparison results. Table 39 shows the statistical results of optimization algorithms for the cantilever beam problem compared with different algorithms in terms of the mean, standard deviation, minimum, maximum, and median. The results show that LCA has provided the solution to this problem with optimal values for variables of (5.627E+00, 5.392E+00, 4.439E+00, 3.433E+00, and 3.204E+00) and an optimal solution of 1.3289. The simulation results show that the LCA is superior when compared with other competitor algorithms by providing a better solution and better statistical indicators.

**Table 39:** Statistical results of optimization algorithms in the cantilever beam.

${\text {Algorithms}}$	${\text {Mean}}$	${\text {Std}}$	${\text {Minimum}}$	${\text {Maximum}}$	${\text {Median}}$
LCA	1.3300	0.00340	1.3289	1.3399	1.3289
TSA	1.341	0.00042	1.3403	1.342	1.3412
SSA	8.1305	2.0829	3.8461	12.1496	7.9834
MVO	1.3407	0.00039	1.3401	1.3417	1.3407
SCA	1.3891	0.02061	1.346	1.4164	1.3925
GWO	1.34	0.000032	1.34	1.3401	1.34
WOA	1.4884	0.10368	1.3467	1.7161	1.4777
GJO	1.3401	0.00013	1.34	1.3405	1.3401
IGWO	1.34	0.000016	1.34	1.34	1.34
MWOA	1.5018	0.1022	1.3607	1.763	1.4724
MTBO	1.34	1.701E-06	1.3399	1.34	1.34
BWO	1.3508	0.0044	1.3433	1.3591	1.3502
HHO	1.3424	0.0012	1.3402	1.3452	1.3424
MGO	1.3404	0.00036	1.3399	1.3411	1.3404
SCSO	1.34	0.000039	1.33996	1.3401	1.34

## Conclusion

This study has introduced a novel human-based meta-heuristic algorithm, namely the Learning Cooking Algorithm, to mimic the food preparation style in our daily lives. The strategies of LCA were mathematically designed in two phases: (i) children learn from their mothers, and (ii) children and mothers learn from a chef. These phases act like the exploration and exploitation mechanisms, which are vital for any meta-heuristic algorithm. The efficiency of the proposed LCA has been tested on 51 benchmark functions and CEC 2019 benchmark functions, and the results have been compared with eminent and top-performing algorithms. The experimental results demonstrate that the proposed algorithm LCA provides a better outcome to an optimization problem by preserving the proper balance between exploration and exploitation. The execution of the LCA algorithm to address seven real-world engineering problems reveals the superior performance of the proposed algorithm. Although LCA has delivered satisfactory results in solving the problems addressed in this paper, there are certain limitations to this approach in solving some multi-modal separable and multi-modal non-separable functions from the 51 benchmark functions and some functions from the CEC 2019 benchmark functions. In future work, this paper suggests several modifications, such as the inclusion of adaptive inertia factors and levy flight distribution, to improve the performance of the proposed LCA algorithm. It also recommends future research on developing a binary and multi-objective version of the LCA algorithm.

## Data Availability

Data and MATLAB code used during the study are available from the corresponding author by request.
